# Gene Expression Profiling of a Hypoxic Seizure Model of Epilepsy Suggests a Role for mTOR and Wnt Signaling in Epileptogenesis

**DOI:** 10.1371/journal.pone.0074428

**Published:** 2013-09-27

**Authors:** Joachim Theilhaber, Sanjay N. Rakhade, Judy Sudhalter, Nayantara Kothari, Peter Klein, Jack Pollard, Frances E. Jensen

**Affiliations:** 1 Translational and Experimental Medicine, Sanofi Cambridge Research Center, Cambridge, Massachusetts, United States of America; 2 Department of Clinical Studies, Genzyme – a Sanofi Company, Cambridge, Massachusetts, United States of America; 3 Department of Pharmacology, University of Virginia, Charlottesville, Virginia, United States of America; 4 Department of Neurology, Perelman School of Medicine, University of Pennsylvania, Philadelphia, Pennsylvania, United States of America; Rutgers University, United States of America

## Abstract

Microarray profiling was used to investigate gene expression in the hypoxic seizure model of acquired epilepsy in the rat, with the aim of characterizing functional pathways which are persistently activated or repressed during epileptogenesis. Hippocampal and cortical tissues were transcriptionally profiled over a one week period following an initial series of seizures induced by mild hypoxia at post-natal day 10 (P10), and the gene expression data was then analyzed with a focus on gene set enrichment analysis, an approach which emphasizes regulation of entire pathways rather than of individual genes. Animals were subjected to one of three conditions: a control with no hypoxia, hypoxic seizures, and hypoxic seizures followed by treatment with the AMPAR antagonist NBQX, a compound currently proposed to be a modulator of epileptogenesis. While temporal gene expression in the control samples was found to be consistent with known processes of neuronal maturation in the rat for the given time window, the hypoxic seizure response was found to be enriched for components of the PI3K/mTOR and Wnt signaling pathways, alongside gene sets representative of glutamatergic, synaptic and axonal processes, perhaps regulated as a downstream consequence of activation of these pathways. Wnt signaling components were also found enriched in the more specifically epileptogenic NBQX-responsive gene set. While activation of the mTOR pathway is consistent with its known role in epileptogenesis and strengthens the case for mTOR or PI3K pathway inhibitors as potential anti-epileptogenic drugs, investigation of the role of Wnt signaling and the effect of appropriate inhibitors might offer a parallel avenue of research toward anti-epileptogenic treatment of epilepsy.

## Introduction

Epilepsy is a widespread neurological disorder affecting as many as 3% of all individuals at some point in their lives, with about 30% of chronic epileptic patients refractory to drugs, and with many patients experiencing apparently progressive forms of the disease [1]. While historically attention has been focused on controlling the acute symptoms of the disease, more recently emphasis has also been placed on understanding the underlying process of epileptogenesis, that is, the molecular and structural changes that occur in brain tissue, sometimes over extended periods of time of months or years, and which eventually lead to the epileptic state [2]. This new “disease-modifying” focus might eventually provide for a more rational approach to treatment of the disorder, in which one deals with the underlying condition rather than just its symptoms, and concomitantly may reveal a greater number and variety of molecular targets for intervention than the ones currently affected by known anti-epileptic drugs.

To help apprehend the molecular changes underlying epilepsy, and hence identify potential molecular targets for intervention, several microarray-based gene expression profiling studies have been conducted in the past few years [3], [4], [5], [6], [7]. These have generally been based on rodent models of epilepsy, in which for instance pilocarpine or kainate were used to induce seizures [8], following which dissected brain tissues were transcriptionally profiled. Taken together, these studies have led to the identification of a large and generally consistent set of genes, differentially regulated in an epileptic context [9]. However, arguably most of these studies measured only the acute transcriptional response to seizures, and did not characterize differential gene expression during epileptogenesis, because profiling was either done shortly after induced seizures, or was performed in models in which epileptogenesis has not been documented as yet. While recent profiling studies have more appropriately addressed epileptogenic processes [10], [11], [12], identifying genes regulated in chronic or longer-term settings, a multiplicity of models will doubtless be required for understanding of the mechanisms underlying epileptogenesis.

In this context, a rodent model using acute hypoxia-induced neonatal seizures in post-natal day 10 (P10) rats to generate long-term chronic epilepsy [13], [14] appeared particularly well suited for a gene expression study of epileptogenesis. This “hypoxic seizure” model, which has already been extensively characterized phenotypically, results in long term increases in neuronal excitability, seizure susceptibility, and eventually gives rise to spontaneous seizures [15]. Furthermore, a number of pharmacological agents, including AMPA receptor antagonist NBQX, and mTOR inhibitor rapamycin [16], [17], [18], have been clearly identified as anti-epileptogenic in this model when they are applied in a critical time-widow following the initial event of hypoxia-induced seizures.

We set out to transcriptionally characterize the hypoxic seizure model, by profiling by microarray over a one week period hippocampal and cortical tissues of a series of rats subjected to an initial event of hypoxic seizures at P10 (with a control series of sham-treated animals generated in parallel). Profiling was also performed on a series of animals in which anti-epileptogenic NBQX treatment was applied following the initial hypoxic seizure event. Brain tissues were collected and profiled at a set of time points from 1 hour to 1 week following the hypoxic seizures, sampling being designed to capture both acute and long-term transcriptional changes in response to the initial seizures, with the expectation that long-term changes in gene expression reflect the ongoing epileptogenic process, and hence may inform on its mechanisms.

Our analysis of the resulting expression data consisted of three parts. First we examined normal, developmentally-regulated changes in gene expression in the control animals. This analysis provided a “reality check” on the experimental system, by confirming induction or repression of genes participating in known neuronal differentiation processes, and also provided a measure of novelty, as the P10–P17 developmental period in the rat had not been previously analyzed by microarray profiling. Second, we examined the differential response to hypoxic seizures, finding a set of genes and associated pathways which participate in the long-term, persistent consequences of hypoxic seizures. Third, we determined the subset of these genes which also respond to NBQX administered after the initial hypoxic event, these genes being potentially more closely implicated in epileptogenic processes. In these analyses, we systematically used gene set enrichment analysis, which emphasized discovery of relevant pathways and functional categories, rather than just of individual genes.

## Results

### Experimental design of the epileptogenesis model

**Figure 1:**
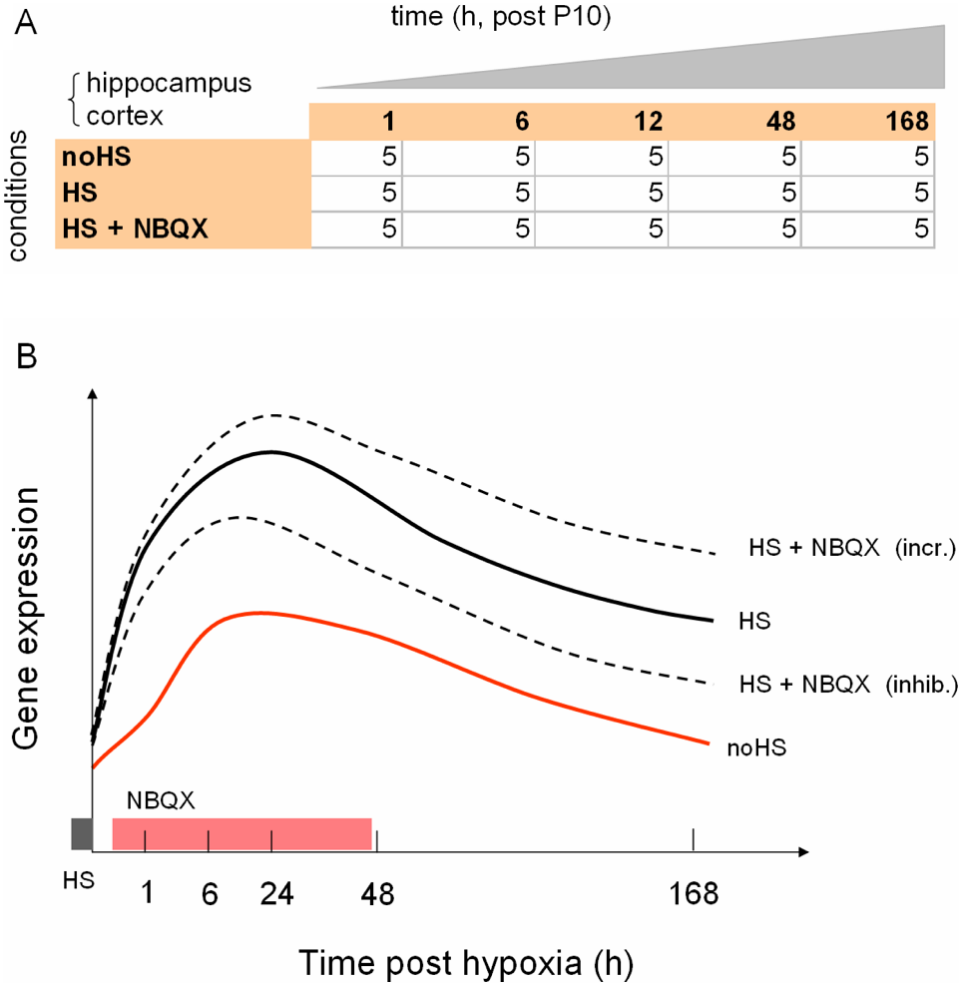
Experimental design for profiling of the hypoxic seizure model. A. Factorial table showing the average number of replicates per combination of treatment condition and sampling time (hours, post P10) for each tissue separately, for a total of 144 samples profiled; noHS  =  no hypoxic seizures induced (baseline condition), HS  =  hypoxic seizures induced at t = 0 h, HS + NBQX  =  hypoxic seizures induced at t = 0 h, followed by four IP administrations of NBQX over a 48 h period. Control vehicle injections were performed at the same times over the same 48 h period under the noHS and HS treatments. B. Cartoon indicating the location of the sampling times and the duration of hypoxic shock (gray bar) and NBQX (rose bar) treatments, alongside hypothetical gene expression profiles for a developmentally modulated gene which also shows persistent activation after hypoxia-induced seizures (HS profile) relative to the baseline (noHS profile). Two possible responses to NBQX treatment are indicated (increase or decrease).

The experimental design of the model for hypoxia-induced epileptogenesis is shown in Figure 1A. Cohorts of P10 rat pups were subjected to one of three experimental conditions: 1) a baseline control treatment, with no induction of hypoxic seizures (noHS), 2) a treatment with hypoxic shock inducing seizures (HS) in which each animal underwent a 15 minutes passage at 5% O_2_, and 3) a treatment with hypoxic shock, followed by four intra-peritoneal injections of NBQX over a 48 h period, starting at 30 minutes post-hypoxia (HS+NBQX). Control vehicle injections of DMSO were performed for the animals under the noHS and HS conditions. For the animals undergoing hypoxia, a mean number of 10.6 (se ±2.8) seizures were observed during the 15 minute hypoxic interval. For each treatment condition, cohorts of 4 to 6 rat pups were killed at five sampling times spanning 1 hour to 1 week post-hypoxia (1 h, 6 h, 12 h, 48 h, 168 h post-hypoxia), for a total of 75 animals sacrificed. Whole cortex and hippocampi were extracted from the sacrificed animals and separately profiled on two Affymetrix high-throughput plate microarrays (see Methods), resulting in a total of 142 gene expression profiles after quality-control (File S1). The data for the study have been deposited in the Gene Expression Omnibus [19], under data set GSE44903.

As a guide for the data analyses, we used the cartoon in Figure 1B, which suggests the behavior of a gene under the given experimental conditions. 1) Under baseline conditions with no induction of hypoxic seizures (red line, “noHS”), the gene undergoes solely developmentally-regulated expression. 2) Under the time course following hypoxic seizures (black line, “HS”) the gene is for instance induced relative to baseline, and its expression level remains higher one week later. 3) With in addition NBQX treatment following hypoxic seizures (dashed lines, “HS+NBQX”), two possible outcomes are indicated, differential inhibition (inhi.) or further increase (incr.) of the hypoxic seizure response. The responses indicated in Figure 1B are expected to roughly correspond to effects of decreasing magnitude, with strong modulation due to developmental effects, weaker differential response due to hypoxic seizures, and yet weaker differential response due to additional treatment with NBQX.

### The developmental time course for post P10 neuronal tissues is consistent with known neuronal maturation processes

**Figure 2:**
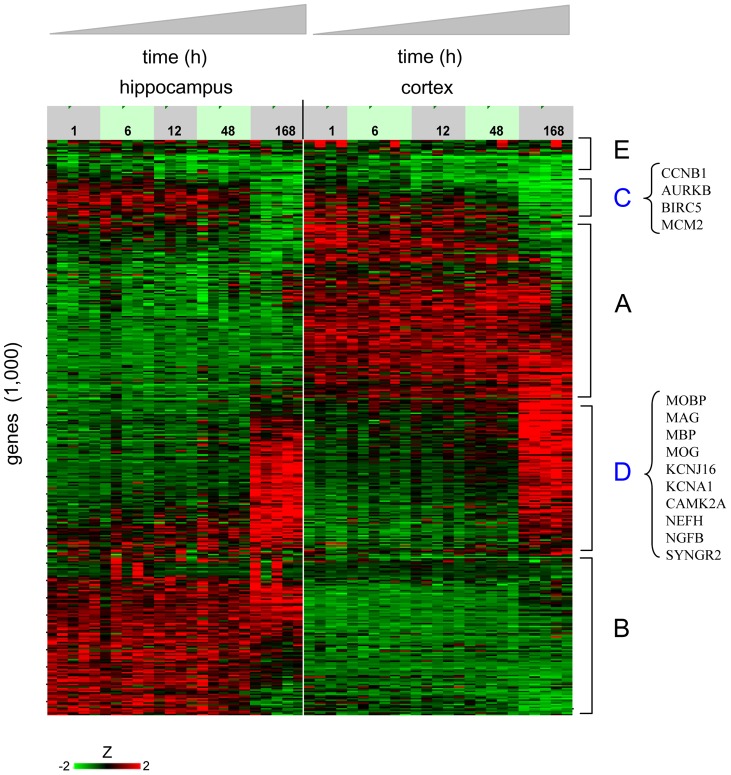
Baseline developmental time courses show tissue-specific as well as common gene expression patterns. Gene expression profiles are displayed for hippocampus (left) and cortex (right), over the time period P10–17. Clustered expression intensities standardized to Z-scores are shown for the thousand genes with most significant variation across all samples. The color scale saturates at Z = ±2. Cluster A: genes with expression specific to cortex; B: genes with expression specific to hippocampus; C: set of genes repressed in time in both tissues, containing many genes for cellular proliferation control; D: set of genes induced in time in both tissues, containing many genes specifying mature neuronal lineages; E: common set of genes with no significant changes of expression.

We first examined the baseline, developmentally-regulated gene expression profiles, as these were obtained for a time interval in neuronal development not generally studied by microarray profiling. Thus in Figure 2, the P10–P17 developmental time courses, for both hippocampal and cortical tissues, are displayed as heat maps based on the thousand genes with most significant variation across all time points and all samples [in the figure, genes correspond to rows and samples to columns, with rows clustered in accordance to the similarity of their expression profiles (see Methods)]. Biological replicates within each group are separately presented in Figure 2, where it can be seen that the multiple profiles corresponding to each time point form stripes of similar color and intensity, indicating that overall good biological replicate concordance was achieved in the experiment.

The profiles in Figure 2 can be approximately grouped into five clusters, A–E. Two of the clusters (A, B) contain genes with expression specific to cortex and hippocampus respectively, with corresponding bright red regions in the heat map occurring specifically for each of these tissues. Two other clusters (C, D, blue lettering), contain genes with common temporal pattern of expression in both tissues, with repression over time occurring in cluster C and induction in cluster D. Casual inspection of these clusters shows that cluster C contains numerous genes associated with cellular proliferation (such as cyclin B1, aurora B kinase or MCM2), with expression strongly down-regulated by the final time point P17. Cluster D contains numerous genes specific to mature neuronal or glial cells (such as neurofilament NEFH, nerve growth factor NFGB, or oligodendrocytic myelination markers MBP or MOG), with expression strongly up-regulated by the final time point P17. The small cluster E contains genes common to both tissues, but which do not show marked temporal changes.

To mathematically quantify which functional pathways are significantly modulated in the developmental time course, we performed a gene set enrichment analysis, using a curated collection of neuronally-relevant gene sets. For each gene set considered, enrichments were computed for each of the profiles of log2 ratios of expression at times 6, 12, 48 and 168 h relative to expression at the 1 h time point, using a “regulated” Kolmogorov-Smirnov (KS) analysis, which generates a P-value, “left” and “right” enrichments scores C_L_ and C_R_, and a distributional plot (KS plot) of log2 ratio values for gene set members relative to the profile being investigated (see Methods).

**Figure 3:**
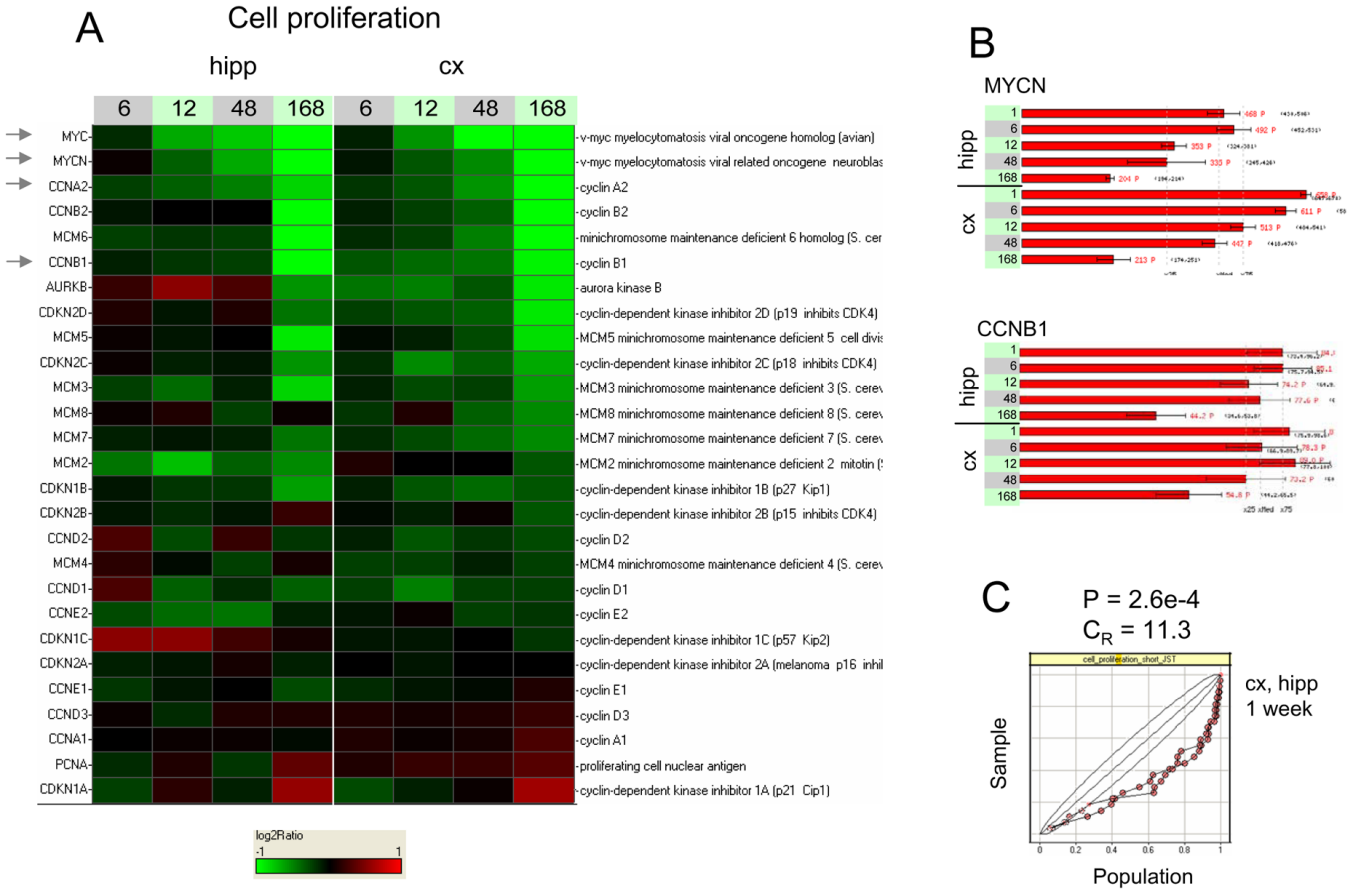
A cellular proliferation gene set shows strong repression under baseline developmental conditions. A. Log2-ratios of gene expression relative to the 1 h time point, in each tissue separately, are displayed as a heat map, with sampling times post-P10 indicated in hours. Colors saturate for log2-ratio  = ±1. Almost all members of this manually curated gene set representing cellular proliferation are repressed over time. B. Detailed intensity profiles for MYCN and cyclin B1 (CCNB1) showing strong repression at t = 1 week. C. KS plot showing the distribution of the log2-ratios at t = 1 week for both tissues. P-value and enrichment are reported for hippocampus only. Hipp  =  hippocampus, cx  =  cortex. The arrows point to some of the genes referred to in the main text.

As already suggested by inspection of Figure 2, gene sets representative of cell cycle were found to be significantly down-regulated over time. This can be seen for a cellular proliferation gene set (Figure 3), where statistically significant (P = 2.6×10^−4^) and large (C_R_ = 11.3) negative enrichment is observed at t = 168 h, with for instance strong down-regulation of MYC, MYCN, cyclin A2 (CCNA2), and mini-chromosome maintenance (MCM) genes; for a mitotic processes gene set (Figure S1 in File S9, P<10^−5^, C_R_ = 9.3 at t = 168 h), with strong down-regulation of chromosomal passenger genes survivin and aurora kinase B and of mitotic cyclins B1 and B2; and finally for a gene set containing targets of the proliferation-inducing sonic-hedgehog [20] (Figure S2 in File S9, P<10^−5^, C_R_ = 12.6 at t = 168 h).

Next focusing on characterization of neuronal lineages, and first looking at gene sets representative of progenitors cells, we found that a gene set for neuronal stem cells was negatively enriched over time (File S9 in File S9, P = 1.6×10^−2^, C_R_ = 8.6 at t = 168 h), with in particular repression of markers nestin and CD24. A related gene set for neuronal and glial cell progenitors (Figure S4 in File S9) showed statistically marginal negative enrichment (P = 6.7×10^−2^) but with overall repression of its markers (C_R_ = 1.9 at t = 168 h), including strong down-regulation of the neuronal precursor migration gene doublecortin (DCX). In logical coordination with the observed repression of gene sets for neuronal precursors, gene sets for the mature neuronal lineages were found to be induced. Thus two overlapping gene sets for neurons (Figure S5, Figure S6 in File S9) showed moderate but statistically significant positive enrichments (P = 2.0×10^−2^, C_L_ = 1.9; P<10^−5^, C_L_ = 2.7 respectively). Consistently with maturation of neurons, a collection of synaptic structure genes (File S7, Figure S7 in File S9) displayed moderate enrichment over one week's time (P = 2.1×10^−2^, C_L_ = 1.6). Most prominent were induction of genes for pre-synaptic proteins such as vesicular glutamate transporter VGLUT1, synapsin 2 and VAMP1, and for post-synaptic scaffolding proteins such as HOMER1 and SHANK3. Developmental changes in gene expression for selected neuronal receptors are further described in File S7 (Figure S10- Figure S12 in File S9).

**Figure 4:**
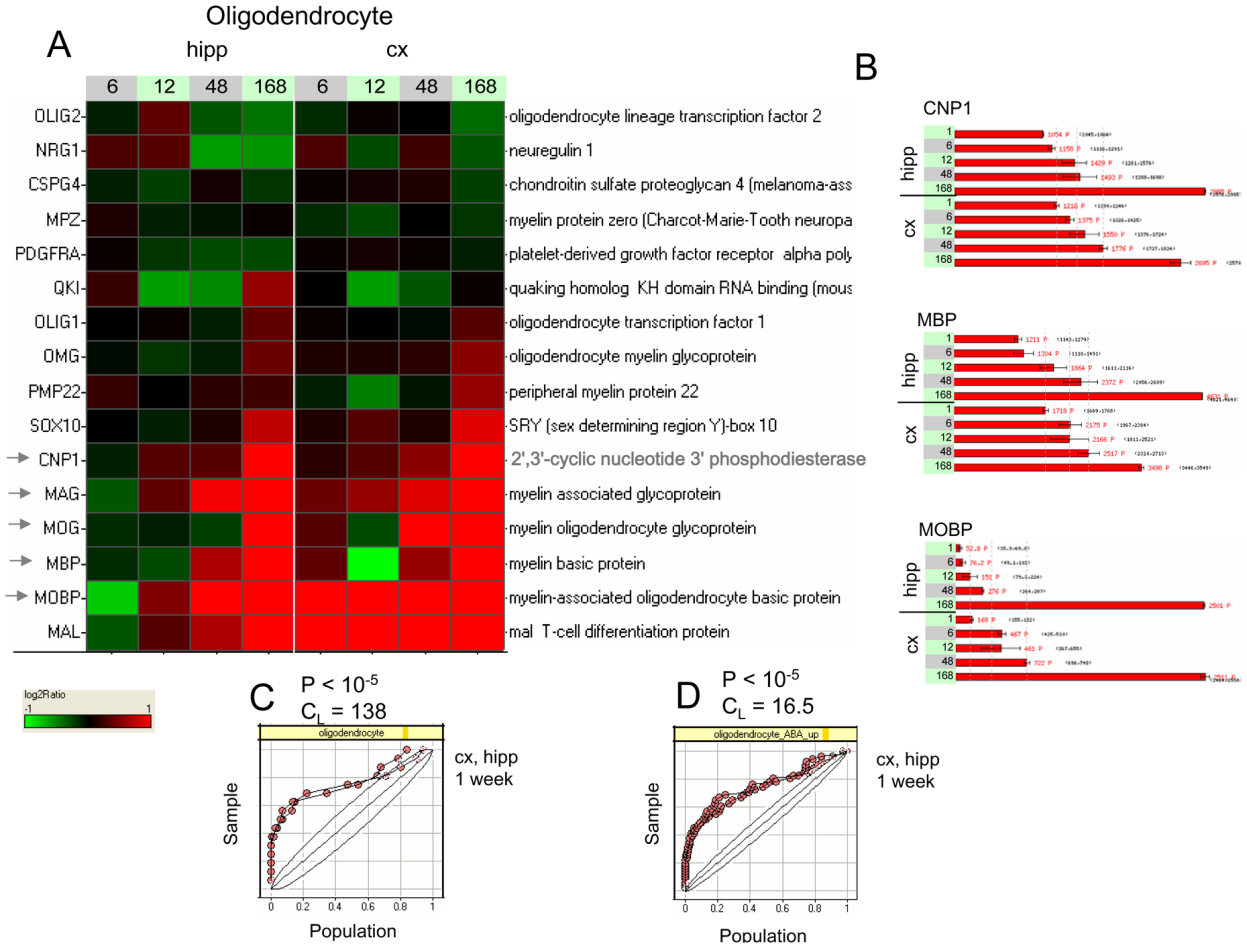
A gene set containing a set of oligodendrocytic markers is strongly induced under baseline developmental conditions. Gene expression profiles are shown for a manually curated set of oligodendrocytic markers. A. Heat map of gene profiles, showing very strong induction of several genes, including myelin basic protein (MBP), myelin associated glycoprotein (MAG), myelin oligodendrocyte glycoprotein (MOG) and myelin-associated oligodendrocyte basic protein (MOBP). B. Detailed intensity profiles for 2′,3′-cyclic nucleotide 3′ phosphodiesterase (CNP1), MBP and MOBP. C. KS plot showing the distribution of the log2-ratios at t = 1 week for both tissues, with very marked leading edges. D. KS plot for an independent empirically determined gene set of oligodendrocytic markers [21] (Figure S9 in File S9), also showing large positive enrichment. See Figure 3 for key to figure color and scale details.

Regarding the glial cell lineages, a gene set containing astrocytic markers (Figure S8 in File S9) was more strongly and positively enriched (P = 1.2×10^−4^, C_L_ = 3.7 at t = 168 h) than any of the neuronal gene sets, with induction of connexin 30 (GJB6), glutamine synthetase (GLUL) and the glutamate transporter GLT-1 driving the enrichment. Finally, gene sets for the oligodendrocytic lineage showed the most dramatic enrichments of all the lineage markers: a manually curated list of oligodendrocytic markers (Figure 4) showed an extremely large, positive enrichment at one week's time (P<10^−5^, C_L_ = 138), driven by very strong and coordinate up-regulation of canonical myelination markers such as MBP, MAG, MOG, and MOBP. An empirically-derived set of oligodendrocytic markers [21] also showed very strong positive enrichment at one week's time (Figure S9 in File S9, P<10^−5^, C_L_ = 16.5).

The down-regulation of genes for cellular proliferation and for neuronal precursors, and the concomitant up-regulation of genes for mature neuronal lineages, as noted above, is consistent with the differentiation processes known to take place over this period of time in both hippocampal and cortical tissues [22]. Furthermore, the relative ranking of gene set enrichments observed here, schematically *C*_neurons_ < *C*_astrocytes_ << *C*_oligodendrocytes_ where *C* denotes enrichment, is consistent with the known order of neuronal maturation processes [22], with neurons at P10 already partially differentiated, glial cells more immature and still strongly differentiating, and myelination processes in oligodendrocytes especially prominent in the time interval P10–P17.

### The hypoxic seizure response gene set is enriched in Wnt and mTOR pathway components

**Figure 5:**
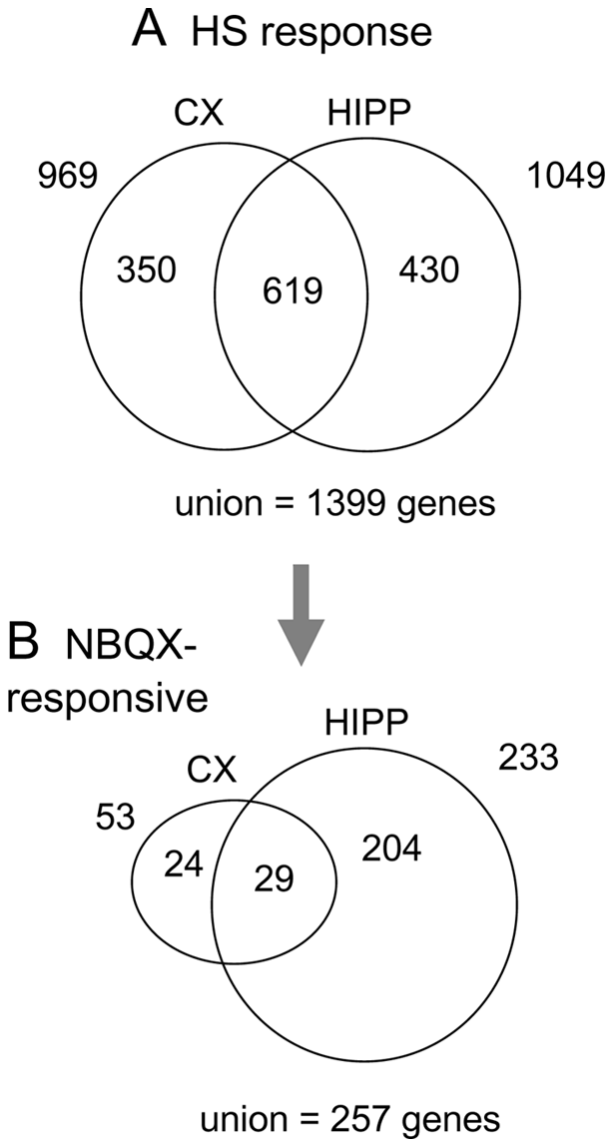
Determination of the hypoxic seizure and of the NBQX responsive sets. A. Hypoxic seizure (HS) response set: Venn diagram showing membership of genes selected separately in the indicated tissues for significance of response to hypoxic seizures (two-way ANOVAs, FDR < = 0.25). A total of 1,399 genes are contained in the HS response set. B. NBQX-responsive set: Venn diagram for the subsets of the HS response which are also NBQX-responsive. The NBQX-responsive group contains 257 genes.

**Figure 6:**
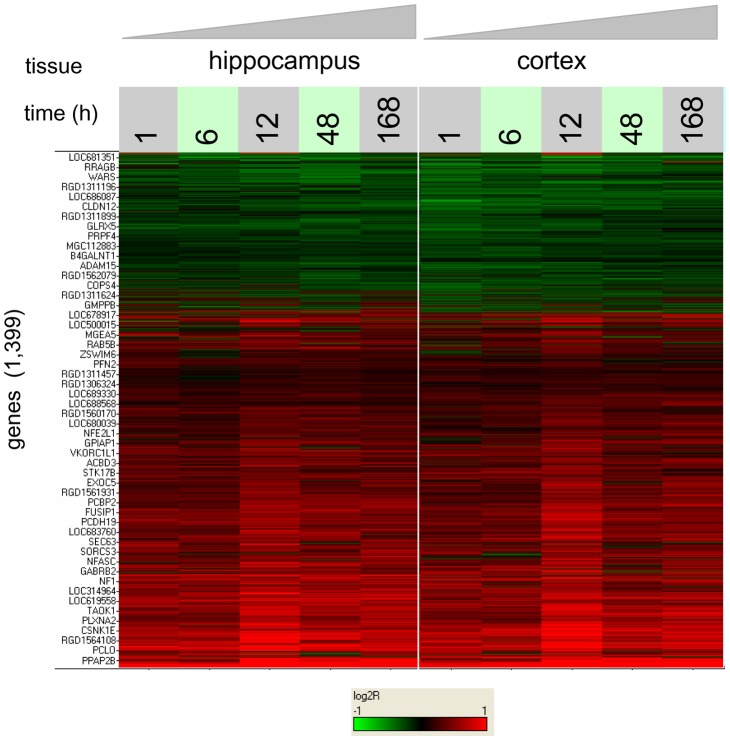
Heat map of gene expression for the hypoxic seizure response set. Clustred log2 ratios of gene expression of hypoxic seizure relative to time-matched normoxic controls, are shown for each tissue for all 1,399 genes in the HS response set. Colors saturate for log2-ratio  = ±1. Maximum differential regulation is observed at 12 h post-HS.

We next examined the set of genes with persistent changes in expression post-hypoxia, relative to the baseline normoxic conditions, these genes defining the “hypoxic seizure (HS) response set”. To that end, two-way analyses of variance were separately performed on the hippocampal and cortical gene expression profiles to find microarray probe sets measuring statistically significant effects under HS relative to baseline conditions, after which probe sets were mapped to genes (see Methods). Sets of 1,049 and 969 genes (out of the total of 14,405 represented on the microarray), were found to be modulated in hippocampus and cortex, respectively, the intersection of the two sets consisting of 619 genes, a highly significant number (P<10^−9^) (Figure 5A). The non-redundant union of the two sets, containing a total of 1,399 genes, defines the HS response set (File S2). A heat map of the clustered, log2-transformed ratios of intensities, HS versus matched normoxic control, for the HS response set is shown in Figure 6. It can be seen that the temporal profile of the differential response is similar for all genes, with early induction at 1 h and moderate peak at 12 h, and is also similar between cortex and hippocampus. Overall about twice as many genes are induced as are repressed.

**Figure 7:**
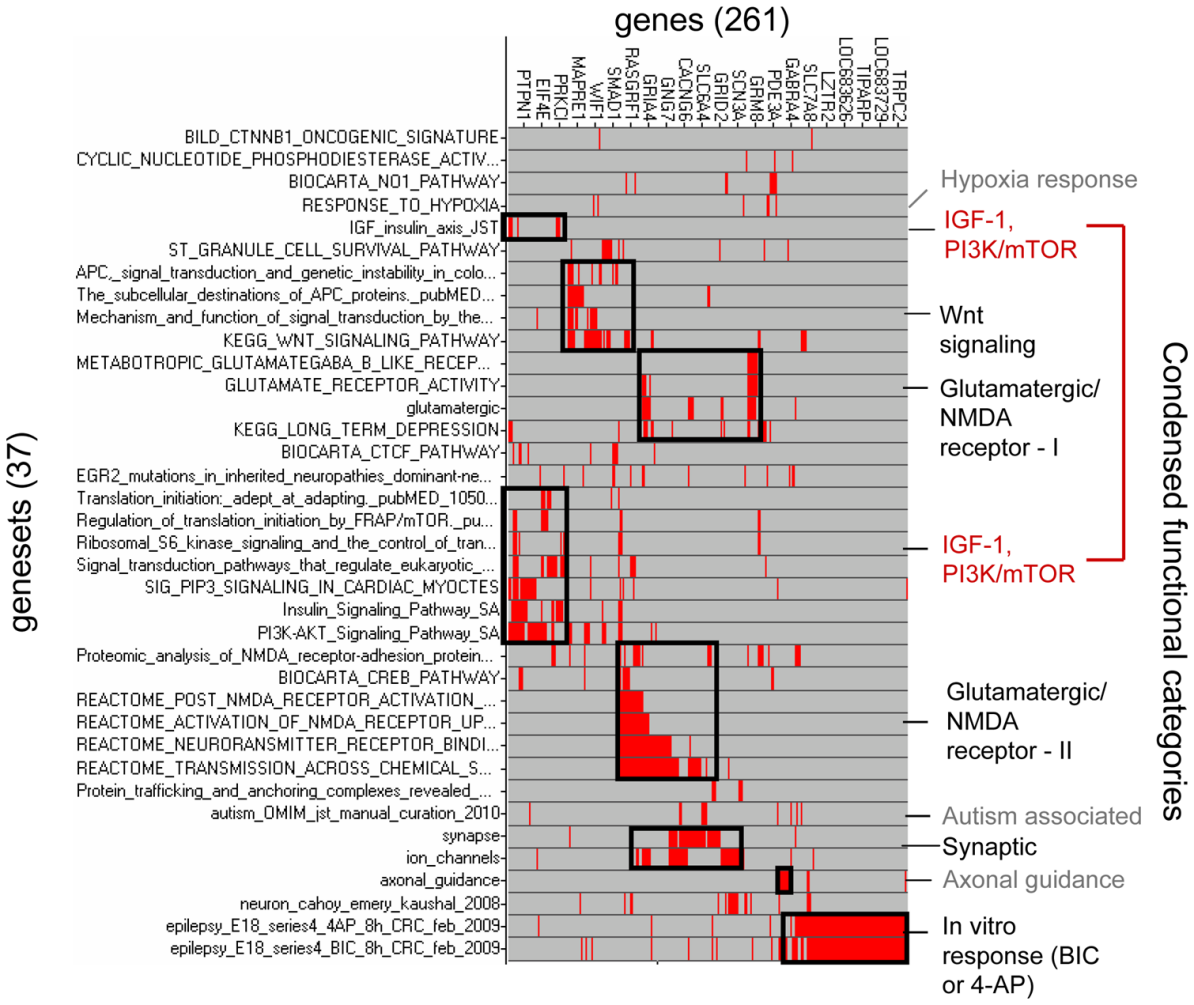
Geneset × gene membership matrix for the hypoxic seizure response. The clustered {geneset × gene} membership matrix for the 37 gene sets and the corresponding 261 leading-edge genes selected by gene set enrichment analysis of the hippocampal hypoxic seizure response at t = 12 hours is shown. Red indicates presence of a gene (column) in given gene set (row), and gray its absence in a given gene set. 9 functional clusters, named on the right-hand-side of the figure, were determined by visual inspection. Note that the IGF-1/PI3K/mTOR “cluster” actually consists of two groups (red lettering).

In order to quantify which biological pathways are affected by hypoxic seizures, we considered the 12 h profile in hippocampus as most representative, because it corresponds to the point of maximum transcriptional modulation for most of the genes (in addition, the 12 h time point is also the one at which maximum activation of glutamatergic signaling components has been previously observed at the protein level [23]). We performed gene set enrichment analysis for the 12 h profile using gene sets arising from five distinct collections containing a total of 6,394 gene sets (see Methods). Collecting results, we found a total of 37 gene sets scoring significantly against the 12 h hippocampal profile under the regulated KS analysis (Table 1, File S3). We then filtered the gene content of this small collection, by retaining only the most significantly changing, “leading-edge” genes in each of the 37 gene sets, and obtaining a final set of 261 genes (see Methods). The corresponding 37×261 gene membership matrix (gene sets versus selected genes, see File S4 and File S5), is displayed as a clustered heat map in Figure 7.

**Table 1:** Gene sets enriched in the response to hypoxic seizures.

Gene set name	Source	ku	kd	P-value	FDR	cLeft	cRight
ion_channels	NC	245	0	2.2E-04	9.3E-04	1.52	1
autism_OMIM_jst_manual_curation_2010	NC	49	0	3.2E-03	5.6E-03	2.08	1
PI3K-AKT_Signaling_Pathway_SA	NC	93	0	0	0	1.94	1
epilepsy_E18_series4_4AP_8h_CRC_feb_2009	NC	253	264	0	0	1.68	1.43
glutamatergic	NC	37	0	4.2E-04	1.2E-03	4.76	1
synapse	NC	86	0	3.3E-03	5.2E-03	1.67	1
epilepsy_E18_series4_BIC_8h_CRC_feb_2009	NC	332	343	0.0E+00	0.0E+00	2.05	1
neuron_cahoy_emery_kaushal_2008	NC	196	0	7.5E-03	8.9E-03	1.72	1
axonal_guidance	NC	57	0	1.3E-03	2.7E-03	2.58	1
Insulin_Signaling_Pathway_SA	NC	91	0	2.4E-02	2.4E-02	1.51	1
IGF_insulin_axis_JST	NC	13	0	1.6E-02	1.7E-02	3.89	1
KEGG_WNT_SIGNALING_PATHWAY	BC	110	0	0	0	2.64	1
BIOCARTA_NO1_PATHWAY	BC	26	0	3.9E-03	5.7E-03	3.49	1
BIOCARTA_CTCF_PATHWAY	BC	19	0	3.3E-03	5.0E-03	4.54	1
KEGG_LONG_TERM_DEPRESSION	BC	57	0	2.3E-03	4.1E-03	2.26	1
BIOCARTA_CREB_PATHWAY	BC	24	0	1.0E-03	2.4E-03	3.85	1
BILD_CTNNB1_ONCOGENIC_SIGNATURE	BC	39	0	2.2E-04	8.4E-04	4.63	1
SIG_PIP3_SIGNALING_IN_CARDIAC_MYOCTES	BC	55	0	5.8E-04	1.6E-03	2.38	1
REACTOME_TRANSMISSION_ACROSS_CHEMICAL_ SYNAPSES	BC	108	0	4.0E-04	1.3E-03	2.12	1
REACTOME_NEURORANSMITTER_RECEPTOR_BINDING_AND_DOWNSTREAM_TRANSMISSION_IN_THE_POSTSYNAPTIC_CELL	BC	68	0	1.1E-03	2.4E-03	2.12	1
REACTOME_POST_NMDA_RECEPTOR_ACTIVATION_EVENTS	BC	28	0	3.3E-03	5.4E-03	3.15	1
REACTOME_ACTIVATION_OF_NMDA_RECEPTOR_UPON_GLUTAMATE_BINDING_AND_POSTSYNAPTIC_EVENTS	BC	32	0	6.0E-05	3.8E-04	6.72	1
ST_GRANULE_CELL_SURVIVAL_PATHWAY	BC	21	0	9.6E-04	2.4E-03	2.61	1
RESPONSE_TO_HYPOXIA	BGO	25	0	6.7E-03	8.5E-03	2.15	1
GLUTAMATE_RECEPTOR_ACTIVITY	BGO	20	0	7.8E-03	9.0E-03	4.52	1
METABOTROPIC_GLUTAMATEGABA_B_LIKE_RECEPTOR_ACTIVITY	BGO	10	0	8.7E-03	9.4E-03	4.52	1
CYCLIC_NUCLEOTIDE_PHOSPHODIESTERASE_ACTIVITY	BGO	12	0	1.2E-02	1.3E-02	3.32	1
EGR2_mutations_in_inherited_neuropathies_dominant-negatively_inhibit_m_pubMED_11394999	PM	79	0	4.2E-03	5.9E-03	2.44	1
Signal_transduction_pathways_that_regulate_eukaryotic_protein_synthesi_pubMED_10521405	PM	77	0	5.3E-03	6.9E-03	2.13	1
Proteomic_analysis_of_NMDA_receptor-adhesion_protein_signaling_complex_pubMED_10862698	PM	65	0	0	0	3.67	1
Regulation_of_translation_initiation_by_FRAP/mTOR._pubMED_11297505	PM	20	0	8.4E-03	9.4E-03	2.78	1
Ribosomal_S6_kinase_signaling_and_the_control_of_translation._pubMED_10579915	PM	21	0	1.0E-04	5.4E-04	2.76	1
Mechanism_and_function_of_signal_transduction_by_the_Wnt/beta-catenin__pubMED_10630639	PM	30	0	6.9E-03	8.4E-03	3.58	1
Translation_initiation:_adept_at_adapting._pubMED_10500305	PM	25	0	5.2E-03	7.1E-03	2.78	1
APC,_signal_transduction_and_genetic_instability_in_colorectal_cancer._pubMED_11900252	PM	19	0	1.4E-03	2.7E-03	4.95	1
The_subcellular_destinations_of_APC_proteins._pubMED_11988767	PM	27	0	1.4E-04	6.7E-04	2.55	1
Protein_trafficking_and_anchoring_complexes_revealed_by_proteomic_anal_pubMED_15024025	PM	13	0	1.2E-03	2.5E-03	2.16	1

The table displays the 37 gene sets out of an initial set of 6,394 which scored significant enrichment against the 12 hour hippocampal response to hypoxic seizures. The gene sets originated from five distinct collections (see Methods), with the source indicated in the second column: NC  =  “neuronal consolidated”, BC  =  “Broad, curated”, BGO  =  “Broad, Gene Ontology”, PM  =  PubMed (no gene sets were selected from the Broad Connectivity Map collection). Keys for column headings: ku  =  number of positive regulated; kd  =  number of negative regulatees; the enrichment factors cLeft and cRight refer to slopes of the distribution functions in the KS plots (see Methods). P-values exactly equal to 0 are equivalent to P-value<10^−5^. A total of 261 leading-edge genes are represented across the selected gene sets.

Inspection of the clusters in Figure 7 suggests that 9 functional groups, named on the right-hand-side, principally contribute to gene set membership. Six of these functional categories, named *Wnt signaling, Glutamatergic/NMDA receptor I* and *II, IGF-1/PI3K/mTOR, Synaptic* and *In vitro response* (black or red lettering), arise from more than one gene set each, while three categories (*Hypoxia response, Axonal* and *Autism associated*)(gray lettering, discussed in File S7, with corresponding Figure S14, Figure S18 and Figure S19 in File S9, respectively), are based on the presence of a single gene set.

The *In vitro response (BIC or 4AP*) group of genes (lower right-hand corner) arise from two gene sets, obtained from analysis of the transcriptional response of cultured rat embryonic E18 cortical cells to induction of seizure-like conditions by the GABA receptor inhibitor bicuculline (BIC) or by the potassium channel blocker 4-aminopyridine (4AP) for 8 h duration (internal communication). The enrichment of these gene sets in the 12 h hippocampal profile (Figure S13 in File S9) indicates that the pathways activated in vivo by hypoxia-induced seizures overlap with those artificially induced in vitro by convulsive agents, despite very different biological contexts. This observation can be considered providing a positive control for the experiment.

The enrichments of the overlapping *Glutamatergic/NMDA receptor I, Glutamatergic/NMDA receptor II* and *Synaptic* categories (Figure S15, Figure S16, Figure S17 in File S9, respectively) suggest modification of synaptic signaling as a result of hypoxic seizures. In the *Glutamatergic/NMDA receptor I* group (Figure S15 in File S9) one finds for instance induction of i) genes coding for the AMPA receptor components GLUR1 through GLUR4 (GRIA1-GRIA4), ii) of genes for the NMDA receptor components NR3A and NR2A (GRIN3A and GRIN2A, respectively), iii) of genes for the astrocytic glutamate transporters GLAST1 (SLC1A3) and GLT1 (SLC1A2), and iv) for the glutamatergic post-synaptic neuron scaffolding components GRIP1 and HOMER1. In the *Glutamatergic/NMDA receptor II* group (Figure S16 in File S9) one finds in addition induction i) of the genes for beta-catenin (CTNNB1) and N-cadherin (CDH2), involved in mediation of Wnt signaling and in cell adhesion, respectively, and ii) induction of the genes coding for CaM kinases CAMK4, CAMK2D, CAMK2G and CAMK2B, which mediate response to changes in cytosolic calcium. The *Synaptic* group (Figure S17 in File S9) includes NLGN3 coding for the pre-synaptic adhesive component neuroligin-3, and gene STX6 coding for syntaxin-6, involved in pre-synaptic vesicular transport.

**Figure 8:**
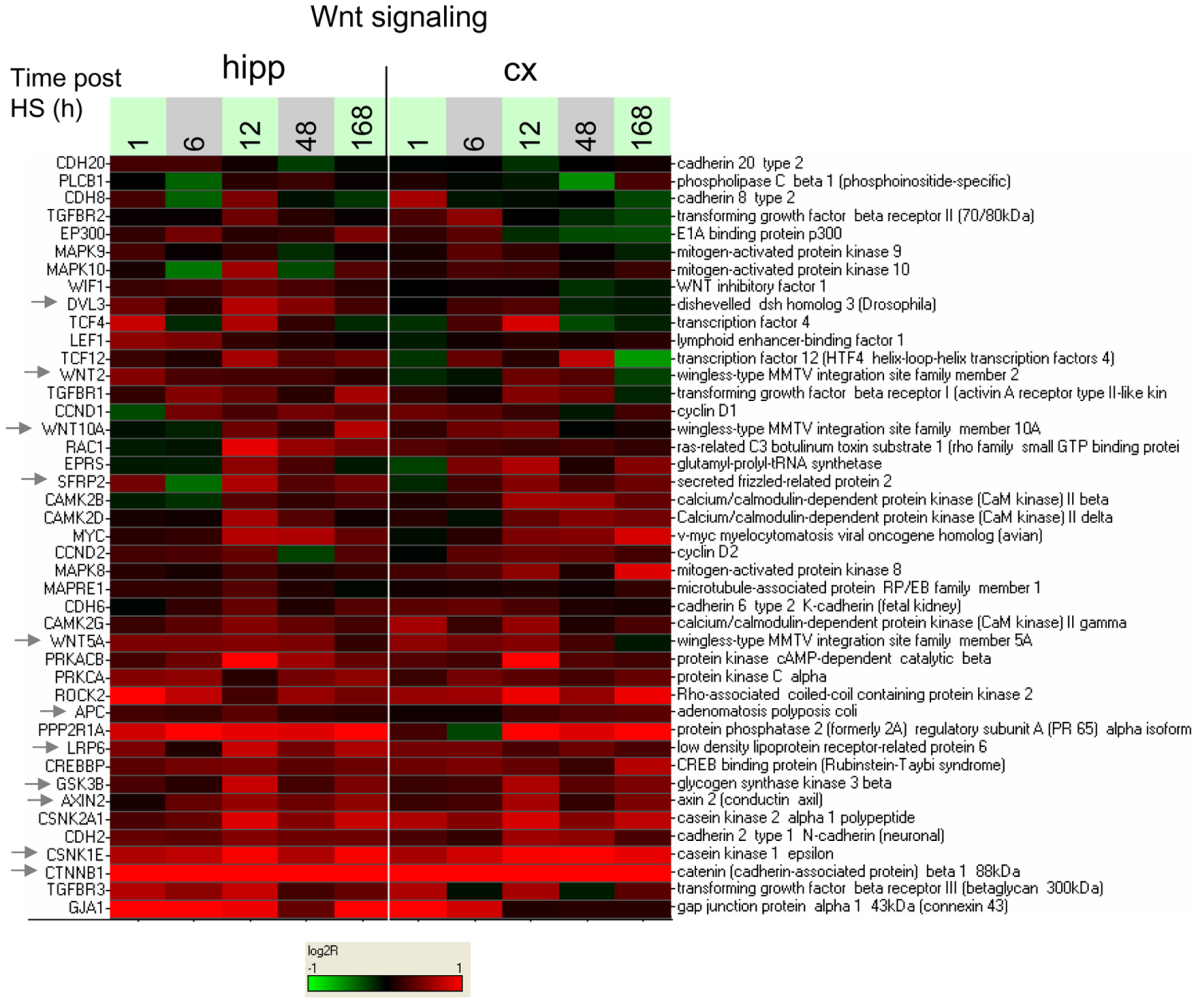
Heat map of response to hypoxic seizures for the genes in the *Wnt signaling* group. Log2-ratios of gene expression of HS to time-matched normoxic controls are displayed for the genes in the *Wnt signaling* group defined by inspection of the enrichment membership matrix of Figure 7. Sampling times post-P10 are indicated in hours. Colors saturate for log2-ratio  = ±1.

**Figure 9:**
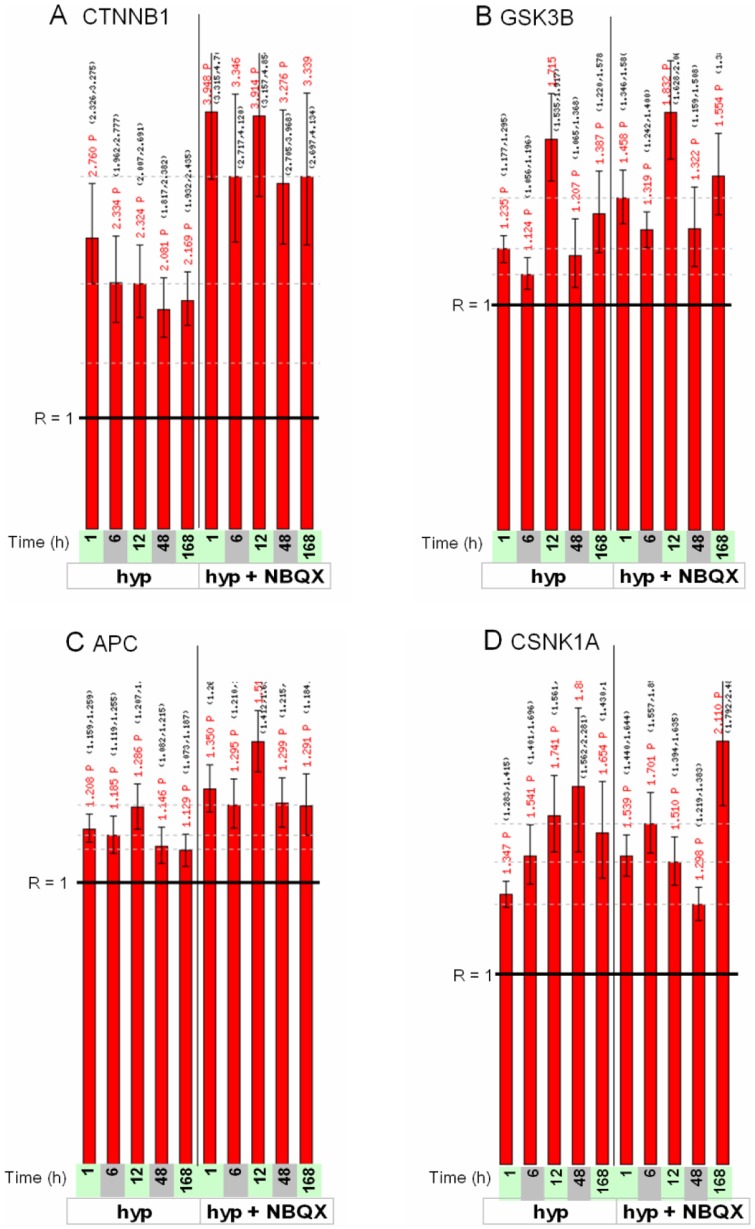
Expression ratios as a function of time for four selected Wnt pathway genes. Gene names are indicated on the top of each figure. In each bar chart, time at the bottom refers to the duration following hypoxic seizures; the left-hand panel (labeled “hyp”) display ratios of hypoxic seizure response to baseline; the right-hand panel (labeled “hyp + NBQX”) displays ratios of response to combined hypoxic seizures and NBQX treatment to baseline. The expression ratio R = 1 (no fold change) is indicated by the black horizontal lines.

Wnt signaling and beta-catenin have important roles in synaptic plasticity [24]–[26], and enrichment of the pathway may reflect synaptic changes in response to hypoxic seizures. Thus, the enriched *Wnt signaling* group (Figure 8) includes the gene for beta-catenin (CTNNB1) already noted above in connection with the *Glutamatergic/NMDA receptor I* group, as well as manifold components of the Wnt signaling pathway including inhibitory protein gene SFRP2 and transducer DVL3, already noted in connection with the *In vitro response (BIC or 4AP)* group, Frizzled co-receptor LRP6, the beta-catenin regulatory complex components CSKN1E, GSK3B, AXIN2, and APC, and genes for Frizzled ligands WNT2, WNT5A and WNT10A. A detailed look at expression ratios for a number of the Wnt pathway genes (Figure 9) indicates a sizeable induction for beta-catenin under HS relative to control (Figure 9A, left-hand profile), with expression ratios above 2 at all time points, alongside milder induction of several other pathway genes considered, with expression ratios in the range 1.2 to 1.5 (Figure 9C–D, left-hand profiles). Additional enrichment analysis with 7 gene sets not included in the analysis above, each containing targets of activated Wnt signaling in different biological contexts (from GeneSigDB [27]) (Table S1), indicated strong enrichment for targets of WNT3A [28] (P = 5.8×10^−3^, C_L_ = 4.95 at t = 12 h) (Figure S20 in File S9).

**Figure 10:**
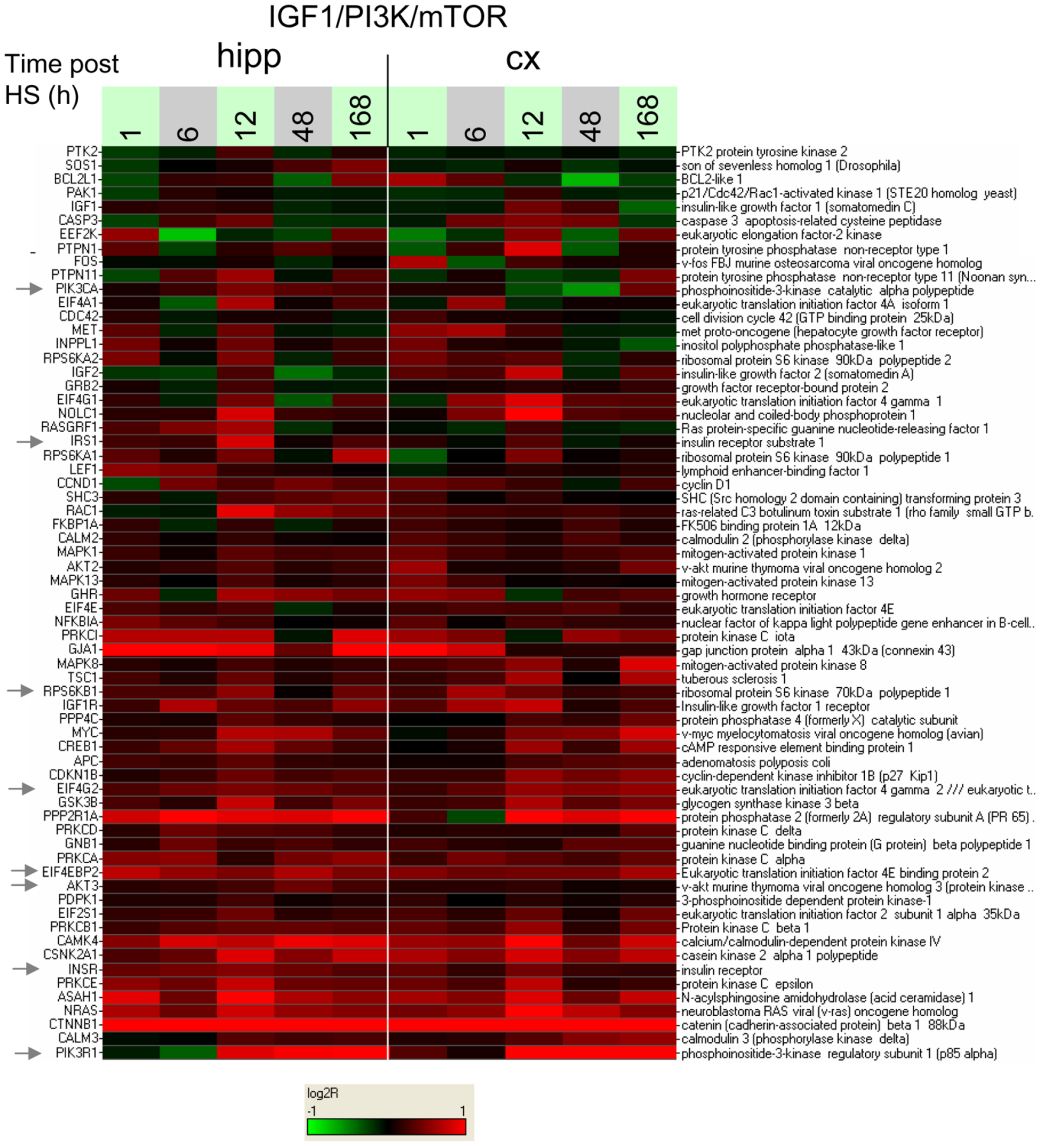
Heat map of response to hypoxic seizures for the genes in the *IGF-1/PI3K/mTOR* group. Log2-ratios of gene expression of HS to time-matched normoxic controls are displayed for the genes in the *IGF-1/PI3K/mTOR* group defined by inspection of the enrichment membership matrix of Figure 7. Sampling times post-P10 are indicated in hours. Colors saturate for log2-ratio  = ±1.

**Figure 11:**
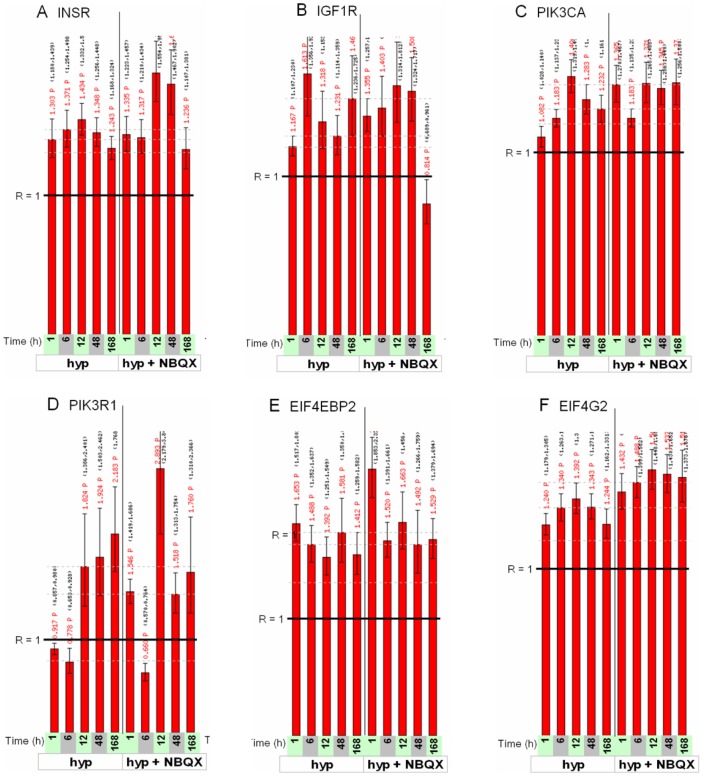
Expression ratios as a function of time for selected IGF-1/PI3K/mTOR pathway genes. Gene names are indicated on the top of each figure. See Figure 9 legend for details on scale and labels.

The presence of the *IGF-1/PI3K/mTOR* group in the membership matrix of Figure 7 (red lettering on right-hand side) is especially important in light of the growing recognition of the importance of mTOR pathway activation in epileptogenesis [18], [29], [30] and more generally in synaptic plasticity [31]–[33]. This group of genes (Figure 10) includes genes for the insulin receptor INSR and its transducer IRS1, PI3 kinase signaling components PIK3CA and PIK3R1, and AKT3, and downstream translational regulation proteins p70S6 kinase 1 (RPS6KB1), EIF4EBP2, and EIF4G2. Detailed profiles for the expression ratios of these genes are shown in Figure 11 and Figure S21A, B in File S9, showing moderate but consistent effects across several time points. On the other hand, transcriptional modulation of IGF-1 and BDNF, upstream signaling molecules important to PI3K activation in a neuronal context [34]–[36] was not significant (Figure S21C, D in File S9).

In summary, these results indicated that persistent induction of a significant number of Wnt and IGF-1/PI3K/mTOR signaling components occurs in response to hypoxic seizures, this induction occurring simultaneously with regulation of large numbers of genes involved in glutamatergic, synaptic or axonal processes.

### Hypoxic seizure responsive genes modulated by NBQX include Wnt signaling components

We next determined the subset of the HS responsive genes which were also transcriptionally regulated by NBQX treatment after hypoxia (with profile comparisons as initially sketched in Figure 1B). Because in our model NBQX treatment suppresses epileptogenesis in the wake of hypoxic seizures, we expected that this sub-setting procedure might enrich for genes more closely associated with the epileptogenic process itself. To first determine the genes differentially regulated by NBQX, two-way analysis of variance including a treatment effect (HS+NBQX versus HS) and a time effect was performed for hippocampal and cortical tissues separately (see Methods). Selection of genes on treatment effects then resulted in lists of 616 and 267 genes, for hippocampus and cortex, respectively. Intersection of these lists on a tissue-by-tissue basis (Figure 5) with the set of 1,399 HS responsive genes already analyzed above generated an “epileptogenic” set of 257 genes (Figure 5B, genes indicated in File S2), with clustered heat map as shown in Figure S22 in File S9. Most of the genes in the epileptogenic set are differentially up-regulated under HS+NBQX relative to HS alone.

**Figure 12:**
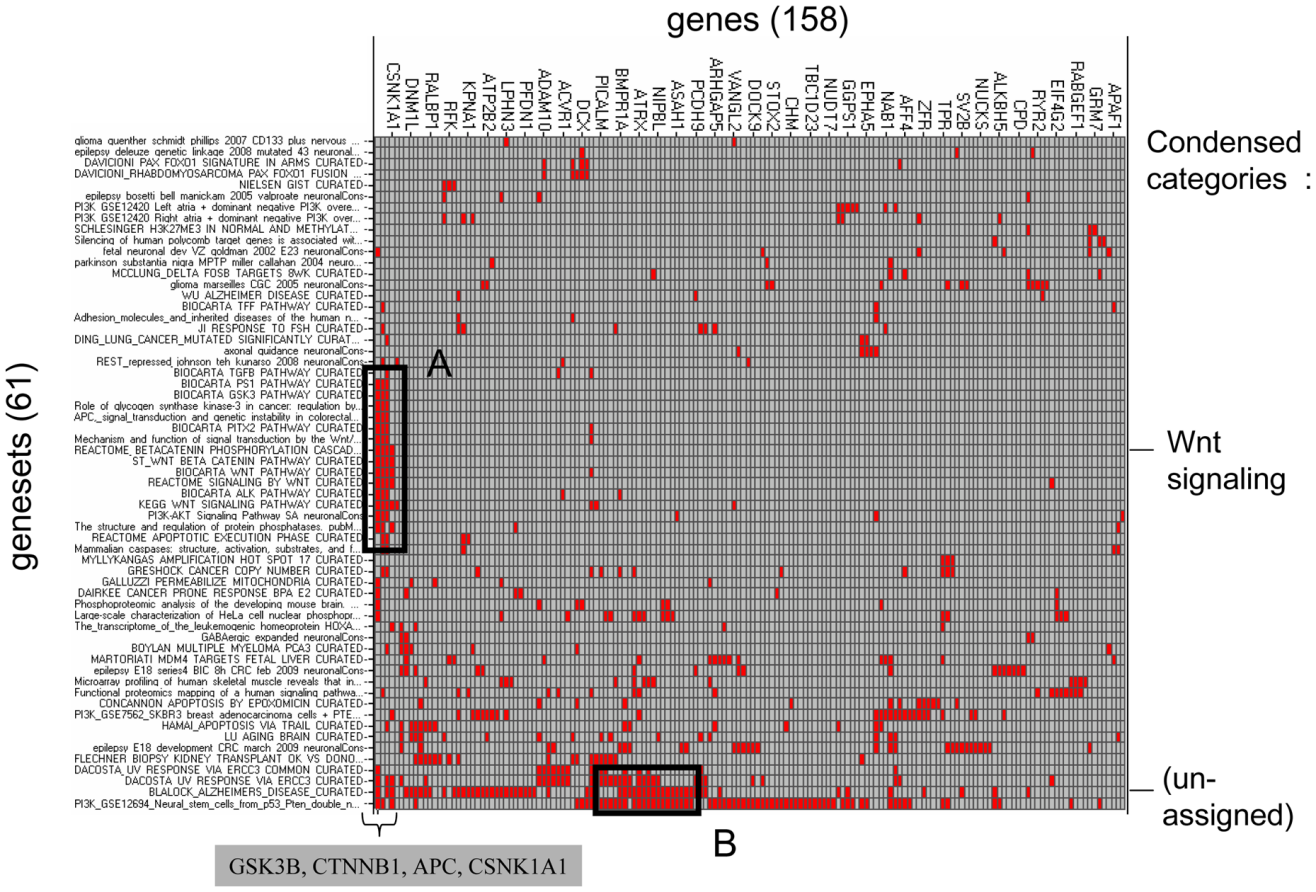
Enrichment membership matrix for the NBQX-responsive gene set. Clustered {gene set × gene} membership matrix for the 61 gene sets and the corresponding 158 genes selected by gene set enrichment analysis of the NBQX-responsive gene set. Red indicates presence of a gene (column) in given gene set (row), and gray its absence in a given gene set. Visual inspection suggested 2 functional clusters (A and B), named on the right-hand-side of the figure. In cluster A, consistent enrichment of gene sets related to Wnt signaling is due to the presence of the four genes CTNNB1, GSK3B, APC and CSNK1A1 (inset below). Cluster B did not have obvious functional categorization, and is labeled “unknown”.

Gene set enrichment analysis of the epileptogenic set was then performed with four of the gene sets collections already used for the analysis of the hypoxia-responsive profiles (see Methods). The analysis resulted in selection of 61 gene sets significantly enriched in the epileptogenic set. As had been done for the hypoxia-response analysis, clustering of the gene membership matrix for the 61 gene sets (Figure 12) was then performed to determine salient clusters of genes and gene sets. Only two prominent clusters resulted from this analysis however, and only one of these could be easily assigned a functional category (*Wnt Signaling*), due to the recurrent presence of the four Wnt signaling pathway genes beta-catenin (CTNNB1), GSK3B, APC and casein kinase 1 alpha (CSNK1A).

Inspection of individual expression profiles for the four Wnt signaling genes (Figure 9A–D, left and right-hand profiles) shows that changes in expression response to NBQX were the largest for beta-catenin (Figure 9A): thus in hippocampus at 12 h, hypoxic seizures alone induces a 2.3-fold change relative to baseline, and hypoxic seizures followed by NBQX treatment induces a 3.9-fold change in expression relative to baseline. In comparison, the relative inductions of GSK3B, APC and CSNK1A1 were muted (Figure 9C–D).

Because of the prominence of four components of the Wnt-signaling pathway in the results of the enrichment analysis, we asked whether Wnts themselves were significantly regulated in the experiments, either by hypoxic seizures alone or differentially by NBQX treatment. Of the 10 Wnt genes represented on the microarray however, only WNT5A was present in the hypoxic seizure response set (Figure S23A in File S9), and not differentially regulated by NBQX. WNT2 exhibited a statistically marginal induction in response to hypoxic seizures (Figure S23B in File S9). The other Wnts exhibited non-significant changes in expression (Figure S23C–K).

## Discussion

We investigated gene expression in the hypoxic seizure model of acquired epilepsy, a rodent model in which the progression to later life epileptic seizures has been previously described [15]. Rat neural tissues were transcriptionally profiled over the critical time interval P10–P17 and the resulting gene expression data was then analyzed with the goal of uncovering processes specific to epileptogenesis. In this analysis, we systematically applied gene set enrichment methods, an approach which emphasizes regulation of entire functional categories and pathways rather than just of individual genes.

Gene expression in the baseline developmental time course provided by the control samples was found to be consistent with known processes of neuronal maturation for the observed time interval in the rat. Thus, gene sets specifying cellular proliferation or progenitors lineages were repressed over time, while gene sets for the mature neuronal lineages were induced. The relative ranking of effects observed, with genes representing glial cells more strongly induced than those indicative of the already more mature and hence less strongly differentiating neurons, was also consistent with the known order of differentiation events. More generally, the developmental signatures taken across all genes provide a detailed view of the differentiation process ongoing in cortex and hippocampus, and can be used for baseline comparisons in future studies.

Gene expression in the response to hypoxia was then analyzed, with the central result that Wnt and of IGF-1/PI3K/mTOR signaling components are persistently induced by hypoxic seizures. Wnt signaling components were also found to be NBQX-responsive, adding support to their potential role in epileptogenesis [18]. These results are of particular interest, as both pathways are already known to be effectors in generating long-lasting changes in synaptic connectivity [24], [33], [37]–[47], and in the case of IGF-1/PI3K/mTOR signaling, in promoting epileptogenesis [48]–[51].

In the context of Wnt signaling, we saw that beta-catenin displays large transcriptional changes in response to both hypoxic seizures and to additional NBQX treatment. Beta-catenin has important roles in formation and maintenance of neuronal structures and in enabling synaptic plasticity [24]. In particular neuronal activity enhances dendritic arborization in part through beta-catenin dependent mechanisms [43]; in hippocampal neurons, depolarization drives beta-catenin from dendritic shafts into dendritic spines, where by joining cell-cell adhesion complexes it enhances the size or number of synaptic connections [44], these effects being concomitant with the release of Wnt protein at the synaptic sites [45]. Most consistent with the present findings is the observation of induction of beta-catenin itself in chronic electroconvulsive seizures in the rat hippocampus [46].

Regarding signaling along the IGF-1/PI3K/mTOR axis, directly relevant to the present work is an assessment of the role of mTOR signaling in an identical rodent model of hypoxia-induced epileptogenesis [18]. In this study, the authors established that hypoxic seizures lead to rapid and transient induction of BDNF protein expression, followed by activation of components of the PI3K/AKT/mTOR and ERK signaling pathways. Treatment of the animals with the mTOR inhibitor rapamycin following hypoxic seizures attenuated the seizure-induced molecular changes at the protein level, protected against development of sub-acute susceptibility to kainate-induced seizures, and greatly reduced the development of spontaneous seizures 5 to 6 weeks later, thereby demonstrating strong anti-epileptogenic effect. More generally mTOR signaling is dysregulated in several models of congenital or acquired epilepsy, where it displays activation above normal [30], [47]). Rapamycin has anti-epileptogenic effects in kainate or pilocarpine models of temporal lobe epilepsy, where beside directly inhibiting mTOR activation, it inhibits mossy fiber sprouting [48], neurogenesis and the long-term development of spontaneous epilepsy [49], [29]. Rapamycin also prevents epilepsy in a mouse model of the tuberous sclerosis complex [50], alterations of which in general impact neuronal morphology and function as well more directly mTOR signaling [52], [53]. These observations have led to the proposal of rapamycin as an anti-epileptogenic drug [51], [54], [55].

Other prominent enriched categories found in the hypoxic seizure response included gene sets representative of glutamatergic, synaptic and axonal processes, with in particular induction of genes for AMPA receptor subunits, and for the calcium/calmodulin-dependent kinases. These changes might reflect synaptic reorganization in the wake of hypoxia-induced seizures, perhaps under upstream control of Wnt or IGF-1/PI3K/mTOR pathway signaling. While we did not observe significant changes at the mRNA level of extracellular ligands BDNF, IGF1 or for most of the Wnts, protein levels of these ligands might still be enhanced by non-transcriptional mechanisms such as enhanced translation or rates of extracellular release.

In brief our work supports the implication of PI3K/mTOR signaling in epileptogenesis, and suggests that aspects of Wnt signaling might also be involved. For future studies of the role of these pathways, the hypoxic seizure model can be optimized and expanded in a number of ways. From a technical point of view, hypoxic seizure severity might be increased to maximize transcriptional response, which in this study was only moderate. In generating signatures, mTOR or PI3K inhibitors might be used as putative anti-epileptogenic agents, so as to directly target these potentially relevant pathways, in a research direction in harmony with ongoing clinical trials, for instance of the mTOR inhibitor everolimus [56], [57] for the treatment of refractory epilepsy. The role of Wnt signaling in epileptogenesis might also be investigated, through testing of the effect of selective Wnt pathway modulators, for instance signaling suppressors such as inhibitors of tankyrase [58], [59] or porcupine [59], and activators such as lithium [60]. Study of the effect of Wnt pathway modulators may thus eventually open a new, parallel avenue of research for the inhibition of epileptogenesis.

## Materials and Methods

### Hypoxic seizure model

The rodent model of hypoxic seizures and their consequences over time in the CNS of young rats has been previously described [13], [14], [15]. Briefly, 10 days post-natal (P10) Long-Evans hooded rat pups are subjected to 15 minutes of mild hypoxia (∼ 5% O_2_). This treatment induces immediate, acute seizures, as detected by observation of convulsive movement, or by concurrent electrophysiology using depth electrodes [17]. The acute seizures quickly subside following restoration of normoxia. However, a latent period of epileptogenesis actually follows: electrographic monitoring of the animals during a period of several weeks shows that the level of spontaneous brain activity, initially decreasing after the hypoxic insult, starts increasing again, and eventually reaches levels well above baseline controls, manifesting frequent bursting activity. 30 days after hypoxia, the animals display significantly decreased thresholds to seizures by convulsants such as kainic acid. 45 to 50 days after hypoxia, in addition to heightened induced seizure susceptibility, a large fraction of the animals (70%) also display spontaneous seizures.

All procedures were approved by and undertaken in accordance with the guidelines of the Animal Care and Use Committee at Children's Hospital (Boston, MA, U.S.A.) and the National Institutes of Health Guide for the Care and Use of Laboratory Animals. All efforts were made to minimize animal suffering and the number of animals used.

### Experimental design for gene expression profiling

The experimental design of the model for hypoxia-induced epileptogenesis is shown in Figure 1A. Cohorts of ten days old (P10) rat pups were subjected to one of three experimental conditions: 1) a control sham treatment inducing no hypoxic seizures (noHS), 2) a treatment under hypoxia inducing hypoxic seizures (HS), in which each animal underwent a 15 minutes passage at 5% O_2_, and 3) a treatment with hypoxia inducing hypoxic seizures, followed by four intra-peritoneal injections of NBQX over a 48 h period, starting at 30 minutes post-hypoxia (HS+NBQX). Control vehicle injections of DMSO were performed for the animals under the noHS and HS conditions. Time of occurrence and number of seizures for the animals undergoing hypoxia were recorded and analyzed. Times to the first seizure were found to be exponentially distributed, with mean 114 seconds. The total seizure count for the 15 minute hypoxic interval was approximately normally distributed, with mean 10.6 and standard deviation 2.9.

For each treatment condition, cohorts of 4 to 6 rat pups were killed at five sampling times spanning 1 hour to 1 week post-hypoxia (1 h, 6 h, 12 h, 48 h, 168 h post-hypoxia), for a total of 75 animals sacrificed. Whole cortex and hippocampi were extracted from the sacrificed animals and separately profiled on two Affymetrix high-throughput plate microarrays after RNA quality control, resulting in a total of 144 CEL files encapsulating the gene expression data.

### Microarray data preprocessing

The 144 CEL files, generated from scans of the individual microarrays on the two 96-well Affymetrix HT Rat Focus Arrays [61], were processed according to the MAS5 algorithm [62], resulting for each input CEL file in output of 24,249 probeset-level intensities and corresponding absent/present calls. For each processed scan, two summary statistics, consisting of the total number of present calls on the chip, and the 75^th^ percentile of present call intensities, were then computed. A quality control step was performed by inspecting for outlier scans in the space of these two statistics (no outliers were found). Additional visual inspection of a heat map of clustered profiles identified a potential tissue assignment swap between two samples, which were conservatively eliminated from further analysis. The resulting 142 quality-controlled profiles were then assembled into a single data matrix, and normalized to each other by linear rescaling of all probeset intensities so as to bring the 75^th^ percentile of present call intensities on all chips to a single common value. These processing steps, and most of the subsequent analyses discussed here, were done using the Gecko gene expression analysis platform [63]. Additional analyses were performed using the R statistical tools framework [64].

### Gene Expression Omnibus

The experimental design and the individual CEL files for the study have been deposited in the Gene Expression Omnibus [19], under data set GSE44903.

### Data standardization

When required, standardization of profiles was performed for each probeset separately, by first log2-transforming the MAS5 intensity values for that probeset, then subtracting from the result the mean and dividing by the standard deviation of the log2-transformed intensities across all experimental conditions.

### Expression ratio estimation

Expression ratios between two conditions with multiple replicates available for each condition were estimated using the *Pfold* algorithm [65], a robust Bayesian estimator which takes as input the mean intensity values for each condition, and the corresponding standard deviation of measurement of the mean intensities (estimated here from the sample standard deviations of the intensities across the replicates for each condition). The corresponding P-values were computed using an equal-variance t-test. Ratios were first computed at the probeset level, then mapped to genes; when a gene mapped to multiple probesets, with corresponding multiple ratios and P-values available, the ratio and P-value corresponding to the smallest P-value were assigned to that gene.

### Mapping of probesets to genes

Mapping of Affymetrix probesets to genes was implemented using a correspondence file derived from the Rat230_2.na27.annot.csv library file (NetAffx Annotation Release 27) available from the Affymetrix NetAffx service site [61]. The resulting mapping is many-to-many, with generally each gene mapping to multiple probesets and occasionally one probeset mapping to multiple genes. In the assignment of intensities, when a gene corresponded to multiple probesets, the largest intensity available was assigned as that gene's measured intensity. In the assignment of expression ratios, the instance with the smallest P-value was assigned as that gene's computed expression ratio, as already described above.

### Unsupervised filtering and clustering of genes for the developmental time course

After mapping of probeset intensities to genes in the gene expression data matrix, for each gene in turn a sum-of-squares statistic *S^2^* was computed (see File S8). 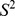 is largest for genes with many moderate deviations and/or a few large deviations from the mean, and hence detects genes with significant variation across the entire breadth of samples. In generating Figure 2, the data matrix was subsetted to the 1,000 genes out of 14,405 which had the largest values of 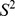. Profiles were standardized as described above, and then clustered on genes using a self-organized map.

### ANOVA to determine the hypoxic seizure response set

To generate the hypoxic seizure response set, two-way analyses of variance (ANOVAs) were separately performed on the hippocampal and cortical gene expression profiles to find microarray probe sets showing significant effects under hypoxia relative to baseline, normoxic conditions. Effects considered were treatment (levels  =  {control, hypoxia}) and time (levels  =  {1 h, 6 h, 12 h, 48 h, 168 h}), and interaction. Microarray probe sets were selected on the basis of the treatment-effect P-value, using a false-discovery rate of 0.25, following which they were mapped to gene symbols. Sets of 1,049 and 969 genes, out of the total of 14,405 represented on the microarray, were found to be modulated by hypoxic shock in hippocampus and cortex, respectively. The intersection of the two sets consisted of 621 genes, a highly significant number (P<10^−9^) as only 71±8 (se) genes would be expected for two sets of comparable size chosen at random. The (non-redundant) union of the two sets contained a total of 1,399 genes (File S2).

### Regulated KS analysis for gene set enrichment analysis of the hypoxia response

Gene sets were scored for enrichment against the entire profile of log2-ratios of hypoxia to control intensities for 14,405 genes, using an algorithmic extension of the Kolmogorov-Smirnov (KS) test which we have denoted “regulated KS analysis”. In computing enrichment, the regulated KS analysis accounts for the sign of regulation of each gene as specified in the input gene set, as well as its identity. The algorithm generates a P-value, “left” and “right” enrichments scores *C_L_* and *C_R_*, and a distributional plot (KS plot) of log2 ratio values for gene set members relative to the profile being investigated.

Briefly, consider a query gene set representing a biological pathway, with *k_u_* genes known to be positive regulatees and *k_d_* genes known to be negative regulatees of the pathway (with subscripts *u* and *d* standing for “Up” and “Down” regulation, respectively), the gene set containing a total of *k* = *k_u_* + *k_d_* genes. For a given target profile of *n* expression values (*n* = 14,405), for which enrichment of the query gene set is to be determined, the expression values are first transformed into ranks, with rank  = *1* corresponding to the largest value and rank  =  *n* to the smallest value in the profile. The empirical cumulative distribution functions (CDFs) of the ranks of the *k_u_* and *k_d_* positive and negative regulatees of the gene set are then independently estimated, and the maximum (signed) deviations *d_u_* and *d_d_* from the CDF for a uniform distribution are computed (*d_u_* and *d_d_*. are signed versions of the usual Kolmogorov-Smirnov positive-value statistic |*d|* [66]). A single test statistic combining *d_u_* and *d_d_* is then formed,
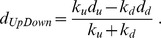



Note that as statistic, *d_UpDown_* has a number of desirable properties: it has a well-defined scale (−1 ≤ *d_UpDown_* ≤ 1), has continuous behavior as *k_d_* or *k_u_* → 0, and it weighs the contribution from each regulatee group in proportion to its membership, so that e.g. a small and noisy set of negative regulatees will not overwhelm the signal from a complementary, larger set of positive regulatees, and vice-versa. The mathematical details for computation of a P-value based on *d_UpDown_*, and of the enrichment scores *C_L_* and *C_R_*_,_ are described in File S8.

The cumulative distributions of positive and negative regulatees are graphically displayed together in a “KS plot”, where fractional rank in the sample (the regulatee group) is plotted against fractional rank in the population (all genes represented on the microarray) for each regulatee group in turn (see Figure 4C, D). Color coding of the distribution curves is red for positive and green for negative regulatees. A single set of the 95% confidence limits that obtain under the null hypothesis of random incidence of the regulates in the population is also indicated in the plot (for the total number *k = k_u_ + k_d_* of regulatees). Large enrichments correspond to widely separated positive and negative regulate distribution curves, with sharp slopes at the ends.

To more fully characterize the gene set relative to the given target profile, we also determine the subset consisting of only “leading-edge” genes [67]: for each set of regulatees separately, these are the genes that occur in the ranked lists up or down to the maximum absolute values of the KS statistics *d_u_* and *d_d_*, respectively.

We note that the integrative approach embodied in the gene set enrichment analysis was all the more useful as fold-changes for individual genes were generally moderate. The analysis focus on gene sets, rather than on a single gene at a time, enables one to pool many moderate effects, so as to gain statistical power in detection of activity at the level of entire pathways or functional categories.

### Gene set collections for gene set enrichment analysis

In order quantify which biological pathways are affected by hypoxia, we considered the 12 h profile in hippocampus as most representative, and performed gene set enrichment analysis for this profile using gene sets arising from five distinct collections. These collections included a manually curated set of neuronally relevant groups of genes, already discussed in connection with the developmental profiles analyzed above (*n* = 372 gene sets), a collection internally generated from parsing of PubMed [68] articles abstracts (*n* = 802), and three publicly available collections [69], namely collections derived by manual curation (*n* = 2,457), from the Gene Ontology [70] (*n* = 1,454) or from the Connectivity Map [71] (*n* = 1,309), for a grand total of 6,394 gene sets.

Each gene set in each collection was scored against the entire profile of log2-ratios of hypoxia to control intensities for the 14,405 genes represented on the microarray using the regulated KS analysis method presented above. For each of the five collections considered, we selected statistically significant gene sets by requiring a false-discovery rate of less than 0.25 and an enrichment score of at least 2. Collecting results, we found a total of 37 gene sets scoring significantly against the 12 h hippocampal profile under the regulated KS analysis (Table 1, File S3), for a total non-redundant content of 1,041 genes. To further organize this result, for each of the 37 significant gene sets we retained only its leading-edge genes, and also required that each gene originate from at least two gene sets before incorporating it into a final list, this procedure insuring that genes considered were individually and significantly modulated in the relevant context defined by their gene sets. The procedure resulted in a set of 261 genes, and a corresponding 37×261 gene membership matrix (gene sets versus selected genes) (File S4).

To generate an even more succinct list of genes, we further required that the 261 pathway-relevant genes, arising from the enriched gene sets as discussed above, also individually show significant effects of hypoxia relative to control, by belonging to the ANOVA-selected list of 1,399 genes. This resulted in a list of 76 significantly modulated, pathway-relevant genes (list in File S6, expression ratios in File S2) which summarizes the gene content of the enrichment analyses.

Finally, a gene set enrichment analysis focused on Wnt signaling alone was performed, using a small collection of Wnt-responsive gene sets [27] joined to a group of manually curated Wnt-relevant gene sets.

### ANOVA to determine the epileptogenic set

To determine the genes differentially regulated by NBQX in a hypoxia background, two-way analyses of variance including a treatment effect (levels  =  {hypoxia+NBQX, hypoxia+vehicle}) and a time effect (levels  =  {1 h, 6 h, 12 h, 48 h, 168 h}), were performed for hippocampal and cortical tissues separately. Selection based on treatment effects was then done as follows, depending on tissue; 1) hippocampus: a false-discovery-rate (FDR) threshold was used, with FDR  =  0.25 (P = 3.35 ×10^−3^), resulting in selection of 616 genes, of which 235 overlapped with the set of 1,051 hypoxic seizure responsive genes in hippocampus; 2) cortex: a P-value threshold of P = 10^−2^ (corresponding to the very lax threshold FDR ∼ 1) resulted in a selection of 257 genes, 54 of which overlapped with the hypoxic seizure responsive genes in cortex. The union of the two resulting sets contained 257 genes, defining the “epileptogenic” set (indicated in File S2).

Note when acting to inhibit epileptogenesis, NBQX may function by either repressing pro-epileptogenic genes, or by enhancing the action of anti-epileptogenic protective genes (Figure 1B): thus both NBQX-induced and NBQX-repressed genes are included in the “epileptogenic” gene set.

### Enrichment analysis for the epileptogenic set

Four of the five gene set collections (excluding the Connectivity Map collection) already used for enrichment analysis of the hypoxia response set (see above) were used for enrichment analysis of the epileptogenic set. The nested selection criteria defining the epileptogenic set made assignment of a single representative gene expression profile ambiguous, and hence the Fisher exact test rather than the regulated KS analysis was used to establish whether overlap between a given query gene set and the target epileptogenic set of 257 genes was significant. A background of *n* = 14,405 genes was assumed throughout. P-value thresholds for significant enrichment were set at 0.01. The analysis resulted in selection of 61 gene sets significantly enriched in the epileptogenic set, these gene sets altogether containing a (non-redundant) set union of 158 genes. Unsupervised clustering analysis of the 61×158 gene membership matrix for the 61 gene sets was then performed to determine salient groups of associated genes and gene sets.

An independent enrichment analysis of the epileptogenic set was also conducted using the NextBio analysis platform [72], in which the NextBio curated gene set collection was queried. The most prominent enrichment detected (34 gene overlap, P = 5.3×10^−17^) was for a set of genes induced in the rat hippocampal CA3 region in response to a spatial learning task [73], a result consistent with epileptogenesis inducing persistent modifications in neuronal connectivity.

## Supporting Information

Table S1 **Enrichment analysis results for seven Wnt-relevant gene sets.** This small gene set collection includes sets of transcriptional targets of the activated pathway, as well as two sets of genes in the signaling pathway itself. Only the WNT3A target gene set [28] showed significant enrichment (see Figure S20 in File S9 for KS plot). Column keys: ku  =  number of positive regulates in gene set; kd  =  number of negative regulatees in gene set; P-value  =  P-value from regulated KS test; FDR  =  false-discovery rate; the enrichment factors cLeft and cRight refer to slopes of the distribution functions in the KS plots (see Methods).
(XLS)

Table S2 **List of the 76 significantly modulated, pathway-relevant genes for the hypoxic seizure response.** This set of genes is defined by the intersection of the HS response set of 1,399 genes determined by ANOVA, and the set of 261 leading-edge genes determined by the gene set enrichment analysis of the 12 h hippocampal response profile. The list is also provided in File S6, and expression ratios in File S2.
(XLS)

File S1 **Experimental design for the hypoxic seizure model, including physiological response information.** This file contains the experimental design information for the samples matching the 142 Affymetrix scans which passed quality-control. Physiological response to hypoxia, including seizure count, is also shown. The individual CEL files for the study have also been deposited in the Gene Expression Omnibus [19], data set: GS44903).
(XLS)

File S2 **Expression ratios and P-values for the 1,399 genes in the hypoxic seizure (HS) response set.** the subset of 257 NBQX-responsive genes is indicated in the table.
(XLS)

File S3 **Gene sets enriched in the response to hypoxic seizures.** The table displays the 37 gene sets out of an initial set of 6,394 which scored significant enrichment against the 12 hour hippocampal response to hypoxic seizures (the contents are identical to those of Table 1).
(XLS)

File S4 **Membership matrix for the enriched gene sets in the hypoxic seizure response.** The 261 * 37 membership matrix for the 261 leading-edge genes in the 37 gene sets which scored significantly against the 12 h differential response to hypoxic seizures in hippocampus, with additional filter requiring that each gene appear in at least two gene sets, is displayed below (gray columns).
(XLS)

File S5 **Table of expression ratios and P-values for the 261 genes in the membership matrix for the hypoxic seizure response.**
(XLS)

File S6 **List of the 76 significantly modulated, pathway-relevant genes for the hypoxic seizure response.** This set of genes is defined by the intersection of the HS response set of 1,399 genes determined by ANOVA, and the set of 261 leading-edge genes determined by the gene set enrichment analysis of the 12 h hippocampal response profile. The list is displayed in Table S2, and the genes are also indicated in the expression ratio spreadsheet, File S2.
(XLS)

File S7 **Supplementary Results.** Description of gene expression for glutamatergic and GABAergic receptors in the developmental time course, and of additional gene sets enriched in the hypoxic seizure response.
(DOC)

File S8 **Supplementary Methods.** Mathematical details for the regulated KS analysis.
(DOC)

File S9 **Supporting Figures.** Contains all Supporting Figures, S1–S23. **Figure S1. Mitosis gene set under baseline developmental conditions.** A. Log2-ratios of gene expression relative to the 1 h time point, in each tissue separately, are displayed as a heat map, with sampling times post-P10 indicated in hours. Colors saturate for log2-ratio  = ±1. Strong repression of many genes is observed, including for the genes coding for the chromosomal passenger proteins aurora kinase B (AURKB) and surviving (BIRC5), and for several centromeric proteins. B. KS plot showing the distribution of log2-ratios at t = 1 week for both tissues. P-value and enrichment are reported for hippocampus only. Hipp =  hippocampus, cx =  cortex. The arrows point to some of the genes referred to in the main text. **Figure S2. Gene set containing sonic hedgehog (SHH) target genes under baseline developmental conditions.** A. Strong repression of most genes in the gene set is observed by t = 1 week. B. Detailed intensity profiles for CXCR4 and TOP2A. C. KS plot showing the distribution of the log2-ratios at t = 1 week for both tissues. See Figure S1 for key to figure color and scale details. **Figure S3. Neural stem cell markers under baseline developmental conditions.** A. Repression of several genes in the gene set is observed by t = 1 week, including CXCR4, CD24 and nestin (NES). B. Detailed intensity profile for CD24. c) KS plot showing the distribution of the log2-ratios at t =  week for both tissues. See Figure S1 for key to figure color and scale details. **Figure S4. Neuronal progenitor markers under baseline developmental conditions.** A. Repression of several genes in the gene set is observed by t = 1 week, including doublecortin (DCX) and OLIG2. B. KS plot showing the distribution of the log2-ratios at t = 1 week for both tissues. See Figure S1 for key to figure color and scale details. **Figure S5. A gene set containing multiple neuronal markers under baseline developmental conditions.** a) Heat map of gene profiles. b) KS plot showing the distribution of the log2-ratios at t = 1 week for both tissues (P-value and enrichment here displayed for cortex). See Figure S1 for key to figure color and scale details. **Figure S6. A gene set containing an empirical collection of neuronal markers under baseline developmental conditions.** The markers were obtained from the Allen Brain Atlas [21]. A. Heat map of gene profiles. B. KS plot showing the distribution of the log2-ratios at t = 1 week for both tissues. See Figure S1 for key to figure color and scale details. **Figure S7. A comprehensive collection of synaptic markers under the baseline developmental conditions.** A. Heat map of gene profiles, showing the 22 genes most induced in cortex. Gene descriptions are given on the right, with functional categories color-coded as specified in the legend. B. Detailed intensity profiles for the chloride transporter KCC2. C. KS plot showing the distribution of the log2-ratios at t = 1 week for both tissues. See Figure S1 for key to figure color and scale details. **Figure S8. Astrocytic markers under the baseline developmental conditions.** A. Heat map of gene profiles, showing induction of several genes, including connexin 30 (GJB6), glutamine synthesase (GLUL) and GLT-1 (SLC1A2). B. Detailed intensity profiles for GFAP, AQP9 and connexin 30. c) KS plot showing the distribution of the log2-ratios at t = 1 week for both tissues. See Figure S1 for key to figure color and scale details. **Figure S9. A gene set containing an empirical collection of oligodendrocytic markers under the baseline developmental conditions.** The markers were obtained from the Allen Brain Atlas [21]. A. Heat map of gene profiles, showing very strong induction of many genes, including genes for the myelinating proteins already indicated in Figure 4 (left-hand arrows). A. KS plot showing the distribution of the log2-ratios at t = 1 week for both tissues, with very marked leading edges. See Figure S1 for key to figure color and scale details. **Figure S10. NMDA receptor components under the baseline developmental conditions.** A. Heat map of gene profiles, showing induction of NR1 and NR2C and repression of NR3A and NR2D. B. Detailed log2-ratio profiles for NR1, NR2C, NR3A and NR2D in hippocampus and C, in cortex. See Figure S1 for key to figure color and scale details. **Figure S11. AMPA receptor components under the baseline developmental conditions.** A. Heat map of gene profiles, showing non-significant changes in hippocampus, and repression of GLUR1 and GLUR2 in cortex. B. Detailed log2-ratio profiles for GLUR2 in hippocampus and C, for GLUR1 and GLUR2 in cortex. See Figure S1 for key to figure color and scale details. **Figure S12. GABA receptor components and glutamate decarboxylase 1 and 2 under the baseline developmental conditions.** A. Heat map of gene profiles, showing generally non-significant changes. B. Detailed log2-ratio profiles for GABA_A_-δ (GABRD), GABA_A_-β1 (GABRB1) and KCC2 in hippocampus and C, in cortex. See Figure S1 for key to figure color and scale details. **Figure S13. Heat map of the differential response to hypoxic seizures for the genes in the** ***In vitro response (BIC or 4AP)*** **group.** The group is defined by one of the clusters of enriched gene sets in the membership matrix of Figure 7. Colors represent log2-ratios of intensities of hypoxic seizure samples to that of time-matched controls (red  =  positive, green  =  negative values, colors saturate at ±1). Arrows point to genes referred to in the main text. **Figure S14. Heat map of the differential response to hypoxic seizures for the genes in the** ***Hypoxia response*** **group.** The group is defined by one of the clusters of enriched gene sets in the membership matrix of Figure 7. See Figure S13 for key to figure color and scale details. **Figure S15. Heat map of the differential response to hypoxic seizures for the genes in the** ***Glutamatergic/NMDA receptor I*** **group.** The group is defined by one of the clusters of enriched gene sets in the membership matrix of Figure 7. See Figure S13 for key to figure color and scale details. **Figure S16. Heat map of the differential response to hypoxic seizures for the genes in the** ***Glutamatergic/NMDA receptor II*** **group.** The group is defined by one of the clusters of enriched gene sets in the membership matrix of Figure 7. See Figure S13 for key to figure color and scale details. **Figure S17. Heat map of the differential response to hypoxic seizures for the genes in the** ***Synaptic*** **group.** The group is defined by one of the clusters of enriched gene sets in the membership matrix of Figure 7. See Figure S13 for key to figure color and scale details. **Figure S18. Heat map of the differential response to hypoxic seizures for the genes in the** ***Axonal Guidance*** **group.** The group is defined by one of the clusters of enriched gene sets in the membership matrix of Figure 7. See Figure S13 for key to figure color and scale details. **Figure S19. Heat map of the differential response to hypoxic seizures for the genes in the** ***Autism associated*** **group.** The group is defined by one of the clusters of enriched gene sets in the membership matrix of Figure 7. See Figure S13 for key to figure color and scale details. **Figure S20. WNT3A target genes are enriched in the hypoxic seizure response profile.** Enrichment analysis with the WNT3A target gene set [28] was performed against the 12 hour profile of transcriptional response to hypoxic seizures in hippocampus. A. KS plot showing enrichment profile. B. Table of the corresponding 15 leading-edge genes. Ratios are of gene expression intensities in hypoxic seizure sample to corresponding control. **Figure S21. Expression ratios as a function of time for selected IGF-1/PI3K/mTOR pathway genes.** The gene name is indicated at the top of each figure. Time at the bottom refers to the duration following hypoxic seizures; the left-hand panels (labeled “hyp”) display ratios of hypoxic seizure response to control; the right-hand panels (labeled “hyp + NBQX”) displays ratios of response to combined hypoxic seizures and NBQX treatment, to control. The expression ratio R = 1 (no fold change) is indicated by the black horizontal lines. A. IRS1 displays ∼ 1.6 fold induction at 12 h. B. AKT3 is very moderately but significantly induced over the time course. C and D. IGF1 and BDNF are not significantly induced. **Figure S22. Heat map of differential expression for the NBQX-responsive genes.** The profiles of the 257 genes, each of which is differentially regulated by both hypoxic seizures (HS) relative to control, and by HS+NBQX relative to HS, are shown (these genes form a proper subset of the 1,399 HS-responsive genes displayed in Figure 6). Log2 ratios of gene expression between time-matched samples are shown for the ratio combinations indicated on top. Colors saturate for log2-ratio  = ±1. **Figure S23. Expression ratios as a function of time for 11 Wnt genes represented on the microarray.** The gene name is indicated at the top of each figure. Time at the bottom refers to the duration following hypoxic seizures; the left-hand panels (labeled “hyp”) display ratios of hypoxic seizure response to control; the right-hand panels (labeled “hyp + NBQX”) displays ratios of response to combined hypoxic seizures and NBQX treatment, to control. The expression ratio R = 1 (no fold change) is indicated by the black horizontal lines. Only WNT5A and WNT2 are significantly induced.
(PDF)
