# Supplementary material for: Gene Expression Profiling of a Hypoxic Seizure Model of Epilepsy Suggests a Role for mTOR and Wnt Signaling in Epileptogenesis
Source: PLoS One. 2013 Sep 27;8(9):e74428. doi: 10.1371/journal.pone.0074428 (PMC3785482; doi:10.1371/journal.pone.0074428)
Supplement: File S7 — Supplementary Results. Description of gene expression for glutamatergic and GABAergic receptors in the developmental time course, and of additional gene sets enriched in the hypoxic seizure response. (DOC) [file pone.0074428.s009.doc]

Supplementary Descriptions of Results for:

Gene expression profiling of a hypoxic seizure model of epilepsy suggests a role for mTOR and Wnt signaling in epileptogenesis

Joachim Theilhaber, Sanjay N. Rakhade, Judy Sudhalter, Nayantara Kothari, Peter Klein, Jack Pollard, Frances E. Jensen.

## The developmental time course for post P10 neuronal tissues is consistent with known neuronal maturation processes

Beyond the study of neuronal lineages as a whole, it was of some interest to examine in more detail temporal expression of genes for excitatory and inhibitory post-synaptic receptors, as receptor maturation plays an important role in perinatal seizures [1]. Thus, examination of gene expression for the NMDA receptor components (Figure S10), revealed moderate induction of subunits NR1 and NR2C (genes GRIN1, GRIN2C, respectively), alongside repression of subunit NR2D (GRIN2D) and of the Mg2+-independent subunit NR3A (GRIN3A)[2-4], over 1 week’s time, and with concordant regulation in hippocampus and cortex. On the other hand, changes in gene expression for the AMPA receptor components GLUR1 through GLUR4 (Figure S11) were not significant in the hippocampus, and in cortex only significant for down-regulation of subunit GLUR1. Inspection of gene expression for the inhibitory ionotropic GABA receptor (Figure S12) indicated that most subunit genes were not significantly changed over time, with the exception of the delta subunit GABA_A_-δ (GABRD), which was induced in both hippocampus and cortex. Finally note that gene KCC2 (SLC12A5), coding for the chloride pump essential for inhibitory action of the ionotropic GABA receptor, undergoes only moderate induction in cortex or hippocampus over the given time period (Figure S12B, C and Figure S7B).

## The hypoxic seizure response gene set is enriched in Wnt and mTOR pathway components

More biological content for the *In vitro response* *(BIC or 4AP)* group of genes can be inferred by browsing among profiles of the genes most induced in hippocampus at 12h (heat map, Figure S13). We find for instance i) the semaphorin genes SEMA6A and SEMA3A, coding for activating and inhibitory ligands of Plexin-A4 respectively, and also involved in angiogenic signaling [5], ii) the genes SHANK2 and HOMER1, coding for important post-synaptic scaffolding proteins, iii) genes WNT2, DVL3 and SFRP2, coding for ligand, and transducing and inhibitory members, respectively, of the Wnt signaling pathway, iv) genes GABRA4 and mGLUR2 (GRM2), for receptor components of GABAergic and metabotropic glutamatergic synaptic signaling, respectively, and v) GLAST1 (SLC1A3), coding for a glutamate transporter abundant in astrocytes.

Enrichment of the *Hypoxia response* gene set (Figure S14), with induction of hypoxia response target genes VEGFA and CREBBP, is consistent with the occurrence of hypoxia, and can be considered another positive control for the experiment.

The small group of *Axonal guidance* genes (Figure S18) includes members such as the SEMA3A and SEMA6A, already noted above in connection with *In vitro response* *(BIC or 4AP)*. The small *Autism associated* group (Figure S19) includes again neuroligin-3 (NLGN3)[6, 7] and GABRA4, the latter also noted in connection with *In vitro response* *(BIC or 4AP)* and observed in gene expression profiling of acute models of acquired epilepsy[8].

**References**

1. Jensen FE (2002). The role of glutamate receptor maturation in perinatal seizures and brain injury. *Int J Dev Neurosci.* **20**:339-47
2. Zhou C, Jensen FE, Sucher NJ (2009). Altered development of glutamatergic synapses in layer V pyramidal neurons in NR3A knockout mice. *Mol Cell Neurosci.* **42**:419-26.
3. Pilli J, Kumar SS (2012). Triheteromeric N-methyl-D-aspartate receptors differentiate synaptic inputs onto pyramidal neurons in somatosensory cortex: involvement of the GluN3A subunit. *Neuroscience* **222**:75-88.
4. Kumar SS, Huguenard JR. (2003). Pathway-specific differences in subunit composition of synaptic NMDA receptors on pyramidal neurons in neocortex. *J Neurosci.* **23**:10074-83.
5. Kigel B, Rabinowicz N, Varshavsky A, Kessler O, Neufeld G (2011). Plexin-A4 promotes tumor progression and tumor angiogenesis by enhancement of VEGF and bFGF signaling. *Blood* **118**:4285-96.
6. Tabuchi K, Blundell J, Etherton MR, Hammer RE, Liu X, Powell CM, Südhof TC (2007). A neuroligin-3 mutation implicated in autism increases inhibitory synaptic transmission in mice. *Science* **318**:71-6
7. Varoqueaux F, Aramuni G, Rawson RL, Mohrmann R, Missler M, Gottmann K, Zhang W, Südhof TC, Brose N (2006). Neuroligins determine synapse maturation and function. *Neuron* **51**:741-54.
8. Roberts DS, Hu Y, Lund IV, Brooks-Kayal AR, Russek SJ (2006). Brain-derived neurotrophic factor (BDNF)-induced synthesis of early growth response factor 3 (Egr3) controls the levels of type A GABA receptor alpha 4 subunits in hippocampal neurons. *J Biol Chem.* **281**: 29431-5.
