# Supplementary material for: Gene Expression Profiling of a Hypoxic Seizure Model of Epilepsy Suggests a Role for mTOR and Wnt Signaling in Epileptogenesis
Source: PLoS One. 2013 Sep 27;8(9):e74428. doi: 10.1371/journal.pone.0074428 (PMC3785482; doi:10.1371/journal.pone.0074428)
Supplement: File S8 — Supplementary Methods. Mathematical details for the regulated KS analysis. (DOC) [file pone.0074428.s010.doc]

Supplementary Methods for:

Gene expression profiling of a hypoxic seizure model of epilepsy suggests a role for mTOR and Wnt signaling in epileptogenesis

Joachim Theilhaber, Sanjay N. Rakhade, Judy Sudhalter, Nayantara Kothari, Peter Klein, Jack Pollard, Frances E. Jensen.

**Regulated KS analysis for gene set enrichment analysis of the hypoxia response**

Gene sets were scored for enrichment against the entire profile of log2-ratios of hypoxia to control intensities for 14,405 genes, using an algorithmic extension of the Kolmogorov-Smirnov (KS) test which we have denoted “regulated KS analysis”. In computing enrichment, the regulated KS analysis accounts for the sign of regulation of each gene as specified in the input gene set, as well as its identity.

Briefly, consider a query gene set representing a biological pathway, with *k_u_* genes known to be positive regulatees and *k_d_* genes known to be negative regulatees of the pathway (with subscripts *u* and *d* standing for “Up” and “Down” regulation, respectively), the gene set containing a total of *k* = *k_u_* + *k_d_* genes. For a given target profile of *n* expression values (*n* = 14,405), for which enrichment of the query gene set is to be determined, the expression values are first transformed into ranks, with rank = *1* corresponding to the largest value and rank = *n* to the smallest value in the profile. The empirical cumulative distribution functions (CDFs) of the ranks of the *k_u_* and *k_d_* positive and negative regulatees of the gene set are then independently estimated, and the maximum (signed) deviations *d_u_* and *d_d_* from the CDF for a uniform distribution are computed (*d_u_* and *d_d_*. are signed versions of the usual Kolmogorov-Smirnov positive-value statistic |*d|* [1]. A single test statistic combining *d_u_* and *d_d_* is then formed,

$$d_{UpDown}=\frac{k_{u}d_{u}-k_{d}d_{d}}{k_{u}+k_{d}}\text{ }.$$

Note that as statistic, *d_UpDown_* has a number of desirable properties: it has a well-defined scale (-1 ≤ *d_UpDown_* ≤ 1), has continuous behavior as *k_d_* or *k_u_* → 0, and it weighs the contribution from each regulatee group in proportion to its membership, so that e.g. a small and noisy set of negative regulatees will not overwhelm the signal from a complementary, larger set of positive regulatees, and vice-versa.

Because the sampling distribution of *d_UpDown_* under the null hypothesis of uniform sample distributions is not known analytically (unless *k_d_* or *k_u_* = 0, in which case it is just given by the analytic KS distribution function, as given below), we generate it on-the-fly by computing *nr* = 100,000 random samples

$$d^{*}=\frac{k_{u}^{1/2}\xi_{u}-k_{d}^{1/2}\xi_{d}}{k_{u}+k_{d}}\text{ },$$

where ξ_u_ and ξ_d_ are independent random samples generated from the analytic KS distribution function[1]

$$P(\xi\geq\xi_{0})=2\sum_{i=1}^{\infty} (-)^{i+1}\exp(-2i^{2}\xi_{0}^{2})\text{ }.$$

The empirical Prob*(d* > d_UpDown_)* is then estimated (cumulatively, on the fly), and this is used to compute a two-sided P-value *P_UpDown_* against the null hypothesis that the regulatees have random incidence in the sorted expression profile.

Alongside the P-value *P_UpDown_*, an activation status *a_UpDown_* is assigned to the entire pathway represented by the gene set through the rule

$$a_{UpDown}=\mathrm{sgn}\left(d_{UpDown}\right)\text{ }.$$

To quantify the degree of enrichment and not just its significance, in addition to the statistic *d_UpDown_*, “left” and “right” enrichments scores *C_L_* and *C_R_* are computed from robust approximations of the slopes at the origin of the CDFs for positive and negative regulatees, respectively. Briefly, for a given quantile *q,* the enrichment *C(q)* of the CDF is defined by

$$C(q)=\frac{q}{x_{q}}\text{ },$$

where *x_q_* is the corresponding quantile in the population. For computation of *C_L_* and *C_R_* the first quartile is used here, with *q* = 0.25.

The cumulative distributions of positive and negative regulatees are graphically displayed together in a “KS plot”, where fractional rank in the sample (the regulatee group) is plotted against fractional rank in the population (all genes represented on the microarray) for each regulatee group in turn (see for instance Figure S20A). Color coding of the distribution curves is red for positive and green for negative regulatees. The 95% confidence limits that obtain under the null hypothesis of random incidence of the regulatees in the population is also indicated in the plot (for the total number *k = k_u_ + k_d_* of regulatees). Large enrichments correspond to widely separated positive and negative regulatee distribution curves, with sharp slopes at the ends.

To more fully characterize the gene set relative to the given target profile, we also determine the subset consisting of only “leading-edge” genes[2]: for each set of regulatees separately, these are the genes that occur in the ranked lists up or down to the maximum absolute values of the KS statistics *d_u_* and *d_d_*, respectively.

We note that the approach embodied in the gene set enrichment analysis was all the more useful in this study as fold-changes for individual genes were generally moderate. The algorithmic focus on gene sets, rather than on a single gene at a time, enables one to pool many moderate effects, so as to gain statistical power in detection of activity at the level of entire pathways or functional categories.

**Unsupervised filtering and clustering of genes for the developmental time course**

After mapping of probeset intensities to genes in the gene expression data matrix, for each gene in turn a sum-of-squares statistic was computed,

$$S^{2}=\sum_{i=1}^{m} \text{Sym}^{2}\left(R_{i}\right)\text{ },$$

where *m* = 142 is the number of samples in the gene expression data matrix, and *R_i_* a robust estimate of the ratio of intensity for the *i*-th sample to the mean intensity,

where *x_i_, i = 1, . . . , m*, denote individual intensities for the given gene, *σ_i_, i = 1, . . ., m,* the corresponding estimated standard deviations of the intensity measurements (Theilhaber, 2001[3]), and $\bar{x}$denotes the mean intensity across all samples. The function Sym(*R*), defined by

$$\text{Sym}(R)=\left\{\begin{matrix} (R^{2}-1)^{1/2},\text{ }R\text{ } \geq1, \\ -(1/R^{2}-1)^{1/2},\text{ }R \text{ }<1, \end{matrix}\right.$$

is a log-like function with property Sym(*R*) = - Sym(*1/R*) which provides equal emphasis on ratios *R* >> *1* and *R* << *1.* The sum-of-squares statistic $S^{2}$ is approximately proportional to a $\chi^{2}$- distributed variable (with prefactor and degrees of freedom determined empirically). $S^{2}$ is largest for genes with many moderate deviations and/or a few large deviations from the mean, and hence detects genes with significant variation across the entire breadth of samples. In generating Figure 2, the data matrix was sub-setted to the 1,000 genes out of 14,405 which had the largest values of $S^{2}$. Profiles were standardized and then clustered on genes using a self-organized map.

**References**

1. Keeping ES (1995). *Introduction to Statistical Inference* (Dover, New York) p. 256.
2. Subramanian A, Tamayo P, Mootha VK, Mukherjee S, Ebert BL, Gillette MA, Paulovich A, Pomeroy SL, Golub TR, Lander ES, Mesirov JP. (2005). Gene set enrichment analysis: a knowledge-based approach for interpreting genome-wide expression profiles. *Proc Natl Acad Sci* *U S A.* **102** : 15545-50.
3. Theilhaber J, Bushnell S, Jackson A, Fuchs R. 2001. Bayesian estimation of fold-changes in the analysis of gene expression: the PFOLD algorithm. *J Comput Biol.* **8**: 585-614.
