# Supplementary material for: Gene Expression Profiling of a Hypoxic Seizure Model of Epilepsy Suggests a Role for mTOR and Wnt Signaling in Epileptogenesis
Source: PLoS One. 2013 Sep 27;8(9):e74428. doi: 10.1371/journal.pone.0074428 (PMC3785482; doi:10.1371/journal.pone.0074428)
Supplement: File S9 — Supporting Figures. Contains all Supporting Figures, S1–S23. Figure S1. Mitosis gene set under baseline developmental conditions. A. Log2-ratios of gene expression relative to the 1 h time point, in each tissue separately, are displayed as a heat map, with sampling times post-P10 indicated in hours. Colors saturate for log2-ratio = ±1. Strong repression of many genes is observed, including for the genes coding for the chromosomal passenger proteins aurora kinase B (AURKB) and surviving (BIRC5), and for several centromeric proteins. B. KS plot showing the distribution of log2-ratios at t = 1 week for both tissues. P-value and enrichment are reported for hippocampus only. Hipp = hippocampus, cx = cortex. The arrows point to some of the genes referred to in the main text. Figure S2. Gene set containing sonic hedgehog (SHH) target genes under baseline developmental conditions. A. Strong repression of most genes in the gene set is observed by t = 1 week. B. Detailed intensity profiles for CXCR4 and TOP2A. C. KS plot showing the distribution of the log2-ratios at t = 1 week for both tissues. See Figure S1 for key to figure color and scale details. Figure S3. Neural stem cell markers under baseline developmental conditions. A. Repression of several genes in the gene set is observed by t = 1 week, including CXCR4, CD24 and nestin (NES). B. Detailed intensity profile for CD24. c) KS plot showing the distribution of the log2-ratios at t = week for both tissues. See Figure S1 for key to figure color and scale details. Figure S4. Neuronal progenitor markers under baseline developmental conditions. A. Repression of several genes in the gene set is observed by t = 1 week, including doublecortin (DCX) and OLIG2. B. KS plot showing the distribution of the log2-ratios at t = 1 week for both tissues. See Figure S1 for key to figure color and scale details. Figure S5. A gene set containing multiple neuronal markers under baseline developmental conditions. a) Heat map of gene profiles. [file pone.0074428.s011.pdf]

Supporting Figures for:

Gene expression profiling of a hypoxic seizure model of epilepsy suggests a role for mTOR and Wnt signaling in epileptogenesis

Joachim Theilhaber, Sanjay N. Rakhade, Judy Sudhalter, Nayantara Kothari, Peter Klein, Jack Pollard, Frances E. Jensen.

Supporting Figures Legends

(references refer to the main text)

Figure S1 Mitosis gene set under baseline developmental conditions. A. Log2-ratios of gene expression relative to the 1h time point, in each tissue separately, are displayed as a heat map, with sampling times post-P10 indicated in hours. Colors saturate for log2-ratio = ±1. Strong repression of many genes is observed, including for the genes coding for the chromosomal passenger proteins aurora kinase B (AURKB) and surviving (BIRC5), and for several centromeric proteins. B. KS plot showing the distribution of log2-ratios at t = 1 week for both tissues. P-value and enrichment are reported for hippocampus only. Hipp = hippocampus, cx = cortex. The arrows point to some of the genes referred to in the main text.

Figure S2 Gene set containing sonic hedgehog (SHH) target genes under baseline developmental conditions. A. Strong repression of most genes in the gene set is observed by t = 1 week. B. Detailed intensity profiles for CXCR4 and TOP2A. C. KS plot showing the distribution of the log2-ratios at t = 1 week for both tissues. See Figure S1 for key to figure color and scale details.

Figure S3 Neural stem cell markers under baseline developmental conditions. A. Repression of several genes in the gene set is observed by t = 1 week, including CXCR4, CD24 and nestin (NES). B. Detailed intensity profile for CD24. c) KS plot showing the distribution of the log2-ratios at t = 1 week for both tissues. See Figure S1 for key to figure color and scale details.

Figure S4 Neuronal progenitor markers under baseline developmental conditions. A. Repression of several genes in the gene set is observed by t = 1 week, including doublecortin (DCX) and OLIG2. B. KS plot showing the distribution of the log2-ratios at t = 1 week for both tissues. See Figure S1 for key to figure color and scale details.

Figure S5 A gene set containing multiple neuronal markers under baseline developmental conditions. a) Heat map of gene profiles. b) KS plot showing the distribution of the log2-ratios at t = 1 week for both tissues (P-value and enrichment here displayed for cortex). See Figure S1 for key to figure color and scale details.

Figure S6 A gene set containing an empirical collection of neuronal markers under baseline developmental conditions. The markers were obtained from the Allen Brain Atlas [20]. A. Heat map of gene profiles. B. KS plot showing the distribution of the log2-ratios at t = 1 week for both tissues. See Figure S1 for key to figure color and scale details.

Figure S7 A comprehensive collection of synaptic markers under the baseline developmental conditions. A. Heat map of gene profiles, showing the 22 genes most induced in cortex. Gene descriptions are given on the right, with functional categories color-coded as specified in the legend. B. Detailed intensity profiles for the chloride transporter KCC2. C. KS plot showing the distribution of the log2-ratios at t = 1 week for both tissues. See Figure S1 for key to figure color and scale details.

Figure S8 Astrocytic markers under the baseline developmental conditions. A. Heat map of gene profiles, showing induction of several genes, including connexin 30 (GJB6), glutamine synthesase (GLUL) and GLT-1 (SLC1A2). B. Detailed intensity profiles for GFAP, AQP9 and connexin 30. c) KS plot showing the distribution of the log2-ratios at t = 1 week for both tissues. See Figure S1 for key to figure color and scale details.

Figure S9 A gene set containing an empirical collection of oligodendrocytic markers under the baseline developmental conditions. The markers were obtained from the Allen Brain Atlas [20]. A. Heat map of gene profiles, showing very strong induction of many genes, including genes for the myelinating proteins already indicated in Figure 4 (left-hand arrows). A. KS plot showing the distribution of the log2-ratios at t = 1 week for both tissues, with very marked leading edges. See Figure S1 for key to figure color and scale details.

Figure S10 NMDA receptor components under the baseline developmental conditions. A. Heat map of gene profiles, showing induction of NR1 and NR2C and repression of NR3A and NR2D. B. Detailed log2-ratio profiles for NR1, NR2C, NR3A and NR2D in hippocampus and C, in cortex. See Figure S1 for key to figure color and scale details.

Figure S11 AMPA receptor components under the baseline developmental conditions. A. Heat map of gene profiles, showing non-significant changes in hippocampus, and repression of GLUR1 and GLUR2 in cortex. B. Detailed log2-ratio profiles for GLUR2 in hippocampus and C, for GLUR1 and GLUR2 in cortex. See Figure S1 for key to figure color and scale details.

Figure S12 GABA receptor components and glutamate decarboxylase 1 and 2 under the baseline developmental conditions. A. Heat map of gene profiles, showing generally non-significant changes. B. Detailed log2-ratio profiles for $GABA_A$-δ (GABRD), $GABA_A$-β1 (GABRB1) and KCC2 in hippocampus and C, in cortex. See Figure S1 for key to figure color and scale details.

Figure S13 Heat map of the differential response to hypoxic seizures for the genes in the *In vitro response (BIC or 4AP)* group. The group is defined by one of the clusters of enriched gene sets in the membership matrix of Figure 7. Colors represent log2-ratios of intensities of hypoxic seizure samples to that of time-matched controls (red

= positive, green = negative values, colors saturate at ±1). Arrows point to genes referred to in the main text.

Figure S14 Heat map of the differential response to hypoxic seizures for the genes in the *Hypoxia response* group. The group is defined by one of the clusters of enriched gene sets in the membership matrix of Figure 7. See Figure S13 for key to figure color and scale details.

Figure S15 Heat map of the differential response to hypoxic seizures for the genes in the *Glutamatergic/NMDA receptor I* group. The group is defined by one of the clusters of enriched gene sets in the membership matrix of Figure 7. See Figure S13 for key to figure color and scale details.

Figure S16 Heat map of the differential response to hypoxic seizures for the genes in the *Glutamatergic/NMDA receptor II* group. The group is defined by one of the clusters of enriched gene sets in the membership matrix of Figure 7. See Figure S13 for key to figure color and scale details.

Figure S17 Heat map of the differential response to hypoxic seizures for the genes in the *Synaptic* group. The group is defined by one of the clusters of enriched gene sets in the membership matrix of Figure 7. See Figure S13 for key to figure color and scale details.

Figure S18 Heat map of the differential response to hypoxic seizures for the genes in the *Axonal Guidance* group. The group is defined by one of the clusters of enriched gene sets in the membership matrix of Figure 7. See Figure S13 for key to figure color and scale details.

Figure S19 Heat map of the differential response to hypoxic seizures for the genes in the *Autism associated* group. The group is defined by one of the clusters of enriched gene sets in the membership matrix of Figure 7. See Figure S13 for key to figure color and scale details.

Figure S20 WNT3A target genes are enriched in the hypoxic seizure response profile. Enrichment analysis with the WNT3A target gene set[27] was performed against the 12 hour profile of transcriptional response to hypoxic seizures in hippocampus. A. KS plot showing enrichment profile. B. Table of the corresponding 15 leading-edge genes. Ratios are of gene expression intensities in hypoxic seizure sample to corresponding control.

Figure S21 Expression ratios as a function of time for selected IGF-1/PI3K/mTOR pathway genes. The gene name is indicated at the top of each figure. Time at the bottom refers to the duration following hypoxic seizures; the left-hand panels (labeled "hyp") display ratios of hypoxic seizure response to control; the right-hand panels (labeled "hyp + NBQX") displays ratios of response to combined hypoxic seizures and NBQX treatment, to control. The expression ratio R = 1 (no fold change) is indicated

by the black horizontal lines. A. IRS1 displays ~ 1.6 fold induction at 12 h. B. AKT3 is very moderately but significantly induced over the time course. C and D. IGF1 and BDNF are not significantly induced.

Figure S22 Heat map of differential expression for the NBQX-responsive genes. The profiles of the 257 genes, each of which is differentially regulated by both hypoxic seizures (HS) relative to control, and by HS+NBQX relative to HS, are shown (these genes form a proper subset of the 1,399 HS-responsive genes displayed in Figure 6). Log2 ratios of gene expression between time-matched samples are shown for the ratio combinations indicated on top. Colors saturate for log2-ratio = ±1.

Figure S23 Expression ratios as a function of time for 11 Wnt genes represented on the microarray. The gene name is indicated at the top of each figure. Time at the bottom refers to the duration following hypoxic seizures; the left-hand panels (labeled "hyp") display ratios of hypoxic seizure response to control; the right-hand panels (labeled "hyp + NBQX") displays ratios of response to combined hypoxic seizures and NBQX treatment, to control. The expression ratio R = 1 (no fold change) is indicated by the black horizontal lines. Only WNT5A and WNT2 are significantly induced.

Figure S1

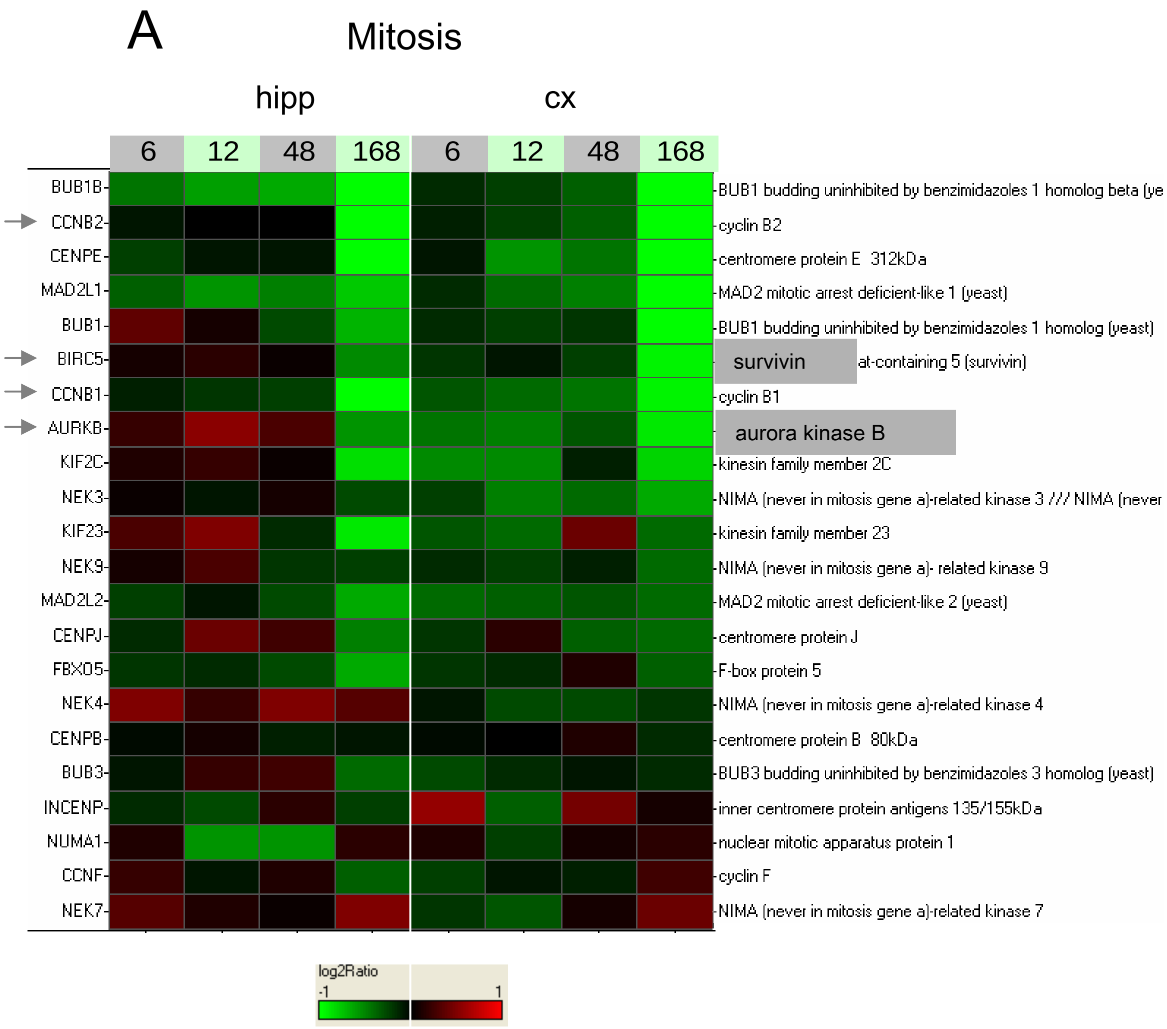
A
Mitosis
hipp
cx
6
12
48
168
6
12
48
168
BUB1B
CCNB2
CENPE
MAD2L1
BUB1
BIRC5
CCNB1
AURKB
KIF2C
NEK3
KIF23
NEK9
MAD2L2
CENPJ
FBXO5
NEK4
CENPB
BUB3
INCENP
NUMA1
CCNF
NEK7
BUB1 budding uninhibited by benzimidazoles 1 homolog beta (ye
cyclin B2
centromere protein E 312kDa
MAD2 mitotic arrest deficient-like 1 (yeast)
BUB1 budding uninhibited by benzimidazoles 1 homolog (yeast)
survivin
at-containing 5 (survivin)
cyclin B1
aurora kinase B
kinesin family member 2C
NIMA (never in mitosis gene a)-related kinase 3 /// NIMA (never
kinesin family member 23
NIMA (never in mitosis gene a)- related kinase 9
MAD2 mitotic arrest deficient-like 2 (yeast)
centromere protein J
F-box protein 5
NIMA (never in mitosis gene a)-related kinase 4
centromere protein B 80kDa
BUB3 budding uninhibited by benzimidazoles 3 homolog (yeast)
inner centromere protein antigens 135/155kDa
nuclear mitotic apparatus protein 1
cyclin F
NIMA (never in mitosis gene a)-related kinase 7
log2Ratio
-1
1

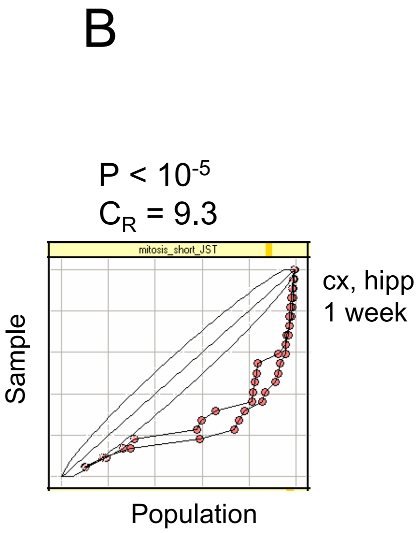
B
P < 10^-5
C_R = 9.3
mitosis_short_JST
cx, hipp
1 week
Sample
Population

Figure S2

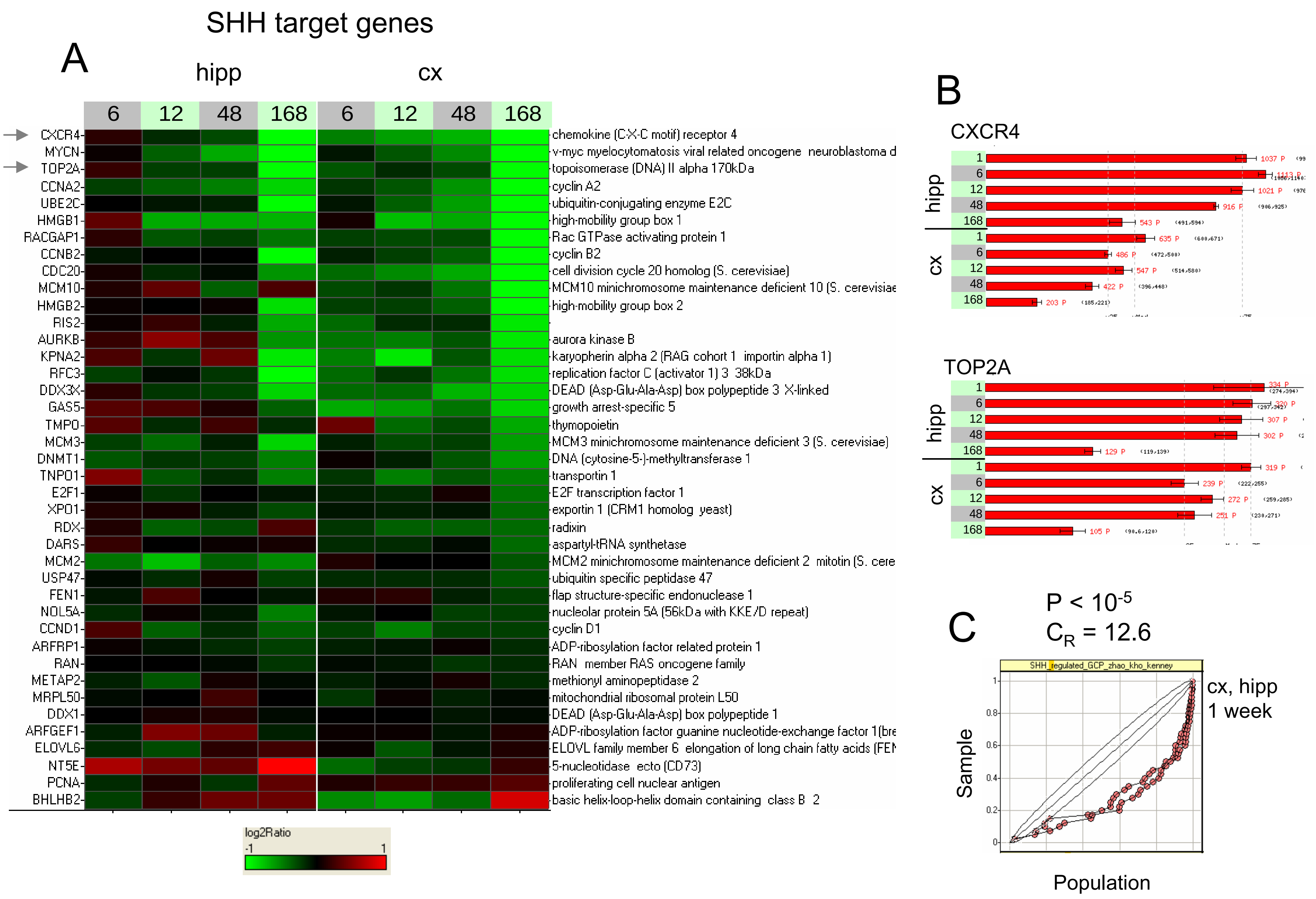
SHH target genes
A
hipp
cx
6
12
48
168
6
12
48
168
CXCR4 chemokine (C-X-C motif) receptor 4
MYCN v-myc myelocytomatosis viral related oncogene neuroblastoma d
TOP2A topoisomerase (DNA) II alpha 170kDa
CCNA2 cyclin A2
UBE2C ubiquitin-conjugating enzyme E2C
HMGB1 high-mobility group box 1
RACGAP1 Rac GTPase activating protein 1
CCNB2 cyclin B2
CDC20 cell division cycle 20 homolog (S. cerevisiae)
MCM10 MCM10 minichromosome maintenance deficient 10 (S. cerevisiae
HMGB2 high-mobility group box 2
RIS2
AURKB aurora kinase B
KPNA2 karyopherin alpha 2 (RAG cohort 1 importin alpha 1)
RFC3 replication factor C (activator 1) 3 38kDa
DDX3X DEAD (Asp-Glu-Ala-Asp) box polypeptide 3 X-linked
GAS5 growth arrest-specific 5
TMPO thymopoietin
MCM3 MCM3 minichromosome maintenance deficient 3 (S. cerevisiae)
DNMT1 DNA (cytosine-5-)-methyltransferase 1
TNPO1 transportin 1
E2F1 E2F transcription factor 1
XPO1 exportin 1 (CRM1 homolog yeast)
RDX radixin
DARS aspartyl-tRNA synthetase
MCM2 MCM2 minichromosome maintenance deficient 2 mitotin (S. cere
USP47 ubiquitin specific peptidase 47
FEN1 flap structure-specific endonuclease 1
NOL5A nucleolar protein 5A (56kDa with KKE/D repeat)
CCND1 cyclin D1
ARFRP1 ADP-ribosylation factor related protein 1
RAN RAN member RAS oncogene family
METAP2 methionyl aminopeptidase 2
MRPL50 mitochondrial ribosomal protein L50
DDX1 DEAD (Asp-Glu-Ala-Asp) box polypeptide 1
ARFGEF1 ADP-ribosylation factor guanine nucleotide-exchange factor 1(bre
ELOVL6 ELOVL family member 6 elongation of long chain fatty acids (FEN
NT5E 5-nucleotidase ecto (CD73)
PCNA proliferating cell nuclear antigen
BHLHB2 basic helix-loop-helix domain containing class B 2
log2Ratio
-1
1
B
CXCR4
hipp
cx
1
6
12
48
168
1037 P
1113 P
1021 P
916 P
543 P
635 P
486 P
547 P
422 P
203 P
TOP2A
334 P
320 P
307 P
302 P
129 P
319 P
239 P
272 P
251 P
105 P
C
P < 10^-5
C_R = 12.6
SHH_regulated_GCP_zhao_kho_kenney
cx, hipp
1 week
Sample
Population
1
0.8
0.6
0.4
0.2
0

Figure S3

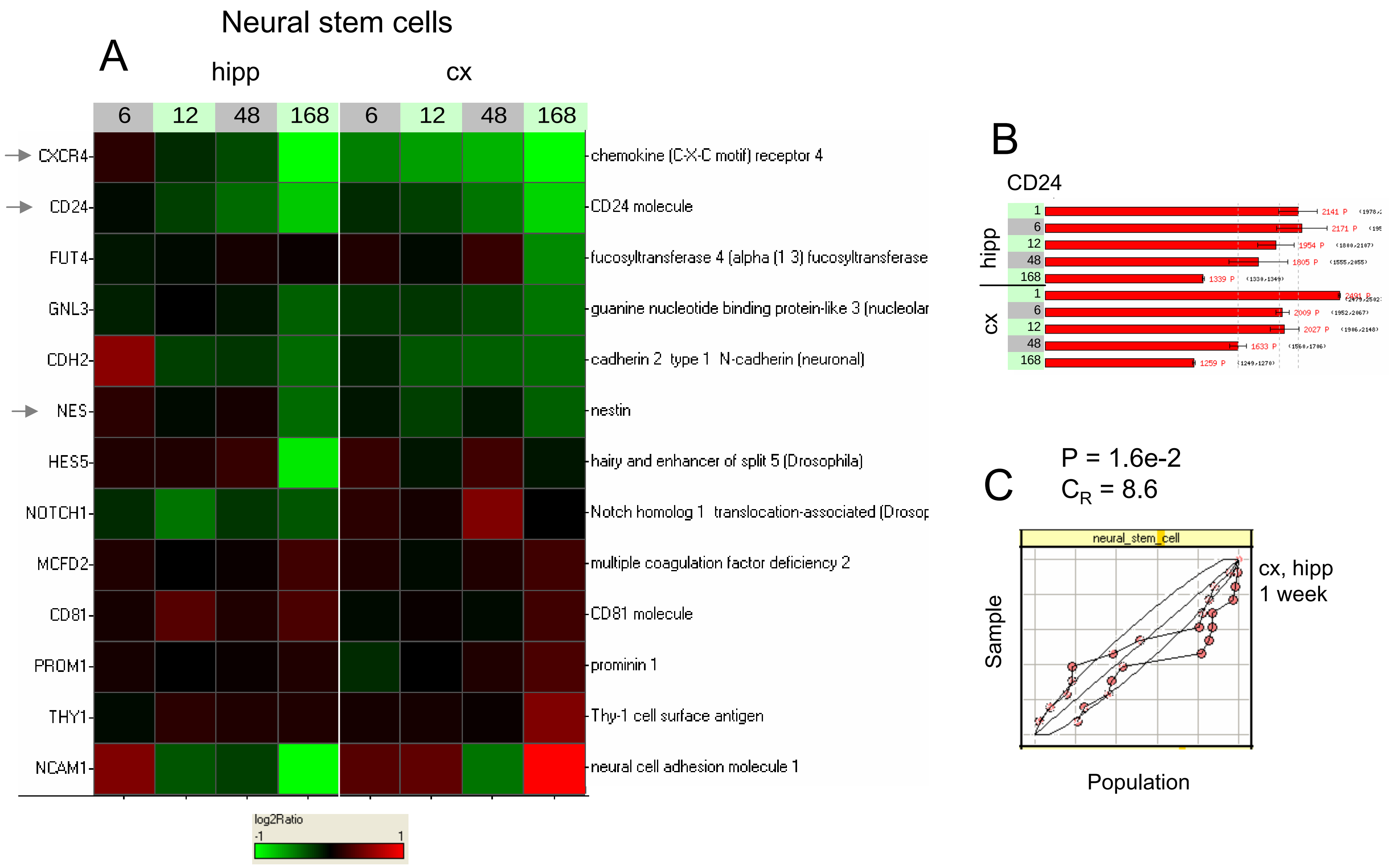
Neural stem cells
A
hipp
cx
6
12
48
168
6
12
48
168
CXCR4
CD24
FUT4
GNL3
CDH2
NES
HES5
NOTCH1
MCFD2
CD81
PROM1
THY1
NCAM1
chemokine (C-X-C motif) receptor 4
CD24 molecule
fucosyltransferase 4 (alpha (1 3) fucosyltransferase
guanine nucleotide binding protein-like 3 (nucleolar
cadherin 2 type 1 N-cadherin (neuronal)
nestin
hairy and enhancer of split 5 (Drosophila)
Notch homolog 1 translocation-associated (Drosop
multiple coagulation factor deficiency 2
CD81 molecule
prominin 1
Thy-1 cell surface antigen
neural cell adhesion molecule 1
log2Ratio
-1
1
B
CD24
hipp
cx
1
6
12
48
168
2141 P
2171 P
1954 P
1805 P
1339 P
2009 P
2027 P
1633 P
1259 P
C
P = 1.6e-2
C_R = 8.6
neural_stem_cell
cx, hipp
1 week
Sample
Population

Figure S4

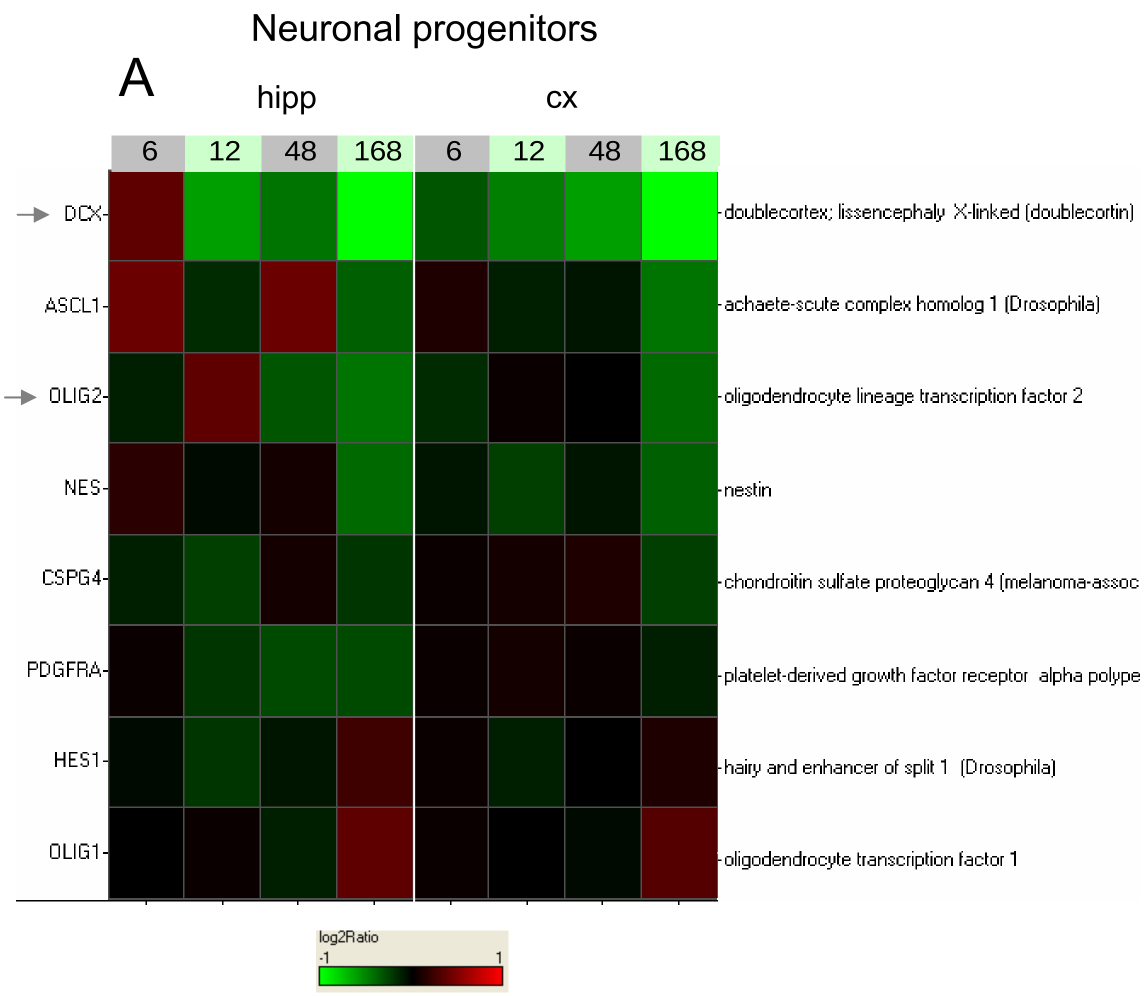
Neuronal progenitors
A
hipp
cx
6
12
48
168
6
12
48
168
DCX
ASCL1
OLIG2
NES
CSPG4
PDGFRA
HES1
OLIG1
doublecortex; lissencephaly X-linked (doublecortin)
achaete-scute complex homolog 1 (Drosophila)
oligodendrocyte lineage transcription factor 2
nestin
chondroitin sulfate proteoglycan 4 (melanoma-assoc
platelet-derived growth factor receptor alpha polype
hairy and enhancer of split 1 (Drosophila)
oligodendrocyte transcription factor 1
log2Ratio
-1
1

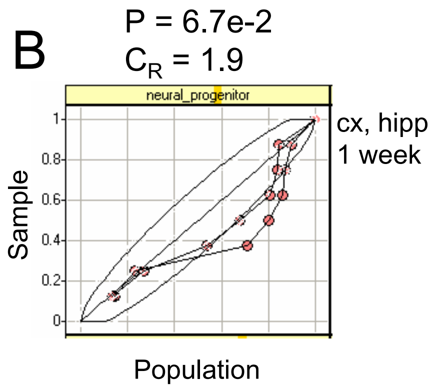
B
P = 6.7e-2
C_R = 1.9
neural_progenitor
cx, hipp
1 week
Sample
Population
1
0.8
0.6
0.4
0.2
0

Figure S5

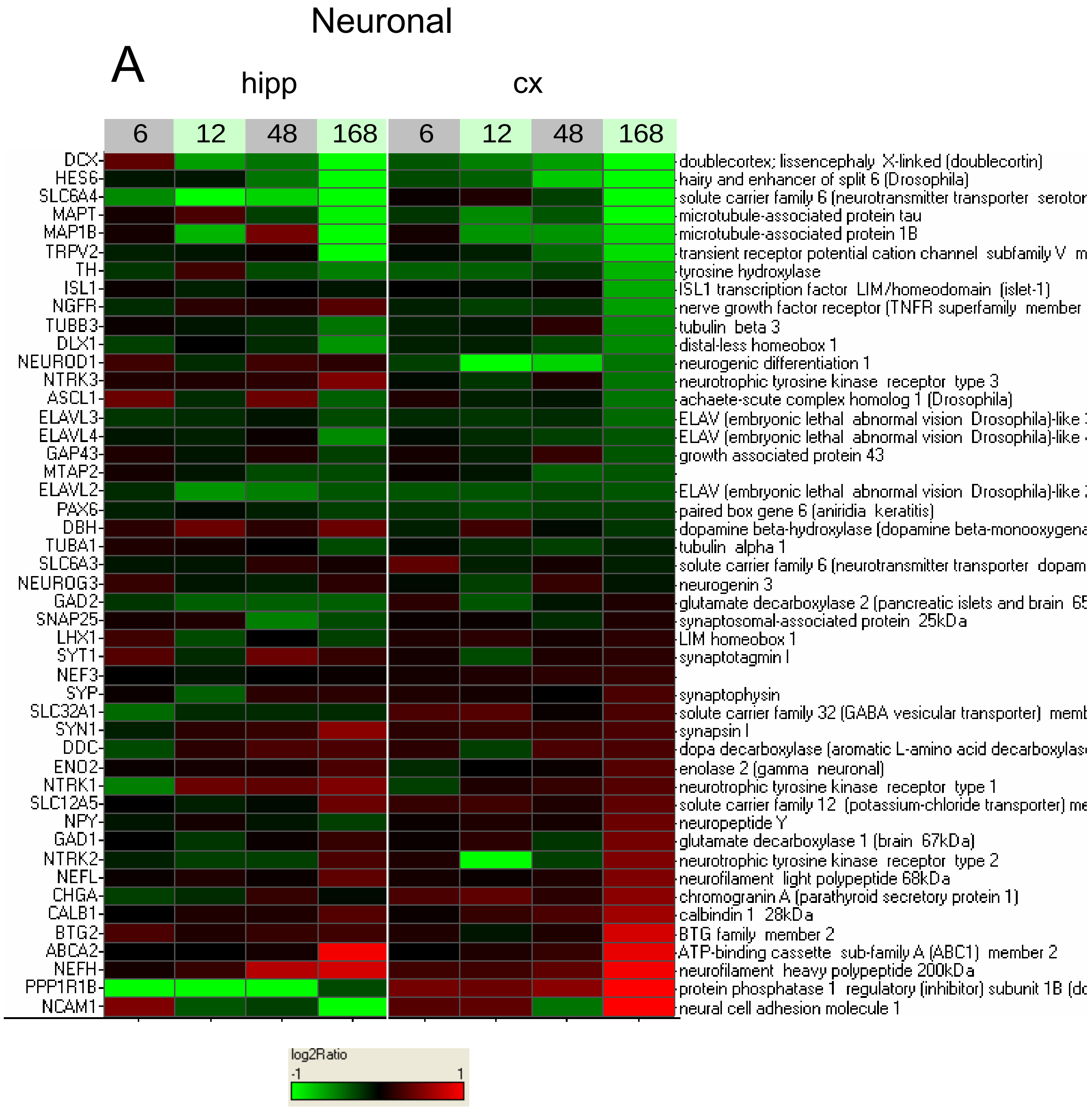

Neuronal
A
hipp
cx
6
12
48
168
DCX
HES6
SLC6A4
MAPT
MAP1B
TRPV2
TH
ISL1
NGFR
TUBB3
DLX1
NEUROD1
NTRK3
ASCL1
ELAVL3
ELAVL4
GAP43
MTAP2
ELAVL2
PAX6
DBH
TUBA1
SLC6A3
NEUROG3
GAD2
SNAP25
LHX1
SYT1
NEF3
SYP
SLC32A1
SYN1
DDC
ENO2
NTRK1
SLC12A5
NPY
GAD1
NTRK2
NEFL
CHGA
CALB1
BTG2
ABCA2
NEFH
PPP1R1B
NCAM1
doublecortex; lissencephaly X-linked (doublecortin)
hairy and enhancer of split 6 (Drosophila)
solute carrier family 6 (neurotransmitter transporter serotor
microtubule-associated protein tau
microtubule-associated protein 1B
transient receptor potential cation channel subfamily V m
tyrosine hydroxylase
ISL1 transcription factor LIM/homeodomain (islet-1)
nerve growth factor receptor (TNFR superfamily member
tubulin beta 3
distal-less homeobox 1
neurogenic differentiation 1
neurotrophic tyrosine kinase receptor type 3
achaete-scute complex homolog 1 (Drosophila)
ELAV (embryonic lethal abnormal vision Drosophila)-like
ELAV (embryonic lethal abnormal vision Drosophila)-like
growth associated protein 43
ELAV (embryonic lethal abnormal vision Drosophila)-like
paired box gene 6 (aniridia keratitis)
dopamine beta-hydroxylase (dopamine beta-monooxygena
tubulin alpha 1
solute carrier family 6 (neurotransmitter transporter dopam
neurogenin 3
glutamate decarboxylase 2 (pancreatic islets and brain 65
synaptosomal-associated protein 25kDa
LIM homeobox 1
synaptotagmin I
synaptophysin
solute carrier family 32 (GABA vesicular transporter) memt
synapsin I
dopa decarboxylase (aromatic L-amino acid decarboxylas
enolase 2 (gamma neuronal)
neurotrophic tyrosine kinase receptor type 1
solute carrier family 12 (potassium-chloride transporter) me
neuropeptide Y
glutamate decarboxylase 1 (brain 67kDa)
neurotrophic tyrosine kinase receptor type 2
neurofilament light polypeptide 68kDa
chromogranin A (parathyroid secretory protein 1)
calbindin 1 28kDa
BTG family member 2
ATP-binding cassette sub-family A (ABC1) member 2
neurofilament heavy polypeptide 200kDa
protein phosphatase 1 regulatory (inhibitor) subunit 1B (dc
neural cell adhesion molecule 1
log2Ratio
-1
1

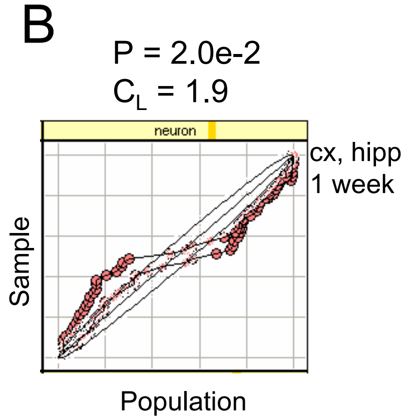

B
P = 2.0e-2
C_L = 1.9
neuron
cx, hipp
1 week
Sample
Population

Figure S6

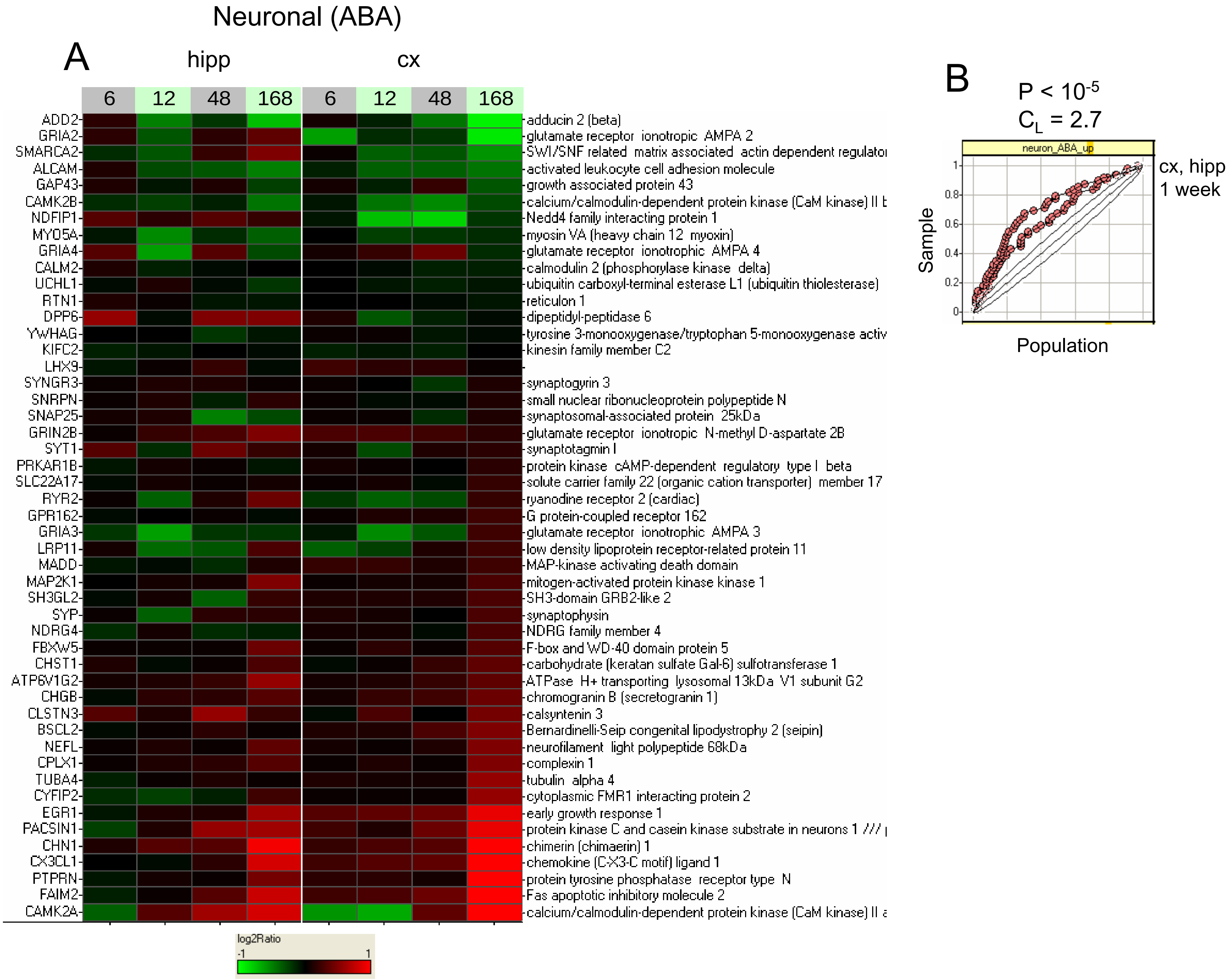

Neuronal (ABA)
A
hipp
cx
6
12
48
168
6
12
48
168
ADD2 - adducin 2 (beta)
GRIA2 - glutamate receptor ionotropic AMPA 2
SMARCA2 - SWI/SNF related matrix associated actin dependent regulatoı
ALCAM - activated leukocyte cell adhesion molecule
GAP43 - growth associated protein 43
CAMK2B - calcium/calmodulin-dependent protein kinase (CaM kinase) II b
NDFIP1 - Nedd4 family interacting protein 1
MYO5A - myosin VA (heavy chain 12 myoxin)
GRIA4 - glutamate receptor ionotrophic AMPA 4
CALM2 - calmodulin 2 (phosphorylase kinase delta)
UCHL1 - ubiquitin carboxyl-terminal esterase L1 (ubiquitin thiolesterase)
RTN1 - reticulon 1
DPP6 - dipeptidyl-peptidase 6
YWHAG - tyrosine 3-monooxygenase/tryptophan 5-monooxygenase activ
KIFC2 - kinesin family member C2
LHX9 -
SYNGR3 - synaptogyrin 3
SNRPN - small nuclear ribonucleoprotein polypeptide N
SNAP25 - synaptosomal-associated protein 25kDa
GRIN2B - glutamate receptor ionotropic N-methyl D-aspartate 2B
SYT1 - synaptotagmin I
PRKAR1B - protein kinase cAMP-dependent regulatory type I beta
SLC22A17 - solute carrier family 22 (organic cation transporter) member 17
RYR2 - ryanodine receptor 2 (cardiac)
GPR162 - G protein-coupled receptor 162
GRIA3 - glutamate receptor ionotrophic AMPA 3
LRP11 - low density lipoprotein receptor-related protein 11
MADD - MAP-kinase activating death domain
MAP2K1 - mitogen-activated protein kinase kinase 1
SH3GL2 - SH3-domain GRB2-like 2
SYP - synaptophysin
NDRG4 - NDRG family member 4
FBXW5 - F-box and WD-40 domain protein 5
CHST1 - carbohydrate (keratan sulfate Gal-6) sulfotransferase 1
ATP6V1G2 - ATPase H+ transporting lysosomal 13kDa V1 subunit G2
CHGB - chromogranin B (secretogranin 1)
CLSTN3 - calsyntenin 3
BSCL2 - Bernardinelli-Seip congenital lipodystrophy 2 (seipin)
NEFL - neurofilament light polypeptide 68kDa
CPLX1 - complexin 1
TUBA4 - tubulin alpha 4
CYFIP2 - cytoplasmic FMR1 interacting protein 2
EGR1 - early growth response 1
PACSIN1 - protein kinase C and casein kinase substrate in neurons 1 /// ı
CHN1 - chimerin (chimaerin) 1
CX3CL1 - chemokine (C-X3-C motif) ligand 1
PTPRN - protein tyrosine phosphatase receptor type N
FAIM2 - Fas apoptotic inhibitory molecule 2
CAMK2A - calcium/calmodulin-dependent protein kinase (CaM kinase) II a
log2Ratio
-1
1
B
P < 10-5
CL = 2.7
neuron_ABA_up
cx, hipp
1 week
Sample
Population
1
0.8
0.6
0.4
0.2
0

Figure S7

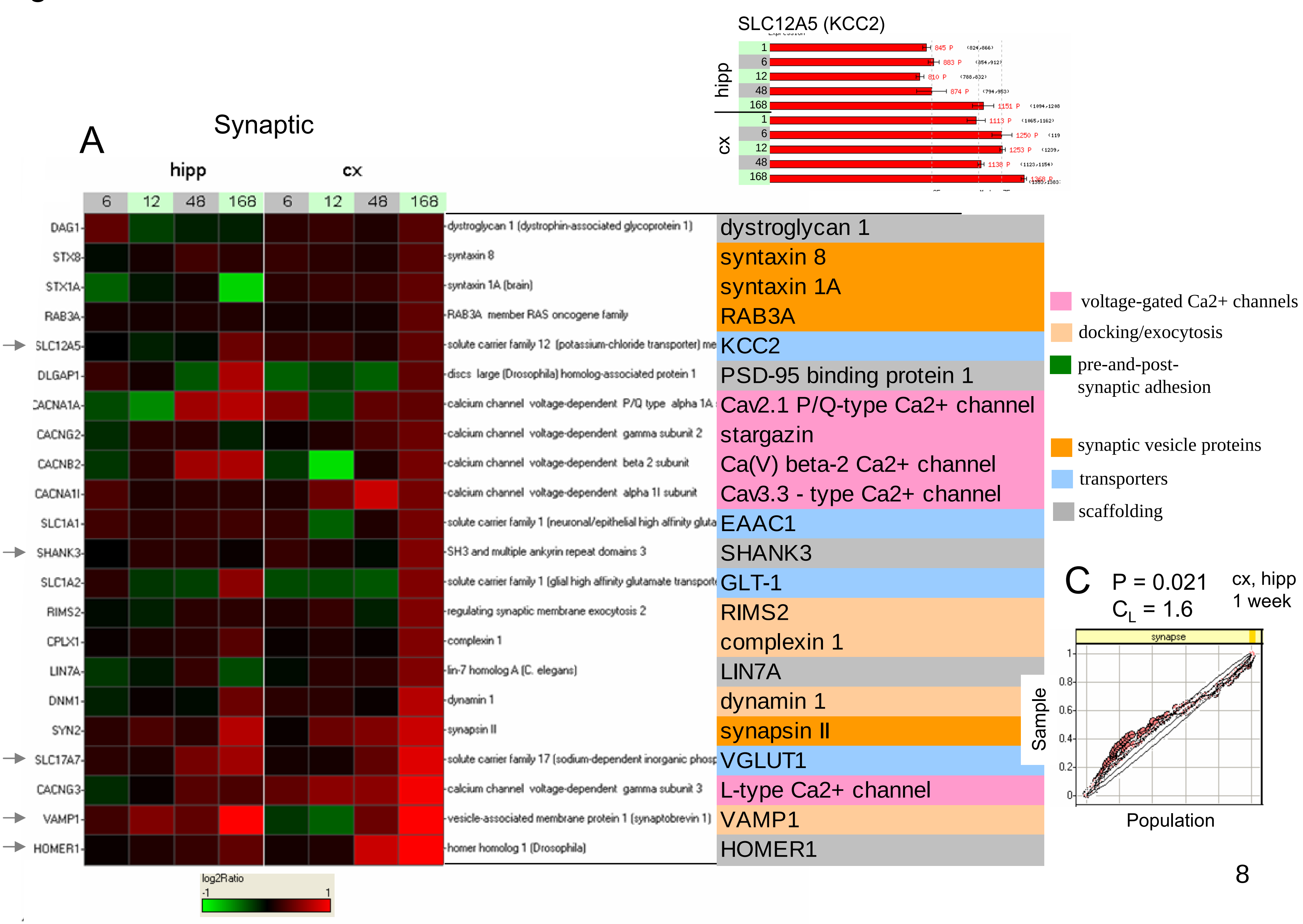
A
Synaptic
hipp
cx
6
12
48
168
DAG1
STX8
STX1A
RAB3A
SLC12A5
DLGAP1
CACNA1A
CACNG2
CACNB2
CACNA1I
SLC1A1
SHANK3
SLC1A2
RIMS2
CPLX1
LIN7A
DNM1
SYN2
SLC17A7
CACNG3
VAMP1
HOMER1
dystroglycan 1 (dystrophin-associated glycoprotein 1)
syntaxin 8
syntaxin 1A (brain)
RAB3A member RAS oncogene family
solute carrier family 12 (potassium-chloride transporter) me
discs large (Drosophila) homolog-associated protein 1
calcium channel voltage-dependent P/Q type alpha 1A
calcium channel voltage-dependent gamma subunit 2
calcium channel voltage-dependent beta 2 subunit
calcium channel voltage-dependent alpha 1I subunit
solute carrier family 1 (neuronal/epithelial high affinity gluta
SH3 and multiple ankyrin repeat domains 3
solute carrier family 1 (glial high affinity glutamate transporte
regulating synaptic membrane exocytosis 2
complexin 1
lin-7 homolog A (C. elegans)
dynamin 1
synapsin II
solute carrier family 17 (sodium-dependent inorganic phosp
calcium channel voltage-dependent gamma subunit 3
vesicle-associated membrane protein 1 (synaptobrevin 1)
homer homolog 1 (Drosophila)
dystroglycan 1
syntaxin 8
syntaxin 1A
RAB3A
KCC2
PSD-95 binding protein 1
Cav2.1 P/Q-type Ca2+ channel
stargazin
Ca(V) beta-2 Ca2+ channel
Cav3.3 - type Ca2+ channel
EAAC1
SHANK3
GLT-1
RIMS2
complexin 1
LIN7A
dynamin 1
synapsin II
VGLUT1
L-type Ca2+ channel
VAMP1
HOMER1
log2Ratio
-1
1
voltage-gated Ca2+ channels
docking/exocytosis
pre-and-post-synaptic adhesion
synaptic vesicle proteins
transporters
scaffolding
B
SLC12A5 (KCC2)
hipp
cx
1
6
12
48
168
845 P
883 P
810 P
874 P
1151 P
1113 P
1250 P
1253 P
1138 P
C
P = 0.021
C$_L$ = 1.6
cx, hipp
1 week
synapse
Sample
Population
0
0.2
0.4
0.6
0.8
1

Figure S8

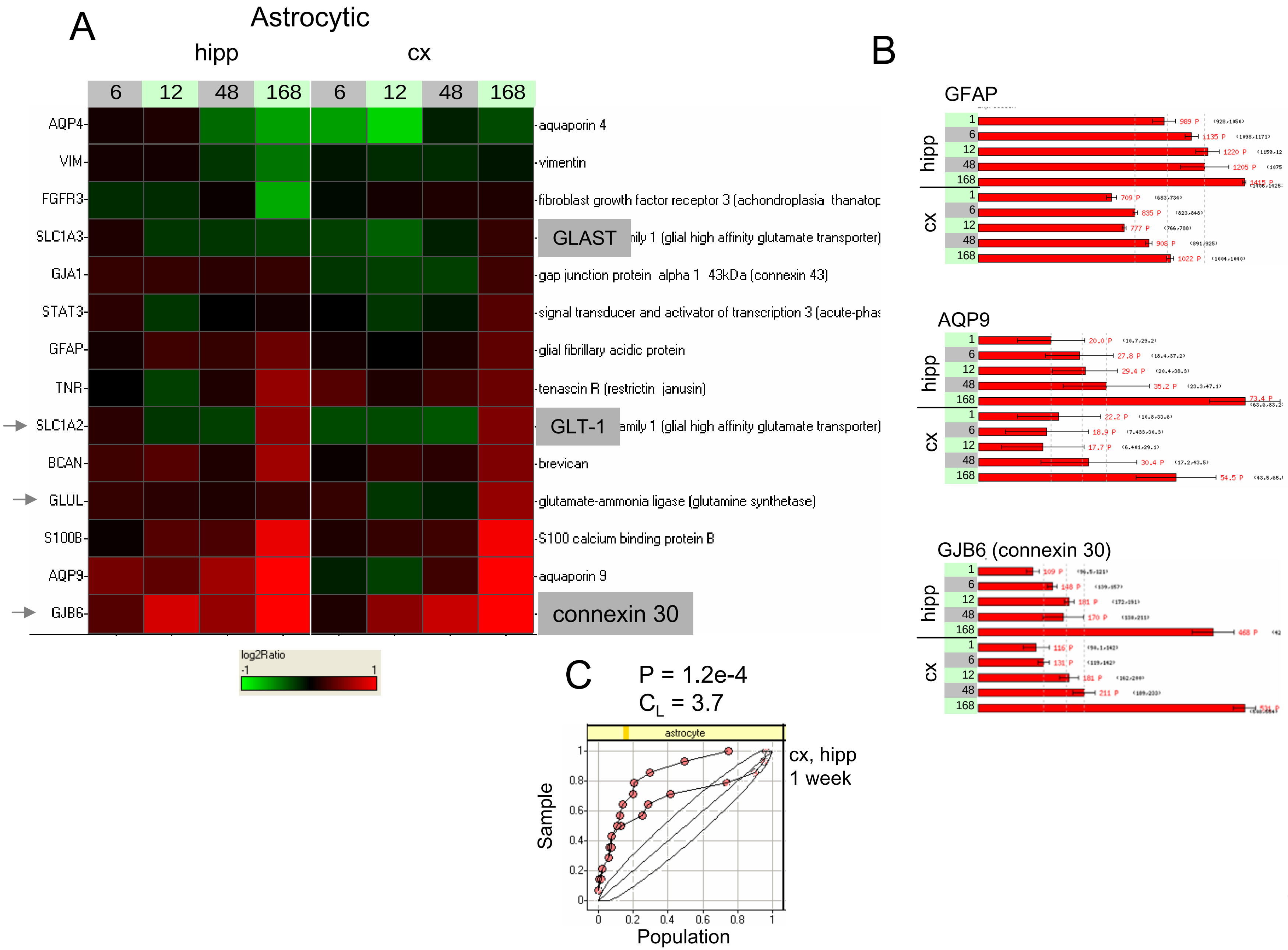

A
Astrocytic
hipp
cx
6
12
48
168
6
12
48
168
AQP4
VIM
FGFR3
SLC1A3
GJA1
STAT3
GFAP
TNR
SLC1A2
BCAN
GLUL
S100B
AQP9
GJB6
aquaporin 4
vimentin
fibroblast growth factor receptor 3 (achondroplasia thanatop
GLAST
ily 1 (glial high affinity glutamate transporter)
gap junction protein alpha 1 43kDa (connexin 43)
signal transducer and activator of transcription 3 (acute-phas
glial fibrillary acidic protein
tenascin R (restrictin janusin)
GLT-1
amily 1 (glial high affinity glutamate transporter)
brevican
glutamate-ammonia ligase (glutamine synthetase)
S100 calcium binding protein B
aquaporin 9
connexin 30
log2Ratio
-1
1
B
GFAP
AQP9
GJB6 (connexin 30)
hipp
cx
C
P = 1.2e-4
C_L = 3.7
astrocyte
cx, hipp
1 week
Sample
Population

Figure S9

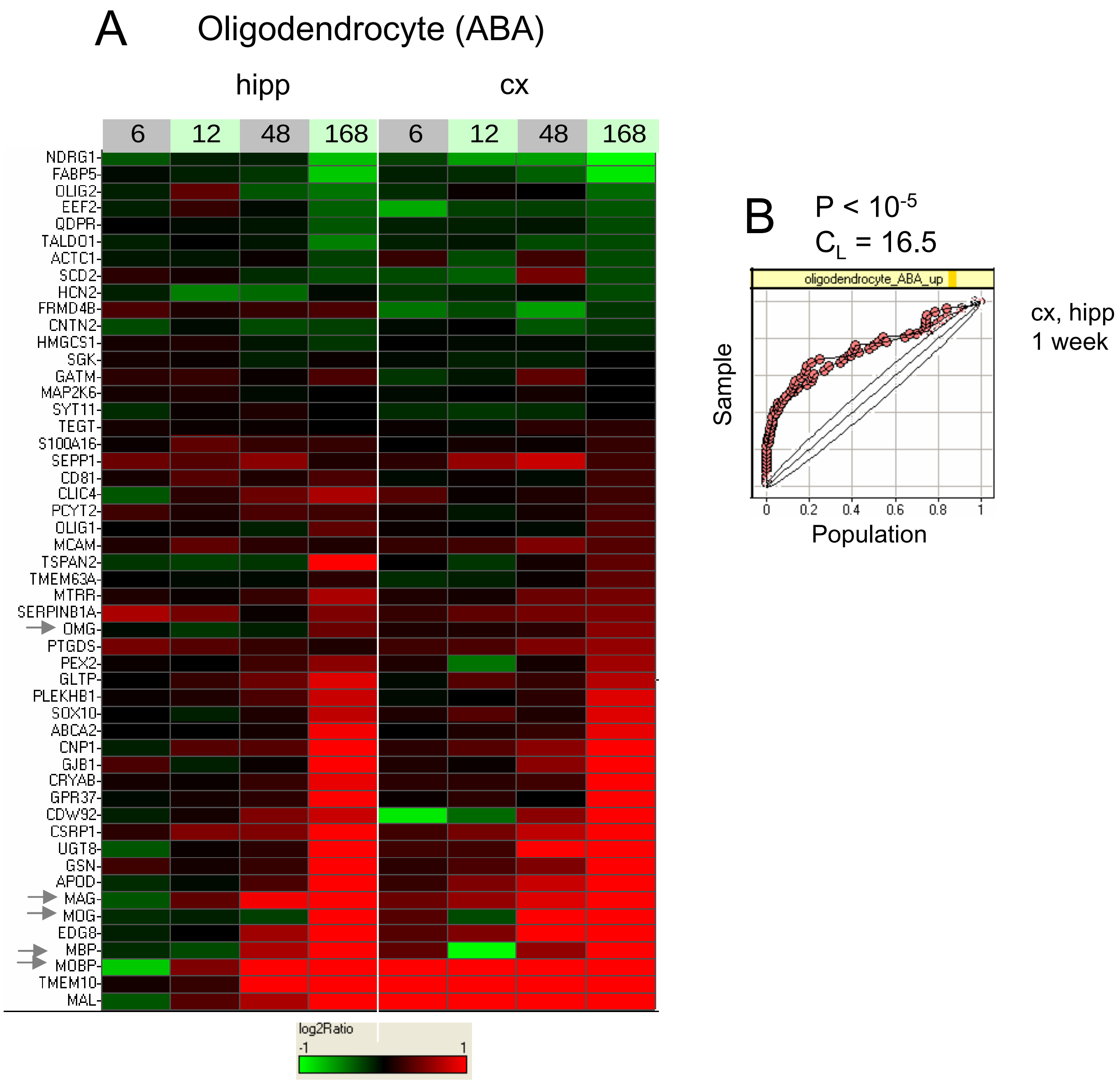
A
Oligodendrocyte (ABA)
hipp
cx
6
12
48
168
6
12
48
168
NDRG1
FABP5
OLIG2
EEF2
QDPR
TALDO1
ACTC1
SCD2
HCN2
FRMD4B
CNTN2
HMGCS1
SGK
GATM
MAP2K6
SYT11
TEGT
S100A16
SEPP1
CD81
CLIC4
PCYT2
OLIG1
MCAM
TSPAN2
TMEM63A
MTRR
SERPINB1A
OMG
PTGDS
PEX2
GLTP
PLEKHB1
SOX10
ABCA2
CNP1
GJB1
CRYAB
GPR37
CDW92
CSRP1
UGT8
GSN
APOD
MAG
MOG
EDG8
MBP
MOBP
TMEM10
MAL
log2Ratio
-1
1
B
P < 10-5
CL = 16.5
oligodendrocyte_ABA_up
Sample
Population
0
0.2
0.4
0.6
0.8
1
cx, hipp
1 week

Figure S10

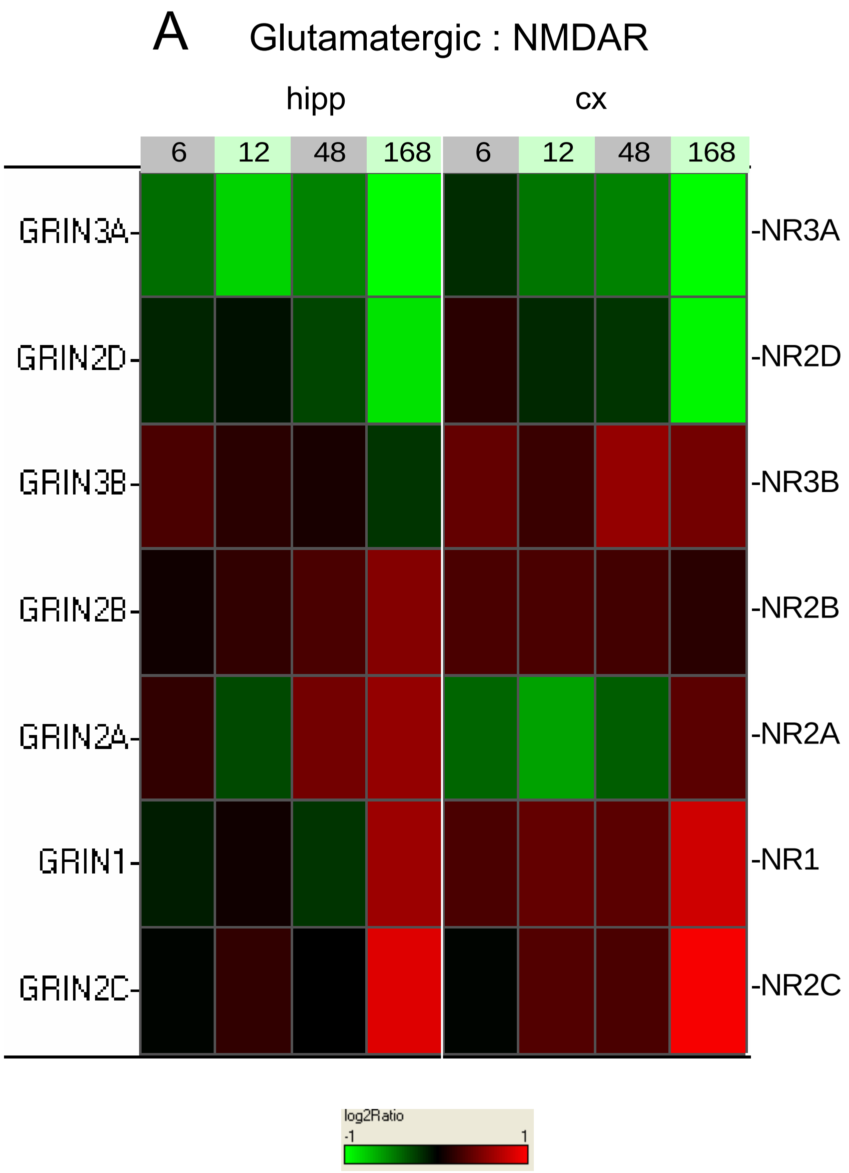

A
Glutamatergic : NMDAR
hipp
cx
6
12
48
168
6
12
48
168
GRIN3A
GRIN2D
GRIN3B
GRIN2B
GRIN2A
GRIN1
GRIN2C
-NR3A
-NR2D
-NR3B
-NR2B
-NR2A
-NR1
-NR2C
log2Ratio
-1
1

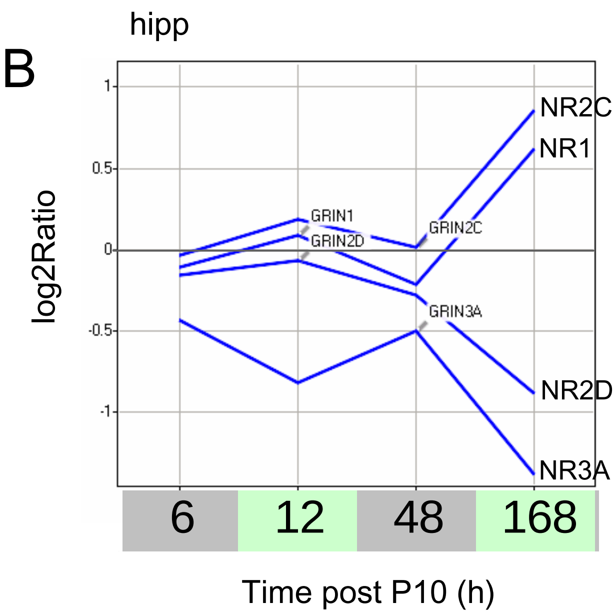

hipp
B
log2Ratio
1
0.5
0
-0.5
-1
GRIN1
GRIN2D
GRIN2C
GRIN3A
NR2C
NR1
NR2D
NR3A
6
12
48
168
Time post P10 (h)

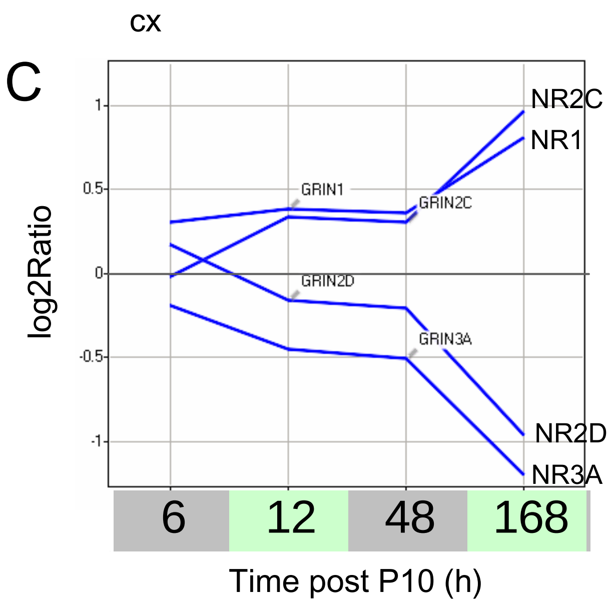

cx
C
log2Ratio
1
0.5
0
-0.5
-1
GRIN1
GRIN2C
GRIN2D
GRIN3A
NR2C
NR1
NR2D
NR3A
6
12
48
168
Time post P10 (h)

Figure S11

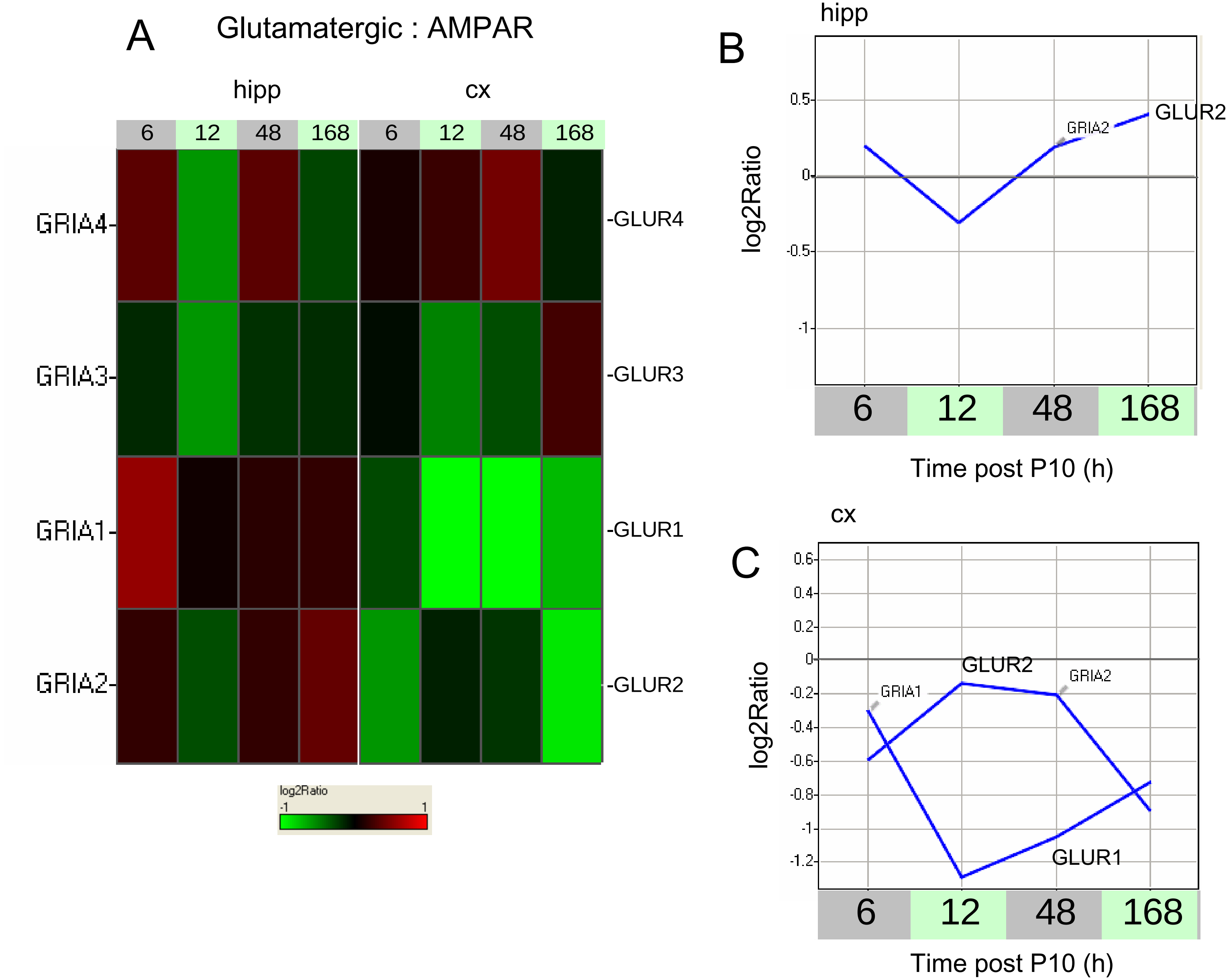

A
Glutamatergic : AMPAR
hipp
cx
6
12
48
168
6
12
48
168
GRIA4
GRIA3
GRIA1
GRIA2
GLUR4
GLUR3
GLUR1
GLUR2
log2Ratio
-1
1
B
hipp
0.5
0
-0.5
-1
log2Ratio
GRIA2
GLUR2
6
12
48
168
Time post P10 (h)
cx
C
0.6
0.4
0.2
0
-0.2
-0.4
-0.6
-0.8
-1
-1.2
log2Ratio
GLUR2
GRIA1
GRIA2
GLUR1
6
12
48
168
Time post P10 (h)

Figure S12

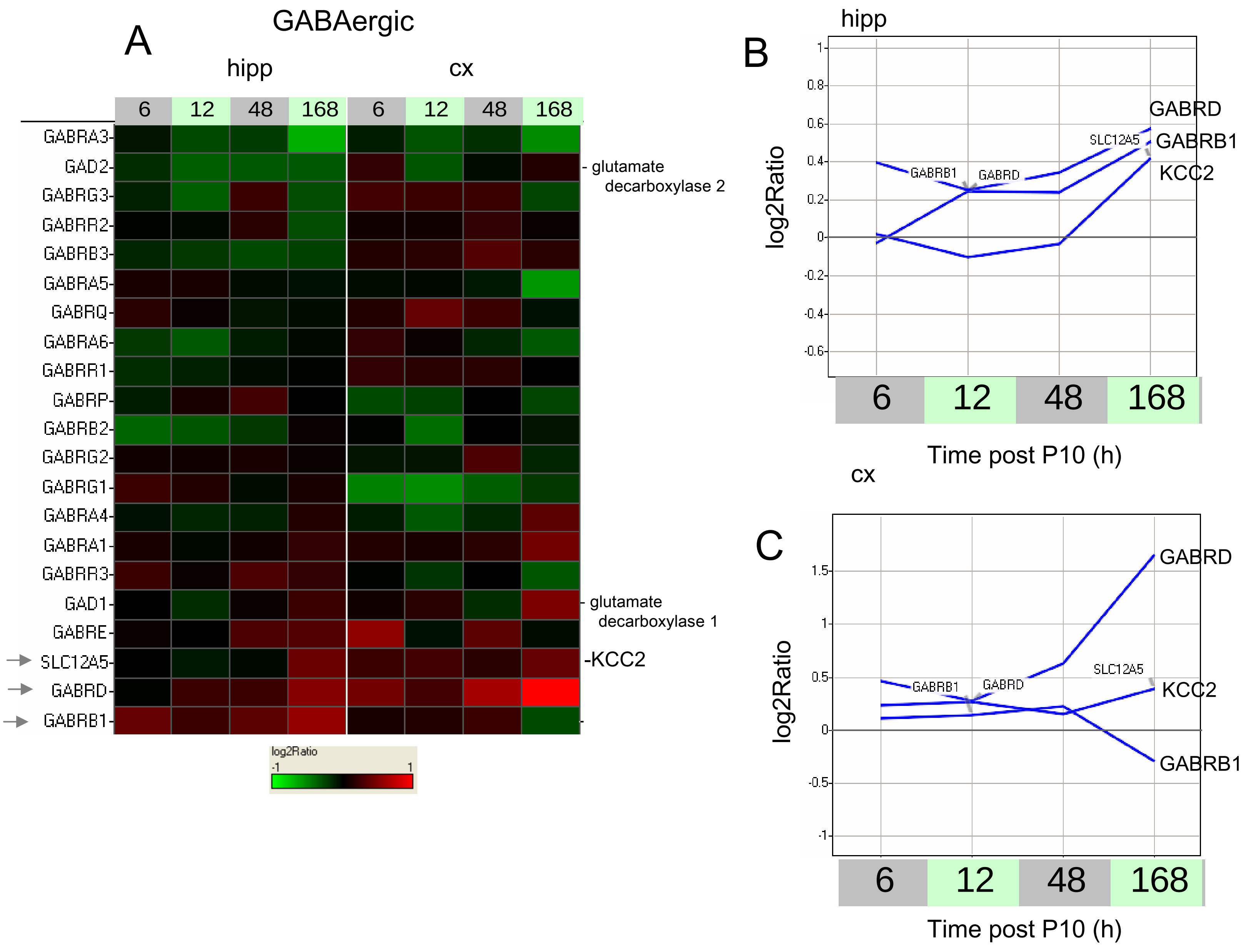

A
GABAergic
hipp
cx
6
12
48
168
6
12
48
168
GABRA3
GAD2
GABRG3
GABRR2
GABRB3
GABRA5
GABRQ
GABRA6
GABRR1
GABRP
GABRB2
GABRG2
GABRG1
GABRA4
GABRA1
GABRR3
GAD1
GABRE
SLC12A5
GABRD
GABRB1
glutamate decarboxylase 2
glutamate decarboxylase 1
KCC2
log2Ratio
-1
1
B
hipp
log2Ratio
GABRD
GABRB1
KCC2
SLC12A5
GABRB1
GABRD
6
12
48
168
Time post P10 (h)
C
cx
log2Ratio
GABRD
KCC2
GABRB1
SLC12A5
GABRB1
GABRD
6
12
48
168
Time post P10 (h)

Figure S13

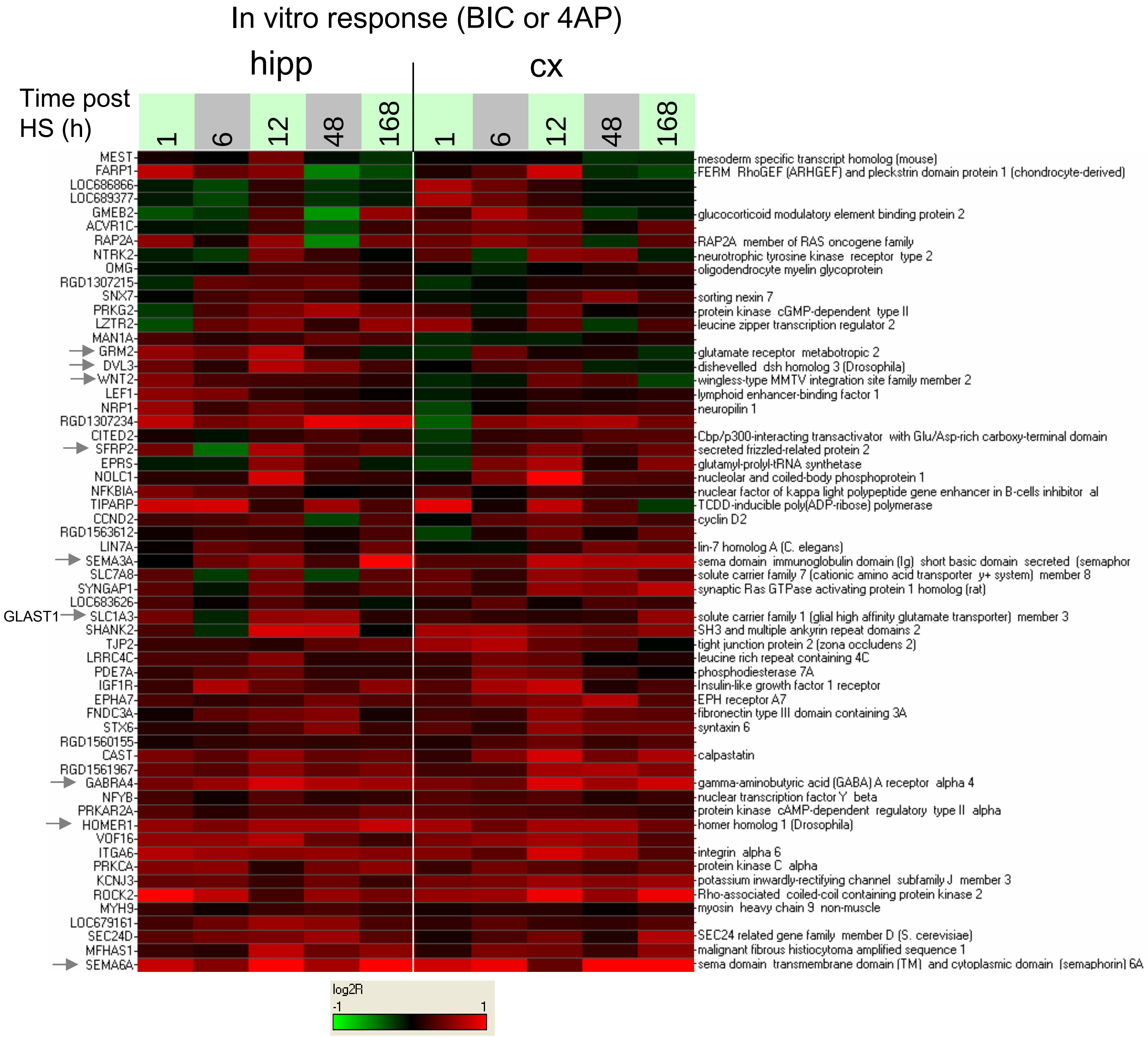
In vitro response (BIC or 4AP)
hipp
cx
Time post HS (h)
1
6
12
48
168
1
6
12
48
168
MEST - mesoderm specific transcript homolog (mouse)
FARP1 - FERM RhoGEF (ARHGEF) and pleckstrin domain protein 1 (chondrocyte-derived)
LOC686866
LOC689377
GMEB2 - glucocorticoid modulatory element binding protein 2
ACVR1C
RAP2A - RAP2A member of RAS oncogene family
NTRK2 - neurotrophic tyrosine kinase receptor type 2
OMG - oligodendrocyte myelin glycoprotein
RGD1307215
SNX7 - sorting nexin 7
PRKG2 - protein kinase cGMP-dependent type II
LZTR2 - leucine zipper transcription regulator 2
MAN1A
GRM2 - glutamate receptor metabotropic 2
DVL3 - dishevelled dsh homolog 3 (Drosophila)
WNT2 - wingless-type MMTV integration site family member 2
LEF1 - lymphoid enhancer-binding factor 1
NRP1 - neuropilin 1
RGD1307234
CITED2 - Cbp/p300-interacting transactivator with Glu/Asp-rich carboxy-terminal domain
SFRP2 - secreted frizzled-related protein 2
EPRS - glutamyl-prolyl-tRNA synthetase
NOLC1 - nucleolar and coiled-body phosphoprotein 1
NFKBIA - nuclear factor of kappa light polypeptide gene enhancer in B-cells inhibitor al
TIPARP - TCDD-inducible poly(ADP-ribose) polymerase
CCND2 - cyclin D2
RGD1563612
LIN7A - lin-7 homolog A (C. elegans)
SEMA3A - sema domain immunoglobulin domain (Ig) short basic domain secreted (semaphor
SLC7A8 - solute carrier family 7 (cationic amino acid transporter y+ system) member 8
SYNGAP1 - synaptic Ras GTPase activating protein 1 homolog (rat)
LOC683626
GLAST1
SLC1A3 - solute carrier family 1 (glial high affinity glutamate transporter) member 3
SHANK2 - SH3 and multiple ankyrin repeat domains 2
TJP2 - tight junction protein 2 (zona occludens 2)
LRRC4C - leucine rich repeat containing 4C
PDE7A - phosphodiesterase 7A
IGF1R - Insulin-like growth factor 1 receptor
EPHA7 - EPH receptor A7
FNDC3A - fibronectin type III domain containing 3A
STX6 - syntaxin 6
RGD1560155
CAST - calpastatin
RGD1561967
GABRA4 - gamma-aminobutyric acid (GABA) A receptor alpha 4
NFYB - nuclear transcription factor Y beta
PRKAR2A - protein kinase cAMP-dependent regulatory type II alpha
HOMER1 - homer homolog 1 (Drosophila)
VOF16
ITGA6 - integrin alpha 6
PRKCA - protein kinase C alpha
KCNJ3 - potassium inwardly-rectifying channel subfamily J member 3
ROCK2 - Rho-associated coiled-coil containing protein kinase 2
MYH9 - myosin heavy chain 9 non-muscle
LOC679161
SEC24D - SEC24 related gene family member D (S. cerevisiae)
MFHAS1 - malignant fibrous histiocytoma amplified sequence 1
SEMA6A - sema domain transmembrane domain (TM) and cytoplasmic domain (semaphorin) 6A
log2R
-1
1

Figure S14

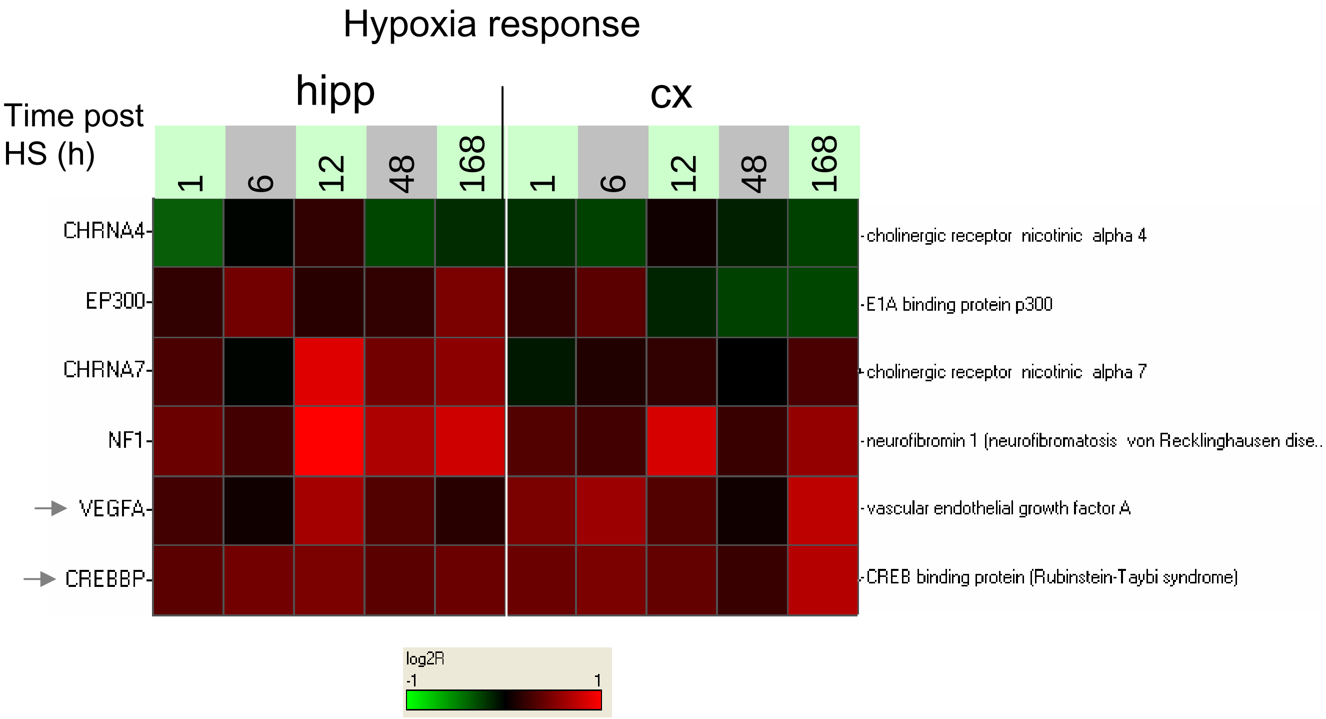
Hypoxia response
hipp
cx
Time post HS (h)
1
6
12
48
168
1
6
12
48
168
CHRNA4
EP300
CHRNA7
NF1
VEGFA
CREBBP
cholinergic receptor nicotinic alpha 4
E1A binding protein p300
cholinergic receptor nicotinic alpha 7
neurofibromin 1 (neurofibromatosis von Recklinghausen dise..
vascular endothelial growth factor A
CREB binding protein (Rubinstein-Taybi syndrome)
log2R
-1
1

Figure S15

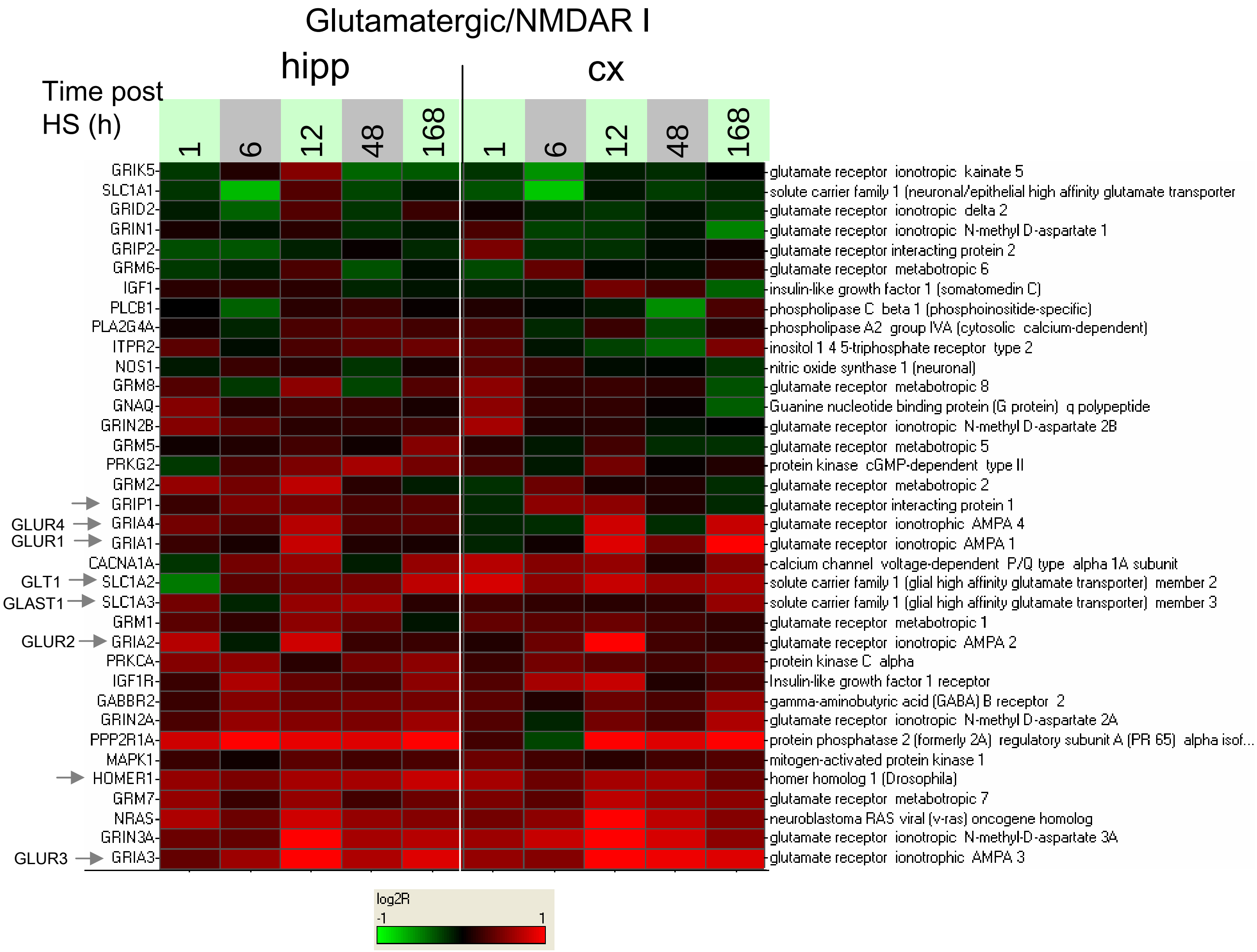

Glutamatergic/NMDAR I
hipp
cx
Time post HS (h)
1
6
12
48
168
1
6
12
48
168
GRIK5 glutamate receptor ionotropic kainate 5
SLC1A1 solute carrier family 1 (neuronal/epithelial high affinity glutamate transporter
GRID2 glutamate receptor ionotropic delta 2
GRIN1 glutamate receptor ionotropic N-methyl D-aspartate 1
GRIP2 glutamate receptor interacting protein 2
GRM6 glutamate receptor metabotropic 6
IGF1 insulin-like growth factor 1 (somatomedin C)
PLCB1 phospholipase C beta 1 (phosphoinositide-specific)
PLA2G4A phospholipase A2 group IVA (cytosolic calcium-dependent)
ITPR2 inositol 1 4 5-triphosphate receptor type 2
NOS1 nitric oxide synthase 1 (neuronal)
GRM8 glutamate receptor metabotropic 8
GNAQ Guanine nucleotide binding protein (G protein) q polypeptide
GRIN2B glutamate receptor ionotropic N-methyl D-aspartate 2B
GRM5 glutamate receptor metabotropic 5
PRKG2 protein kinase cGMP-dependent type II
GRM2 glutamate receptor metabotropic 2
GRIP1 glutamate receptor interacting protein 1
GLUR4 GRIA4 glutamate receptor ionotrophic AMPA 4
GLUR1 GRIA1 glutamate receptor ionotropic AMPA 1
CACNA1A calcium channel voltage-dependent P/Q type alpha 1A subunit
GLT1 SLC1A2 solute carrier family 1 (glial high affinity glutamate transporter) member 2
GLAST1 SLC1A3 solute carrier family 1 (glial high affinity glutamate transporter) member 3
GRM1 glutamate receptor metabotropic 1
GLUR2 GRIA2 glutamate receptor ionotropic AMPA 2
PRKCA protein kinase C alpha
IGF1R Insulin-like growth factor 1 receptor
GABBR2 gamma-aminobutyric acid (GABA) B receptor 2
GRIN2A glutamate receptor ionotropic N-methyl D-aspartate 2A
PPP2R1A protein phosphatase 2 (formerly 2A) regulatory subunit A (PR 65) alpha isof...
MAPK1 mitogen-activated protein kinase 1
HOMER1 homer homolog 1 (Drosophila)
GRM7 glutamate receptor metabotropic 7
NRAS neuroblastoma RAS viral (v-ras) oncogene homolog
GRIN3A glutamate receptor ionotropic N-methyl-D-aspartate 3A
GLUR3 GRIA3 glutamate receptor ionotrophic AMPA 3
log2R
-1
1

Figure S16

Glutamatergic/NMDAR II

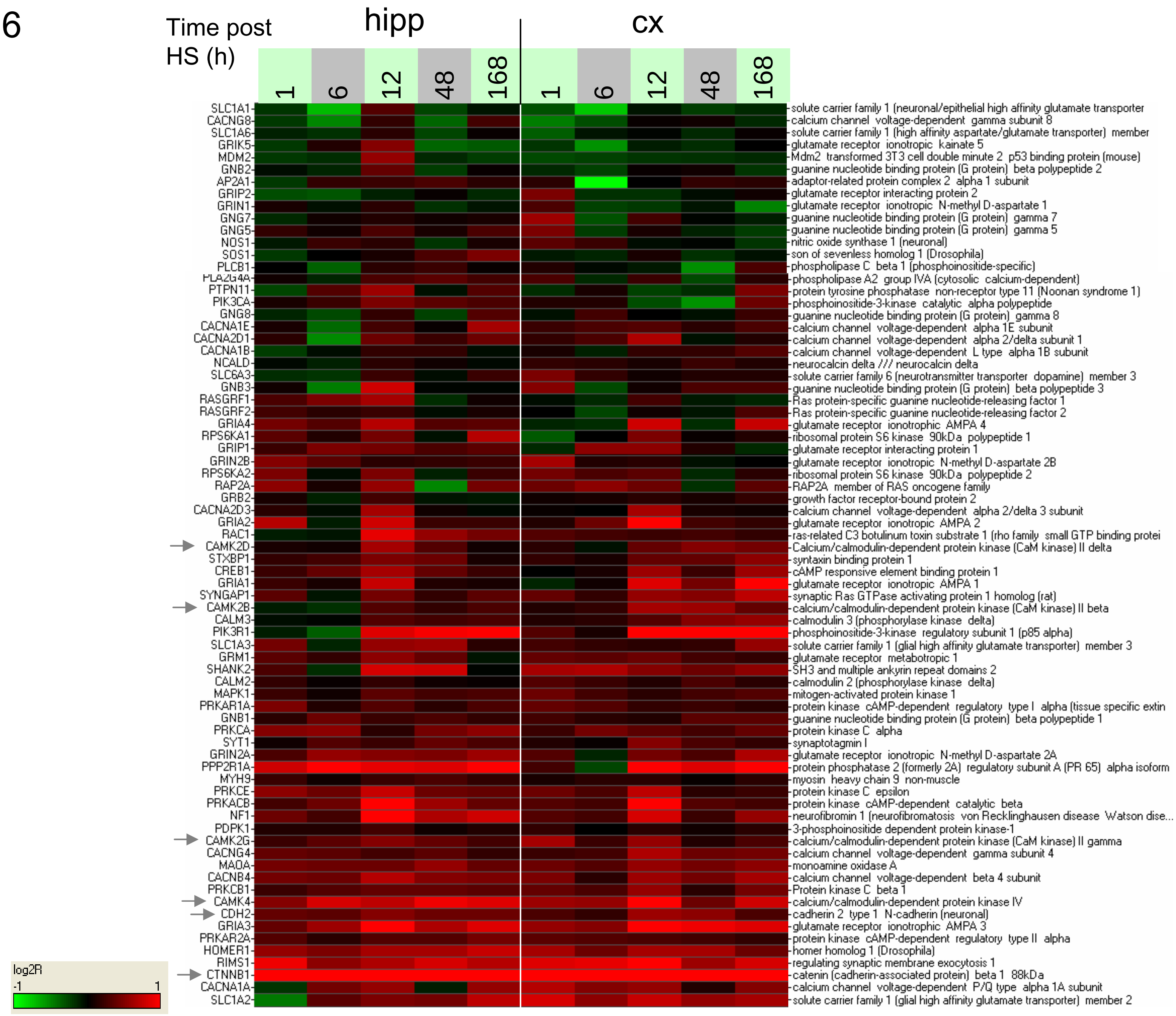

Figure S17

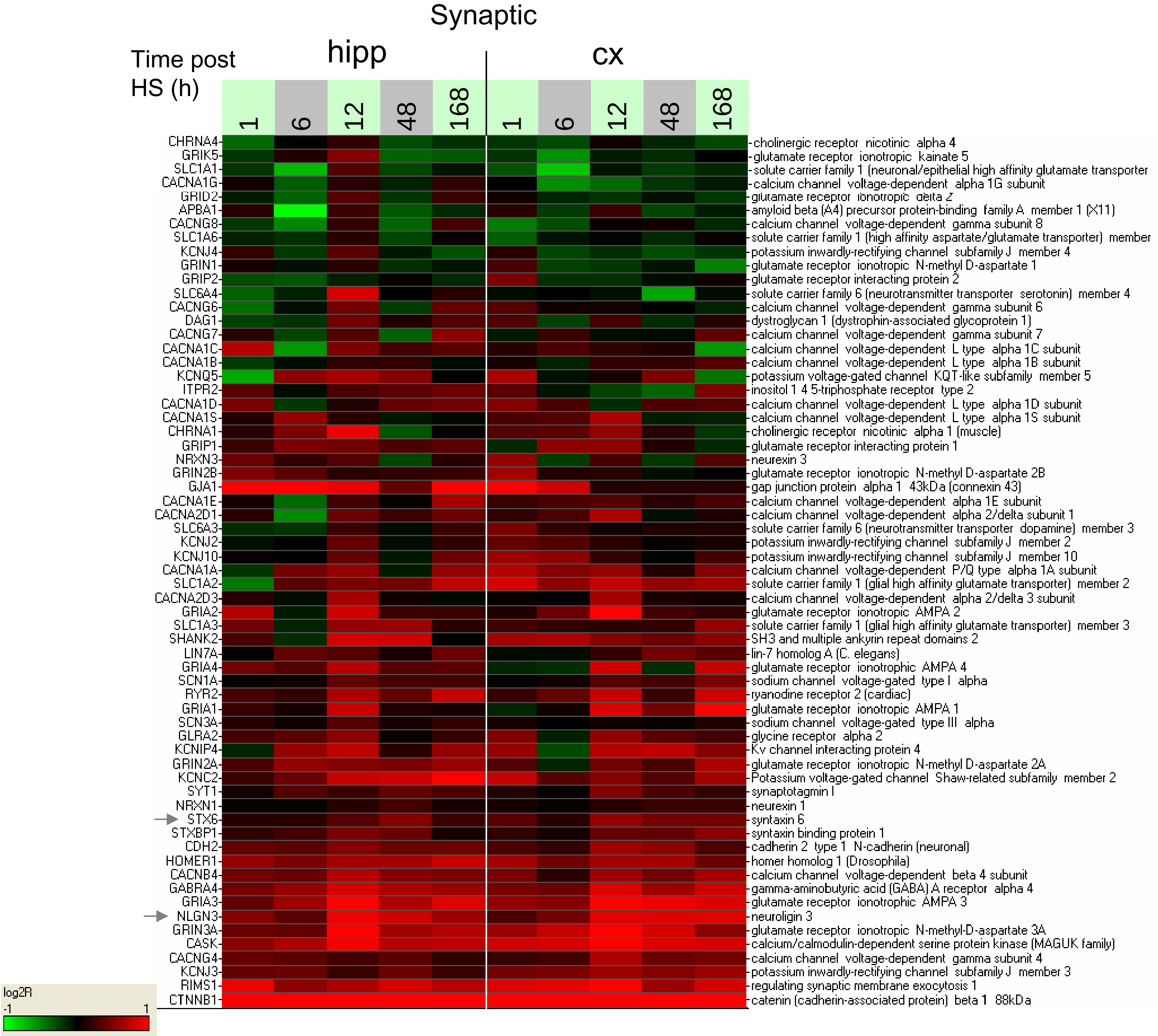
Synaptic
Time post HS (h)
hipp
cx
1
6
12
48
168
1
6
12
48
168
CHRNA4 - cholinergic receptor nicotinic alpha 4
GRIK5 - glutamate receptor ionotropic kainate 5
SLC1A1 - solute carrier family 1 (neuronal/epithelial high affinity glutamate transporter
CACNA1G - calcium channel voltage-dependent alpha 1G subunit
GRID2 - glutamate receptor ionotropic delta 2
APBA1 - amyloid beta (A4) precursor protein-binding family A member 1 (X11)
CACNG8 - calcium channel voltage-dependent gamma subunit 8
SLC1A6 - solute carrier family 1 (high affinity aspartate/glutamate transporter) member
KCNJ4 - potassium inwardly-rectifying channel subfamily J member 4
GRIN1 - glutamate receptor ionotropic N-methyl D-aspartate 1
GRIP2 - glutamate receptor interacting protein 2
SLC6A4 - solute carrier family 6 (neurotransmitter transporter serotonin) member 4
CACNG6 - calcium channel voltage-dependent gamma subunit 6
DAG1 - dystroglycan 1 (dystrophin-associated glycoprotein 1)
CACNG7 - calcium channel voltage-dependent gamma subunit 7
CACNA1C - calcium channel voltage-dependent L type alpha 1C subunit
CACNA1B - calcium channel voltage-dependent L type alpha 1B subunit
KCNQ5 - potassium voltage-gated channel KQT-like subfamily member 5
ITPR2 - inositol 1 4 5-triphosphate receptor type 2
CACNA1D - calcium channel voltage-dependent L type alpha 1D subunit
CACNA1S - calcium channel voltage-dependent L type alpha 1S subunit
CHRNA1 - cholinergic receptor nicotinic alpha 1 (muscle)
GRIP1 - glutamate receptor interacting protein 1
NRXN3 - neurexin 3
GRIN2B - glutamate receptor ionotropic N-methyl D-aspartate 2B
GJA1 - gap junction protein alpha 1 43kDa (connexin 43)
CACNA1E - calcium channel voltage-dependent alpha 1E subunit
CACNA2D1 - calcium channel voltage-dependent alpha 2/delta subunit 1
SLC6A3 - solute carrier family 6 (neurotransmitter transporter dopamine) member 3
KCNJ2 - potassium inwardly-rectifying channel subfamily J member 2
KCNJ10 - potassium inwardly-rectifying channel subfamily J member 10
CACNA1A - calcium channel voltage-dependent P/Q type alpha 1A subunit
SLC1A2 - solute carrier family 1 (glial high affinity glutamate transporter) member 2
CACNA2D3 - calcium channel voltage-dependent alpha 2/delta 3 subunit
GRIA2 - glutamate receptor ionotropic AMPA 2
SLC1A3 - solute carrier family 1 (glial high affinity glutamate transporter) member 3
SHANK2 - SH3 and multiple ankyrin repeat domains 2
LIN7A - lin-7 homolog A (C. elegans)
GRIA4 - glutamate receptor ionotrophic AMPA 4
SCN1A - sodium channel voltage-gated type I alpha
RYR2 - ryanodine receptor 2 (cardiac)
GRIA1 - glutamate receptor ionotropic AMPA 1
SCN3A - sodium channel voltage-gated type III alpha
GLRA2 - glycine receptor alpha 2
KCNIP4 - Kv channel interacting protein 4
GRIN2A - glutamate receptor ionotropic N-methyl D-aspartate 2A
KCNC2 - Potassium voltage-gated channel Shaw-related subfamily member 2
SYT1 - synaptotagmin I
NRXN1 - neurexin 1
STX6 - syntaxin 6
STXBP1 - syntaxin binding protein 1
CDH2 - cadherin 2 type 1 N-cadherin (neuronal)
HOMER1 - homer homolog 1 (Drosophila)
CACNB4 - calcium channel voltage-dependent beta 4 subunit
GABRA4 - gamma-aminobutyric acid (GABA) A receptor alpha 4
GRIA3 - glutamate receptor ionotrophic AMPA 3
NLGN3 - neuroligin 3
GRIN3A - glutamate receptor ionotropic N-methyl-D-aspartate 3A
CASK - calcium/calmodulin-dependent serine protein kinase (MAGUK family)
CACNG4 - calcium channel voltage-dependent gamma subunit 4
KCNJ3 - potassium inwardly-rectifying channel subfamily J member 3
RIMS1 - regulating synaptic membrane exocytosis 1
CTNNB1 - catenin (cadherin-associated protein) beta 1 88kDa
log2R
-1
1

Figure S18

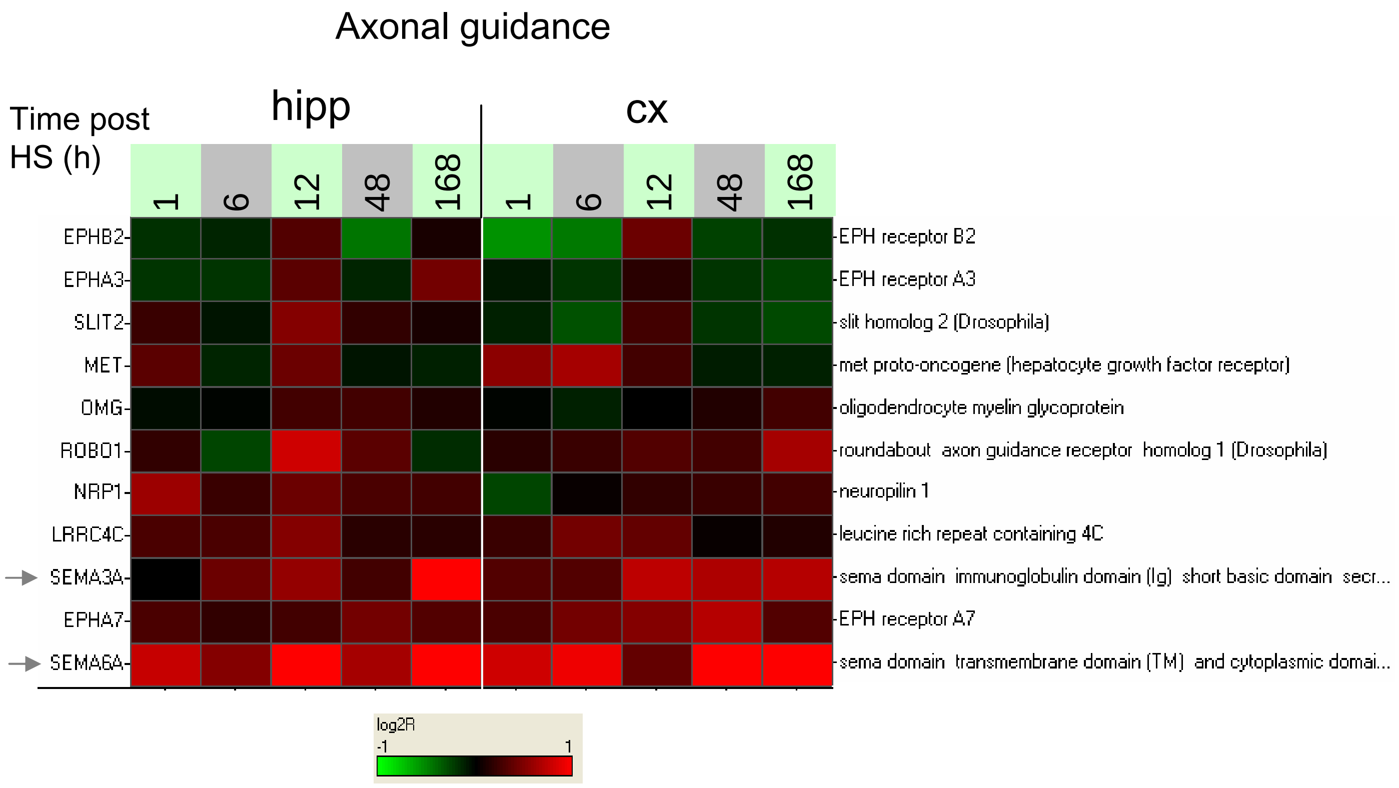
Axonal guidance
Time post HS (h)
hipp
cx
1
6
12
48
168
1
6
12
48
168
EPHB2
EPHA3
SLIT2
MET
OMG
ROBO1
NRP1
LRRC4C
SEMA3A
EPHA7
SEMA6A
EPH receptor B2
EPH receptor A3
slit homolog 2 (Drosophila)
met proto-oncogene (hepatocyte growth factor receptor)
oligodendrocyte myelin glycoprotein
roundabout axon guidance receptor homolog 1 (Drosophila)
neuropilin 1
leucine rich repeat containing 4C
sema domain immunoglobulin domain (Ig) short basic domain secr...
EPH receptor A7
sema domain transmembrane domain (TM) and cytoplasmic domai...
log2R
-1
1

Figure S19

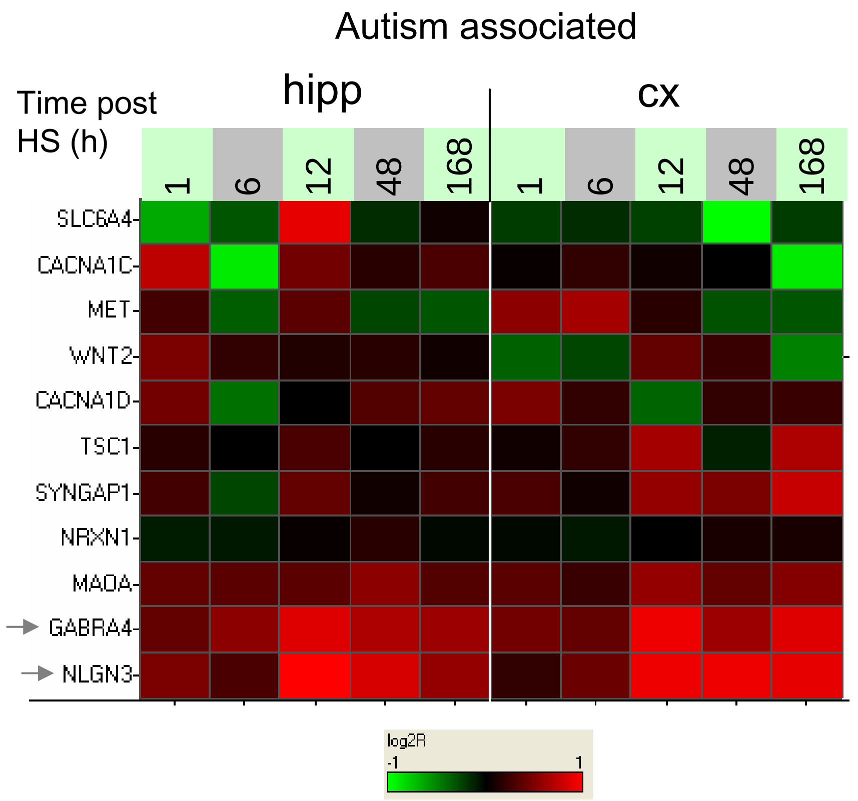

Autism associated
Time post HS (h)
hipp
cx
1
6
12
48
168
1
6
12
48
168
SLC6A4
CACNA1C
MET
WNT2
CACNA1D
TSC1
SYNGAP1
NRXN1
MAOA
GABRA4
NLGN3
log2R
-1
1

Figure S20

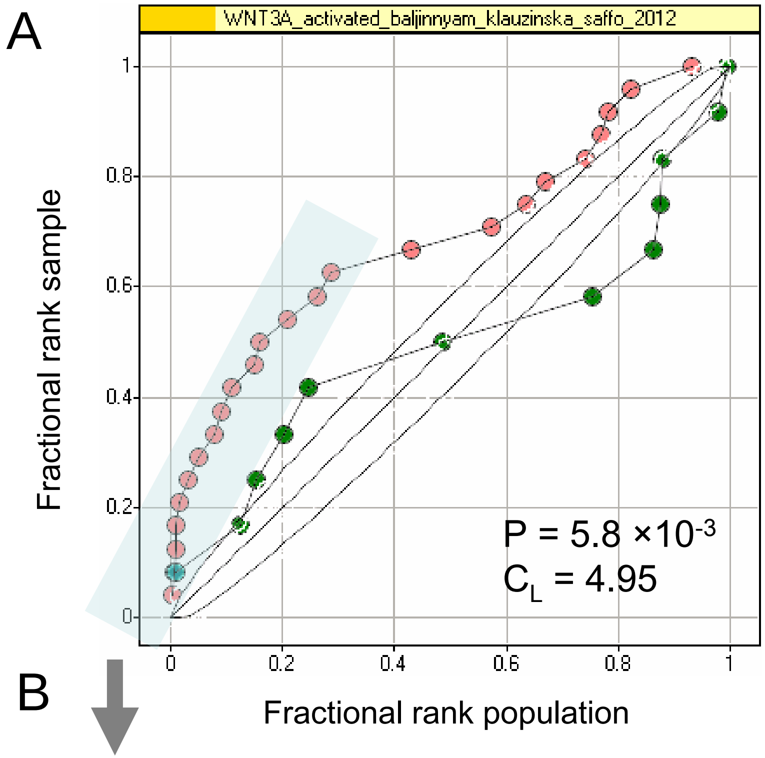

Leading-edge genes (15)

#Gene	description	Ratio	P-value
KLF5	Kruppel-like factor 5 (intestinal)	2.282	0.0446
ENPP2	ectonucleotide pyrophosphatase/phosphodiesterase 2	2.158	0.203
CHRNA1	cholinergic receptor nicotinic alpha 1 (muscle)	1.895	0.00207
RND3	Rho family GTPase 3	1.888	0.00237
IRS1	insulin receptor substrate 1	1.785	0.0034
TCF4	transcription factor 4	1.579	0.0061
AXIN2	axin 2 (conductin axil)	1.482	0.00693
AHR	aryl hydrocarbon receptor	1.396	0.248
TGFBR2	transforming growth factor beta receptor II (70/80kDa)	1.356	0.168
TEC	tec protein tyrosine kinase	1.322	0.147
CTGF	connective tissue growth factor	1.253	0.227
CCND1	cyclin D1	1.238	0.091
SPRY1	sprouty homolog 1 antagonist of FGF signaling (Drosc	1.180	0.277
IGF1	insulin-like growth factor 1 (somatomedin C)	1.133	0.0652
TGFB2	Transforming growth factor beta 2	1.115	0.178

Figure S21

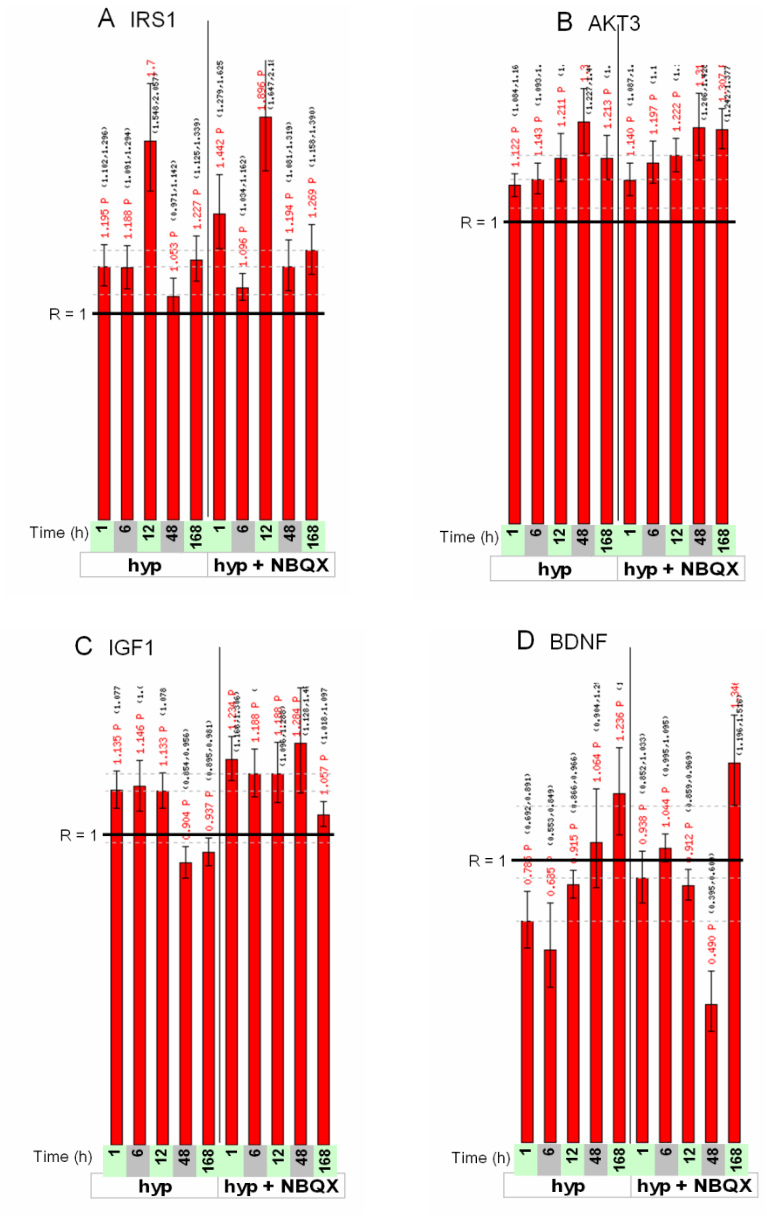

Figure S22

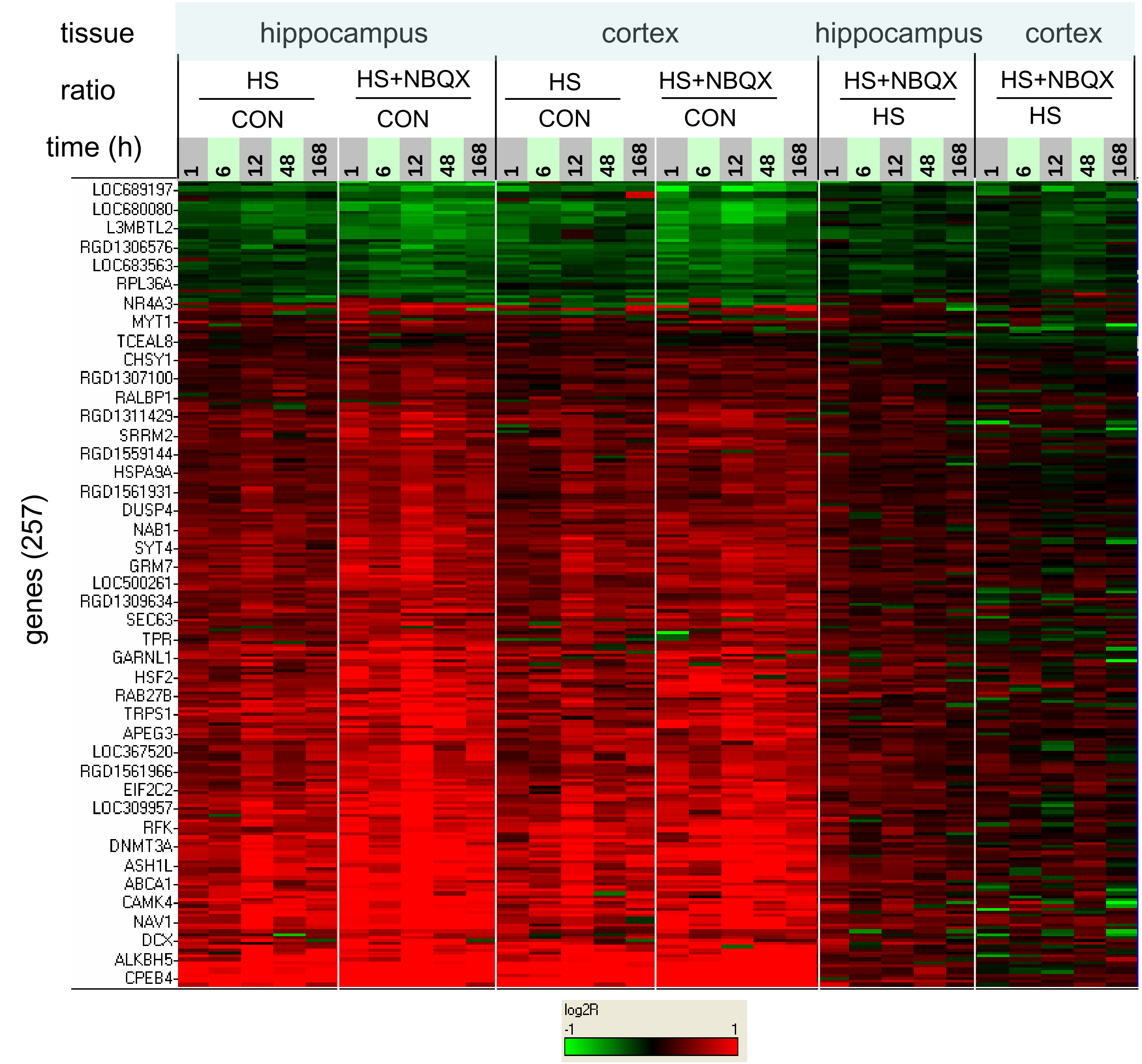

tissue
hippocampus
cortex
hippocampus
cortex
ratio
HS / CON
HS+NBQX / CON
HS / CON
HS+NBQX / CON
HS+NBQX / HS
HS+NBQX / HS
time (h)
1 6 12 48 168
genes (257)
LOC689197
LOC680080
L3MBTL2
RGD1306576
LOC683563
RPL36A
NR4A3
MYT1
TCEAL8
CHSY1
RGD1307100
RALBP1
RGD1311429
SRRM2
RGD1559144
HSPA9A
RGD1561931
DUSP4
NAB1
SYT4
GRM7
LOC500261
RGD1309634
SEC63
TPR
GARNL1
HSF2
RAB27B
TRPS1
APEG3
LOC367520
RGD1561966
EIF2C2
LOC309957
RFK
DNMT3A
ASH1L
ABCA1
CAMK4
NAV1
DCX
ALKBH5
CPEB4
log2R
-1
1

Figure S23

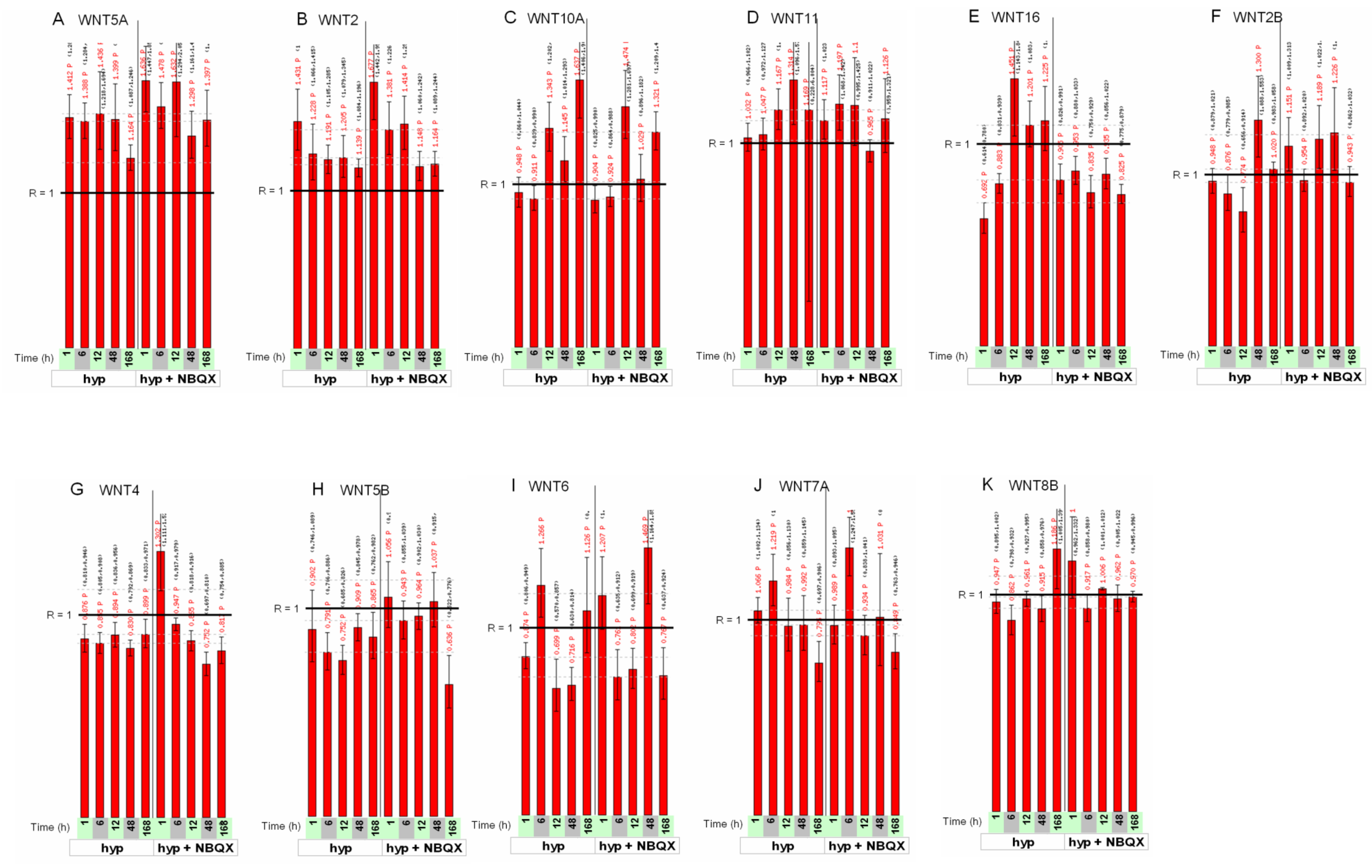
A WNT5A
B WNT2
C WNT10A
D WNT11
E WNT16
F WNT2B
G WNT4
H WNT5B
I WNT6
J WNT7A
K WNT8B
R = 1
Time (h)
1
6
12
48
168
hyp
hyp + NBQX
